# Synchrotron radiation micro-computed tomography of the small-spotted catshark embryonic development (Chondrichthyes: *Scyliorhinus canicula*)

**DOI:** 10.1093/gigascience/giag054

**Published:** 2026-05-07

**Authors:** Elio Escamilla-Vega, Ann-Katrin Koch, Louk W G Seton, Andrea P Murillo-Rincón, Stella Kyomen, Jörg U Hammel, Timo Moritz, Markéta Kaucká

**Affiliations:** Max Planck Institute for Evolutionary Biology, August-Thienemann-Str. 2, 24306 Plön, Germany; Ocean Museum Germany, Katharinenberg 14–20, 18439 Stralsund, Germany; Institute of Biosciences, University of Rostock, Albert–Einstein–Str. 3, 18059 Rostock, Germany; Max Planck Institute for Evolutionary Biology, August-Thienemann-Str. 2, 24306 Plön, Germany; Max Planck Institute for Evolutionary Biology, August-Thienemann-Str. 2, 24306 Plön, Germany; Max Planck Institute for Evolutionary Biology, August-Thienemann-Str. 2, 24306 Plön, Germany; Institute of Materials Physics, Helmholtz-Zentrum Hereon, Max-Planck-Str. 1, 21502 Geesthacht, Germany; Ocean Museum Germany, Katharinenberg 14–20, 18439 Stralsund, Germany; Leibniz Institute for the Analysis of Biodiversity Change, Martin-Luther-King-Platz 3, D-20146 Hamburg, Germany; Max Planck Institute for Evolutionary Biology, August-Thienemann-Str. 2, 24306 Plön, Germany

**Keywords:** synchrotron radiation micro-computed tomography, X-rays, tissue contrast, cartilaginous fish, Chondrichthyes, small-spotted catshark, *Scyliorhinus canicula*, shark ontogeny, evo-devo

## Abstract

**Background:**

Sharks occupy a key position on vertebrate phylogeny, making them essential for understanding the early origins of jawed vertebrates (gnathostomes) and the functional adaptation of vertebrate traits like jaws or complex sensory systems. As such, sharks are important model organisms in evolutionary developmental biology (evo-devo), but sparse data and limited availability of samples hinder their inclusion in contemporary evo-devo research. The knowledge of their distinctive morphology will not only shed light on the anatomical architecture and physiology of basal living gnathostomes but also reveal the evolutionary divergence of developmental processes that establish the foundational vertebrate blueprint.

**Findings:**

We performed synchrotron radiation micro-computed tomography (SRµCT) scanning of the small-spotted catshark (*Scyliorhinus canicula*) embryonic development, spanning from gastrulation (Stage 12) to late organogenesis (Stage 31), enhanced by tissue contrasting with phosphotungstic acid. We obtained 36 whole-embryo scans that encompass the formation of key embryonic structures, such as sensory organs, fins, muscles, and skeletal elements. The achieved resolution allows for segmentation of all tissue types and both internal and external structures.

**Conclusions:**

We present a comprehensive dataset of 4D high-resolution SRµCT of the small-spotted catshark embryonic development. The dataset spans consecutive embryonic stages, allowing the reconstruction and morphometric analyses of tissues, organs, and structures, along with the tracking of their development. The deposited data are publicly available and provide a valuable resource for comparative research, additionally allowing the identification of conserved and derived developmental processes and features and understanding the evolution of vertebrates.

## Background

Vertebrate development represents a series of tightly regulated steps that orchestrate the formation of all body structures and their functional integration. While many fundamental processes, such as axis formation, segmentation, and organ primordia patterning, are shared across taxa, there are also significant differences that result in the remarkable morphological diversity of vertebrates [1–4]. The differences in embryogenesis reflect both phylogenetic divergence and species-specific adaptations, such as ecological and reproductive pressures [5–7]. Comparative embryology across a broad range of vertebrates is therefore essential for disentangling ancestral traits from lineage-specific innovations and for understanding the evolution of developmental processes that generate morphological diversity.

In the past century, a limited repertoire of model vertebrate organisms has been used for comparative embryology research. These studies provided a solid foundation for our understanding of vertebrate development and identified events, structures, and embryonic stages where the evolutionary divergence of developmental programs arises. Mouse, chicken, clawed frogs, and zebrafish are among the most commonly used vertebrate species in developmental biology [8, 9]. More recently, the taxon sampling has expanded with the introduction of emerging experimental organisms like bats [10, 11], ostriches [12, 13], lizards [14, 15], and non-teleost fishes [16, 17], which has enabled comparative embryology to provide valuable insights into the mechanisms underlying the evolution of vertebrate developmental programs. All these species belong to the same superclass of bony vertebrates (Osteichthyes), thus not covering the entire vertebrate subphylum. Notable progress in breeding and maintaining jawless vertebrates (cyclostomes—lamprey and hagfish) [18], has allowed their broader inclusion in modern evo-devo [19–21]. In addition, cartilaginous fishes (Chondrichthyes) such as elephant sharks (*Callorhinchus milii*) and bamboo sharks (*Chiloscyllium* sp.) are also emerging as key research species [22–24]. However, the vast majority of vertebrate evo-devo research remains mostly restricted to representatives of Osteichthyes, leaving cyclostomes and Chondrichthyes comparatively underrepresented.

Chondrichthyes are the sister group of Osteichthyes and represent 1 of the 2 lineages of living jawed vertebrates (gnathostomes). This morphologically diverse group of animals, which diverged from a common ancestor about 420 million years ago, is divided into 2 classes: Elasmobranchii (sharks, rays, skates, and sawfish) and Holocephali (chimaeras) [25]. Chondrichthyans are primarily known for having a cartilaginous endoskeleton throughout their lives, and the loss of perichondral, endochondral, and most dermal bones [26–29]. These traits render them important models for the research of skeletal tissue evolution. Their unique features and phylogenetic position render Chondrichthyes a valuable taxon in vertebrate comparative evo-devo research [30–33]. However, a broader inclusion of Chondrichthyes in contemporary embryological research remains limited due to several inherent challenges. The difficulty in obtaining sufficient numbers of embryos resulting from reduced mating in captivity [34–36], restricted habitats (primarily chimaeras), seasonal breeding with low fecundity [37], reproductive modes that often require the sacrifice of adult females [38, 39], high extinction risks [40], long generation times (~175 days in the small-spotted catshark) [41], and the challenges associated with maintaining these species in aquarium settings, hinder their establishment as laboratory model system. Nevertheless, despite these limitations, 2 oviparous species have emerged as promising model organisms in evo-devo research: the little skate (*Leucoraja erinacea*) and the small-spotted catshark (*Scyliorhinus canicula*) [36]. The small-spotted catshark is an abundant, non-endangered species commonly kept in aquaria, capable of mating in captivity and laying eggs throughout most of the year. Moreover, this shark species is well suited for modern genomic approaches, supported by a chromosomal-level genome assembly and numerous transcriptomic resources [42–45], although genome editing remains a major challenge not only in the small-spotted catshark but generally across chondrichthyans.

Non-destructive three-dimensional imaging techniques, such as confocal laser scanning microscopy combined with immunofluorescence and *in situ* hybridization, have been used to visualize and follow the development of specific anatomical structures [46–49]. However, applying these techniques to non-model organisms remains challenging, for instance, due to the limited availability of specific and effective antibodies for immunostaining. Additionally, although confocal microscopy remains as one of the most popular and widespread 3D imaging techniques, it is limited by the need for fluorescent labeling of the structures of interest, and insufficient light penetration becomes problematic in larger samples. To overcome these limitations and image larger samples, we explored the use of X-ray-based methods, which allow imaging of thick samples at the centimeter scale [42].

Micro-computed tomography (µCT) is a frequently used approach in developmental biology [50–52]. Samples are irradiated with X-rays from multiple angles, obtaining 2D projections, which are later computationally reconstructed into 3D models. Even though absorption-based µCT provides an excellent way to obtain high-resolution 3D data, it also has 2 main limitations. The first one is its inability to effectively differentiate soft tissues due to insufficient differences in their X-ray attenuation coefficients (phase-contrast imaging may overcome this constraint under appropriate conditions). As a result, only dense structures like bones, teeth, and scales are readily visible in the reconstructed tomographic images [53, 54]. This limitation can be overcome by using contrasting agents, which are differentially absorbed by soft tissues, allowing their visualization in the final images [55]. Various contrast-enhancing agents have been tested, including iodine [51, 56–58], ruthenium red [59], and phosphotungstic acid (PTA) [50, 60, 61], each with distinct tissue-specific absorption rates and, thus, contrasting abilities (e.g., PTA does not stain cartilage) [62–65]. The second, unavoidable limitation arises from the physical properties of conventional laboratory-based µCT systems. These conventional scanners are prone to beam hardening artifacts, low signal-to-noise ratios, and resolution constraints, particularly when imaging smaller samples, which can make fine structures indistinguishable in the final images [66, 67]. In synchrotron facilities, the high-energy nearly parallel monochromatic X-ray beam makes it possible to obtain high spatial resolution (i.e., micron scale) images of macroscopic samples (centimeter range) with improved quality and reduced imaging times [68, 69]. Synchrotron radiation micro-computed tomography (SRµCT) combined with iodine-based contrast enhancement was previously employed to reconstruct and characterize the sensory organs in a pre-hatching small-spotted catshark embryo [42], highlighting the potential of this imaging technology to study chondrichthyan development in great detail.

Here, we took advantage of SRµCT combined with PTA-contrasting to generate a high-resolution 4D (volume and time) atlas of the small-spotted catshark development and extend the available information from pre-hatching stages [42]. SRµCT is particularly valuable for studying shark embryos, which are non-transparent and thick and difficult to image using optical techniques. This strategy allowed the manual segmentation of a wide range of embryonic structures, and in turn, the reconstruction of the developmental progression that shapes the shark body plan and anatomical features. We provide the raw reconstructed tomographic slices together with the pre-processed files. The provided dataset will benefit researchers interested in Chondrichthyes embryology and comparative evo-devo studies, addressing a wide spectrum of research questions, without requiring access to new specimens or museum-preserved samples.

## Methods

### Small-spotted catshark sample preparation

**Figure 1:**
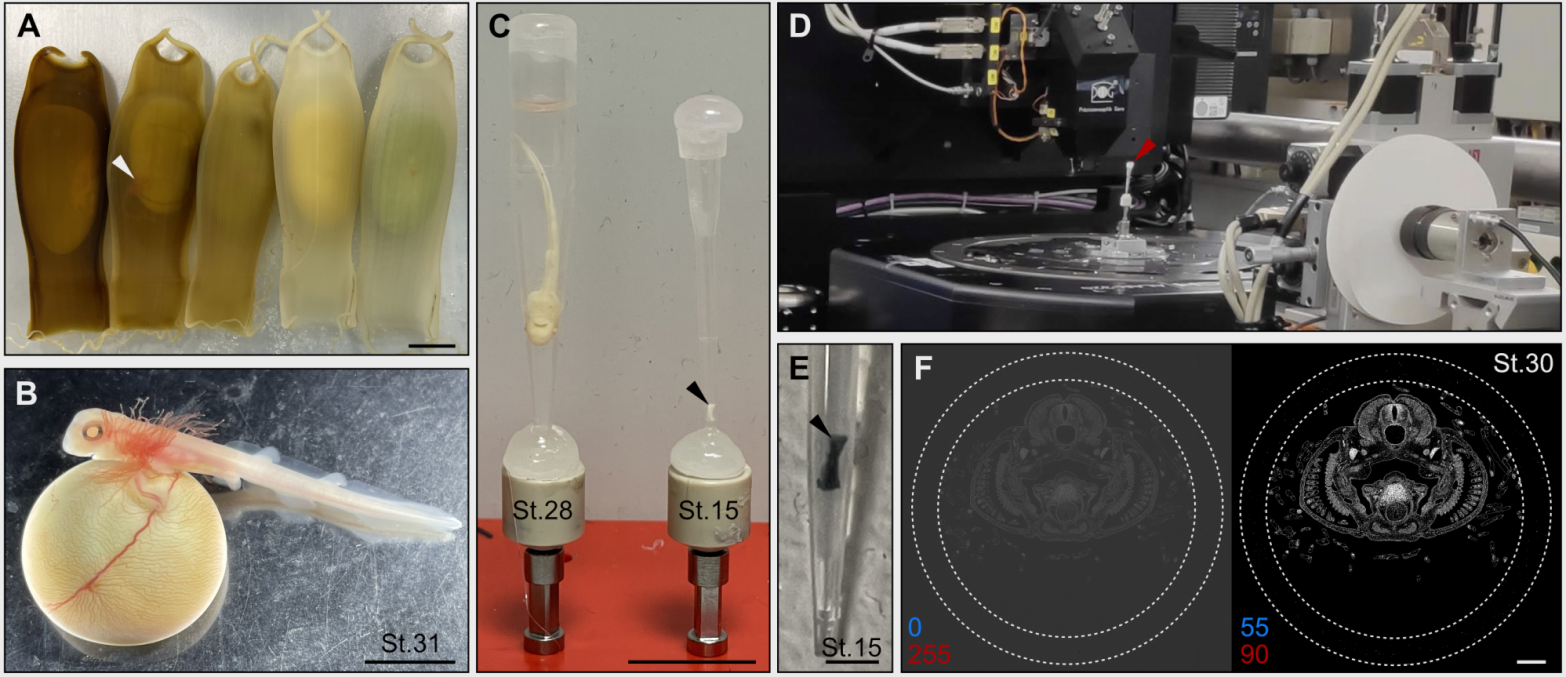
Experimental setup for SRµCT of small-spotted catshark embryos. (A) Fertilized small-spotted catshark eggs. Small-spotted catshark egg cases display a broad range of coloration from light beige to dark brown [92, 93]. The underlying causes of this phenotypic variation remain unknown, although genetic factors have been suggested as contributing influences [92]. Importantly, egg case pigmentation does not affect embryo development [93]. White arrowhead indicates an embryo at approximately St.28 within the eggcase. Scale bar: 1 cm. (B) St.31 small-spotted catshark embryo, attached to its yolk sac and removed from the egg case. Scale bar: 1 cm. (C) PTA-contrasted small-spotted catshark embryos at 2 developmental stages: St.28 (left), and St.15 (right, indicated by black arrowhead). The embryos are mounted in plastic pipette tips and glued to standardized holders. Scale bar: 1 cm. (D) Experimental setup at the beamline P05 at PETRA III. Red arrowhead indicates the sample positioned in the center of the stage prior to imaging. (E) St.15 small-spotted catshark embryo from Fig. 1C immediately after scanning. Scale bar: 1.5 mm. (F) Raw tomographic slice of PTA-contrasted St.30 small-spotted catshark embryo (left) and the same tomographic slice after pre-processing in ImageJ/Fiji (right). The dotted white circles indicate the pipette tip containing the embryo. Thresholds for the adjustment of brightness and contrast are shown in blue (low-intensity value) and red (high-intensity value), with the original values shown for the unprocessed image. Scale bar: 500 µm.

Fertilized small-spotted catshark eggs were obtained from Ozeaneum (Stralsund, Germany) and Sea Life Berlin (Berlin, Germany) (Fig. 1A), opened using sharp dissecting scissors, and the whole content of the eggs was placed in glass Petri dishes with oxygenated seawater (Fig. 1B). The embryos were then carefully separated from the yolk under a stereomicroscope, staged according to Ballard and colleagues [41], euthanized by a tricaine (ethyl-3-aminobenzoate methanesulfonate, Merck, E10521) overdose (0.04% tricaine in seawater) [70, 71], and fixed in freshly prepared 4% paraformaldehyde (PFA) in 1X Dulbecco’s phosphate buffered saline (PBS—Sigma D5652) for 24 h at 4°C with gentle rotation. Following fixation, embryos were washed with PBS and subsequently dehydrated in increasing ethanol (Fisher Chemical E/0650DF/17) concentrations in PBS (30%, 50%, 75%, and 90%) for 24 h each on slow rotation. The slow rotation, together with the long incubation in ethanol, ensures even dehydration while minimizing tissue shrinking and preserving the morphological features. Embryos were then incubated in 1.5% PTA (Sigma P4006) in 90% methanol (Fisher Chemical M/4056/17) to enhance the contrast and visualize soft tissues [50, 60, 61, 63, 64]. The PTA contrasting solution was replaced twice a week. The staining times for each developmental stage are detailed in Supplementary Table 1. Once the embryos were saturated with the contrasting solution, they were washed twice in 100% ethanol for 24 h to remove any PTA excess and stored in 100% ethanol until scanning.

### SRµCT image acquisition

PTA-contrasted small-spotted catshark embryos were placed inside bottom-sealed plastic pipette tips filled with 100% ethanol (Fig. 1C). The pipette tips were bottom-sealed using UV-light sensitive resin (UVR-100, MOCOBO) and their volume was chosen with respect to the size of the embryo (Fisher Scientific—10 µl 0030073371, 200 µl 0030073436, 1,000 µl 0030000927), with larger embryos placed in larger tips and vice versa. The conical shape of the pipette tips keeps the embryos in place and prevents movements during scanning. Once the embryos were fixed inside the pipette tips, they were carefully aligned as straight as possible using fine forceps in the center to avoid scanning artifacts. Subsequently, the tips were top-sealed using a hot glue gun to avoid drying of the samples during the scans, and glued to a standardized sample holder that fits in the rotation stage (Fig. 1C).

Attenuation-contrast SRµCT measurements on full acquisition mode were acquired at the Imaging Beamline P05 of the storage ring PETRA III (Deutsches Elektronen Synchrotron—DESY, Hamburg, Germany) operated by the Helmholtz-Zentrum Geesthacht [72] (Fig. 1D). A double crystal monochromator with 1,1,1-silicon crystals was used to set a photon energy of 20 keV. All embryos were imaged with the same photon energy (20 keV) and a sample-to-detector distance of 80 mm. 3001 projections equally spaced between 0° and 180° were obtained for each tomographic scan (angular step of 0.059°) using a custom-developed 20 MP CMOS camera system [73], custom-made lenses (Präzisions Optik Gera, Germany), and a 100 µm CdWO4 scintillator. To optimize scanning time while obtaining the highest possible resolution for each sample, embryos were divided in 2 groups depending on their size and imaged with a different field of view (FOV) and exposure time. Embryos ranging from St.12-25 were imaged with a FOV of 3.29 mm × 2.47 mm, with an exposure time of 280 ms. Embryos ranging from St.26-31 were imaged with a FOV of 6.57 mm × 2.70 mm, with an exposure time of 80 ms. Multiscan vertical tiling was applied depending on embryo length to cover the whole specimen, with 16 tiles for the largest embryo (St.31). The scanning time for 1 tile was 16.4 min and 4.7 min for each FOV, respectively. Interestingly, the interaction between the X-ray radiation and the PTA contrasting agent causes the samples to turn temporarily blue after scanning (Fig. 1E). Detailed information regarding the SRµCT scanning parameters per sample can be found in Supplementary Table 2.

### Image reconstruction and pre-processing

Tomographic reconstruction of the 2D projections into 3D volumes was performed using a classical filtered back projection with a 2-fold binning based on a custom reconstruction pipeline implemented in MATLAB and the Astra Toolbox [74–77] (Fig. 1F). The reconstruction pipeline is deposited in GitHub [78]. The resulting reconstructed 3D images were saved as tiff. image stacks with an effective isotropic voxel size of 1.28 and 2.57 µm for each FOV, respectively. For samples requiring multiple vertical scans, the resulting tiles were stitched together using the same custom-designed algorithm. Manual adjustment of the stitched tiles was required to correct for minor misalignment artifacts observed in some samples. The final reconstructed and stitched files ranged between 46.8 and 381 GB. In total, we generated 5.04 TB of 3D data for further analysis.

Due to the large size of the reconstructed files, additional pre-processing was required before image analysis or manual segmentations could be performed. The 2D reconstructed stacks were first loaded into ImageJ/Fiji 2.9.0 [79] and cropped to retain only the embryos while removing the surrounding empty space. Afterward, the brightness and contrast were adjusted using the Brightness/Contrast function to refine the range of color values and enhance visualization of the embryonic structures [80] (Fig. 1F and Supplementary Fig. 1). Finally, images were converted from 32-bit into 8-bit format (Fig. 1F). By performing this series of transformations, a reconstructed stack of 46.8 GB reduced its size to ~1.3 GB. While the pre-processing steps were typically sufficient to reduce the size of most files, larger samples (St.25–31) required additional resampling to further reduce loading times and ensure efficient segmentations. Although resampling decreased the number of pixels and, thus, reduced image quality, the obtained pre-processed images remained of high quality and resolution.

### Manual segmentation of embryonic structures

**Figure 2:**
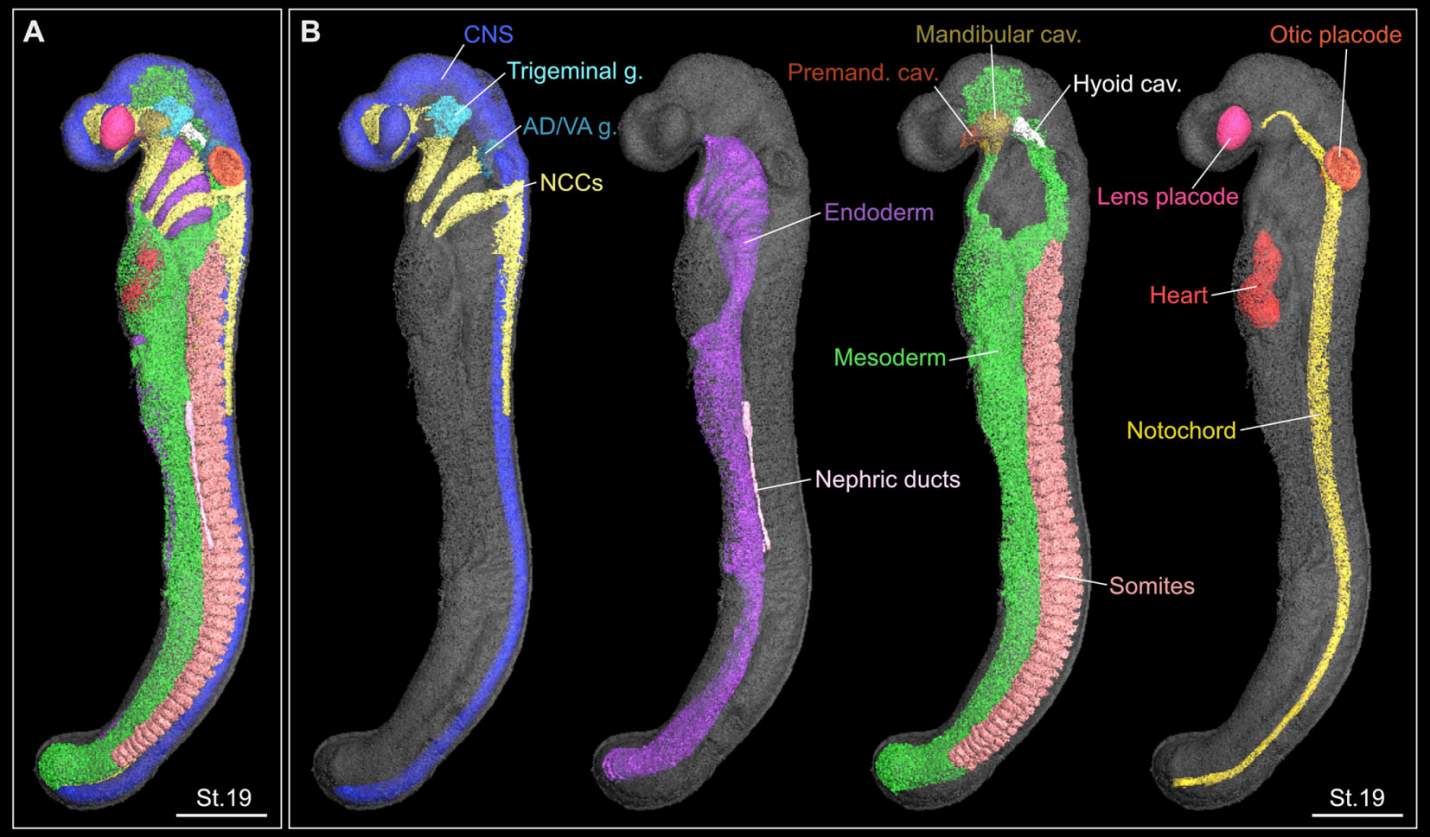
3D reconstruction of a fully segmented St.19 small-spotted catshark embryo. (A) 3D reconstruction of all segmented structures together. Note that some structures may not be visible in this model because they are located beneath other anatomical structures. Scale bar: 500 µm. (B) 3D reconstructions of segmented structures are presented separately for better visibility of individual organs and tissues. Scale bar: 500 µm. AD/VA, acousticofacial; cav, cavity; CNS, central nervous system; g, ganglia; NCCs, neural crest cells; Premand, premandibular.

Based on the pre-processed reconstructed SRµCT images, the different embryonic structures of the developing small-spotted catshark embryos were manually segmented using the interpolation and wrap functions from Avizo3D Pro Software (Thermo Fisher Scientific, Konrad-Zuse-Zentrum, Berlin, Germany) (Fig. 2). With the interpolation function, the experienced operator manually segmented every third slice on the same orthogonal projection, and the rest was automatically calculated by linear interpolation between the adjacent manually segmented slices. The interpolation tool was particularly useful when changes from slice-to-slice are small and progressive, like for the brain or ectodermal placodes. With the wrap selection function, the operator first created a scaffold by manually segmenting slices in the 3 orthogonal projections (XY, XZ, YZ) and then an algorithm automatically computed the remaining slices. The wrap tool was especially helpful for structures with more complex 3-dimensional shapes, like the somites. These tools considerably helped reduce the workload and increased the segmentation speed without impacting the accuracy [81], particularly important for large datasets such as the one provided here. Nonetheless, manual fine-tuning was necessary to ensure the accuracy of the segmentation in complex and fine structures. The manual segmentation of the individual embryonic elements took 1–5 days per sample for the smaller embryos (St.12–20) and up to 2 weeks for larger ones with more complex anatomy (St.21–31). Smoothing of 3D renderings was performed to reduce the staircase artifacts that appeared due to the manual segmentations of the structures in 2D slices. A slight tissue shrinkage was observed due to the overall tissue dehydration and PTA-contrasting enhancement (1.5% PTA in 90% methanol), which represents a known, unavoidable technical limitation of enhanced soft tissue contrast [65, 82, 83]. This effect was particularly evident in ectodermal structures starting at St.25 (Supplementary Fig. 2). However, despite the slight tissue shrinkage, the overall morphology and relative anatomical position of the different embryonic structures remain intact. Moreover, the relative order in which individual structures emerge along the developmental timeline is not changed, which is important for studies on developmental heterochrony.

### Validation of SRµCT data by confocal microscopy

**Figure 3:**
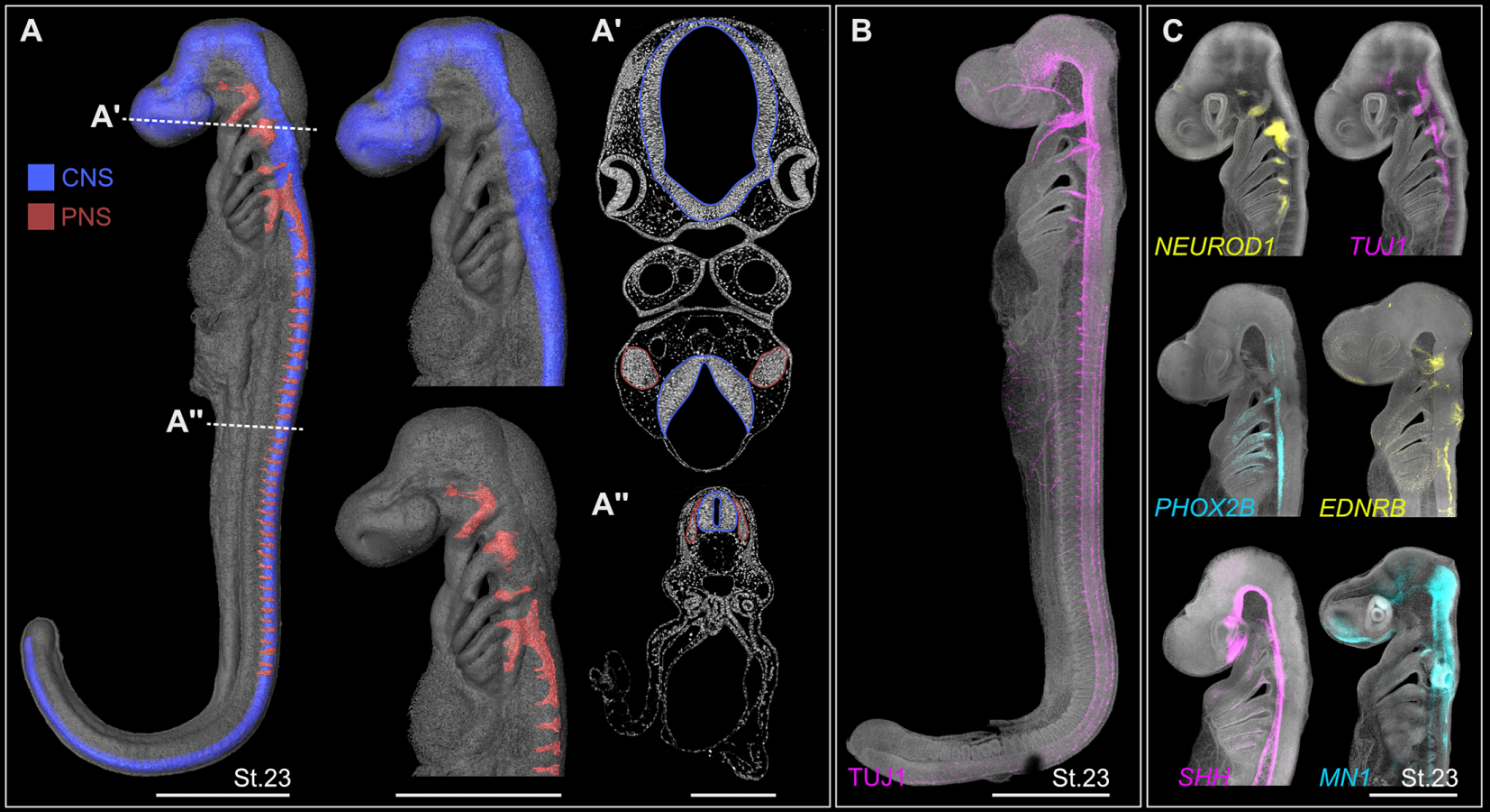
Combination of SRµCT with 3D confocal laser microscopy gene expression data. (A) 3D reconstruction of the developing small-spotted catshark nervous system at St.23. Blue 3D models represent the segmented central nervous system (CNS). Red 3D models represent the segmented peripheral nervous system (PNS). Scale bars: 1.5 mm. A′ and A″ indicate tomographic slices of the pre-processed SRµCT data used to reconstruct the nervous system. Scale bar: 250 µm. (B) TUJ1 immunofluorescence labeling the PNS and immature neurons of the CNS at St.23. Scale bar: 1.5 mm. (C) HCR *in situ* hybridization labeling different subpopulations within the developing nervous system at St.23. Scale bar: 1 mm.

The segmentation of different embryonic structures was performed based on morphological landmarks, contrast differences between adjacent tissues, and publicly available information on Chondrichthyes and vertebrate development. The segmented 3D reconstructions of the developing structures can be visualized simultaneously in the same model, which helps understand the spatial relationships between distinct tissues and organs (Fig. 3A). The segmented 3D models can be directly compared between consecutive developmental stages to understand the complex morphogenetic processes during embryogenesis, together with organ growth and shaping. This approach can be applied to virtually any of the developing structures. Moreover, the obtained 3D models can be additionally integrated with other popular non-destructive 3D techniques, such as confocal laser scanning microscopy with the use of immunofluorescence or HCR *in situ* hybridization (Fig. 3B, C). These 2 methods are essential tools in modern evo-devo research, enabling precise labeling of distinct cellular populations and anatomical structures within the developing embryo based on protein location and differential gene expression, respectively [84]. By combining the high-resolution SRµCT morphological data with fluorescence spatial gene expression mapping, we are able to establish a powerful framework to link genetic programs with tissue architecture and organismal morphology.

Briefly, following embryo collection and fixation, small-spotted catshark embryos were washed with PBS and subsequently dehydrated in increasing methanol (Fisher Chemical M/4056/17) steps (25%, 50%, 75%, and 100%) diluted in 0.1% PBST for 15 min each on slow rotation on ice. Subsequently, they were incubated in methanol for 1 h on slow rotation at room temperature and stored in methanol at −20°C untill further use. To reduce autofluorescence and improve the fluorescence signal, samples were bleached by incubation in Dent’s Bleach solution (1 volume Vaprox®—Steris PB006EUR; 2 volumes Dent’s Fix solution—80% methanol, 20% DMSO—Roth A994.1) overnight on slow rotation at 4°C. Afterwards, samples were washed 3 × 10 min in methanol and incubated overnight at 4°C in Dent’s Fix solution.

Immunofluorescence. Bleached embryos were rinsed 3 times in 0.1% PBST and subsequently washed 3 × 20 min in 0.1% PBST at 4°C to remove any traces of DMSO and methanol. Subsequently, they were incubated at room temperature for 5 days in primary antibody diluted in blocking solution composed of 20% DMSO and 5% donkey serum (Interchim UP77719A-K) in PBS. The following antibody was used to label the PNS and immature neurons of the CNS: mouse anti-Tuj1 (Promega G7121; 1:500). Following primary antibody incubation, samples were rinsed 3 times in 0.1% PBST, washed 3 × 20 min in 0.1% PBST, and incubated in secondary antibody at RT for 3 days diluted in blocking solution. The secondary antibody used was produced in donkey and conjugated with Alexa Fluor 647 (Thermo Fisher A31571; 1:1000). Nuclear stain was performed by incubating embryos overnight at 4°C in 1X DAPI (4′,6-diamidino-2-phenylindole; Thermo Fisher D21490) in PBST. Finally, samples were rinsed 3 times in 0.1% PBST, washed 3 × 20 min in 0.1% PBST, and cleared using BABB [85]. Whole-mount imaging was performed using glass-bottom imaging dishes (Eppendorf 0030740017) in a Zeiss LSM980 with Airyscan2 confocal microscope with the Plan-Apochromat 10x/0.45 M27 (Zeiss 420640-9900) objective. Final confocal images were processed and exported from ZEN 3.9 software.

HCR *in situ* hybridization. Bleached embryos were rehydrated in decreasing methanol series 15 min each (75%, 50%, 25%, 0%—in 0.1% PBST) at 4°C on slow rotation, post-fixed 15 min at room temperature in 4% PFA and pre-hybridized in 30% probe hybridization buffer for 30 min at 37°C. Once samples sank to the bottom of the tube, they were considered equilibrated in probe hybridization buffer and subsequently incubated overnight at 37°C in 2 pmol of HCR probes diluted in 30% probe hybridization buffer in a thermomixer (Eppendorf 5355) with gentle shaking (450 rpm). The following morning, samples were washed 4×15 min in 30% probe wash buffer at 37°C, 4×15 min in 0.1% 5X SSC-Tween (SSCT) at room temperature (20X SSC; Fisher BioReagents BP1325-4), and pre-amplified in HCR amplification buffer for 30 min. Once equilibrated, samples were incubated overnight at room temperature in 30 pmol of fluorescent-labeled hairpins diluted in amplification buffer, in the dark [84, 86]. On the following morning, samples were washed 4 × 15 min in 5X SSCT to remove excess hairpins and incubated overnight at 4°C in 1X DAPI in 5X SSCT to counterstain the nuclei. Finally, samples were washed 4 × 15 min in 5X SSCT, cleared using BABB, and imaged using a Zeiss LSM980 with Airyscan2 confocal microscope.

### Reuse potential

**Figure 4:**
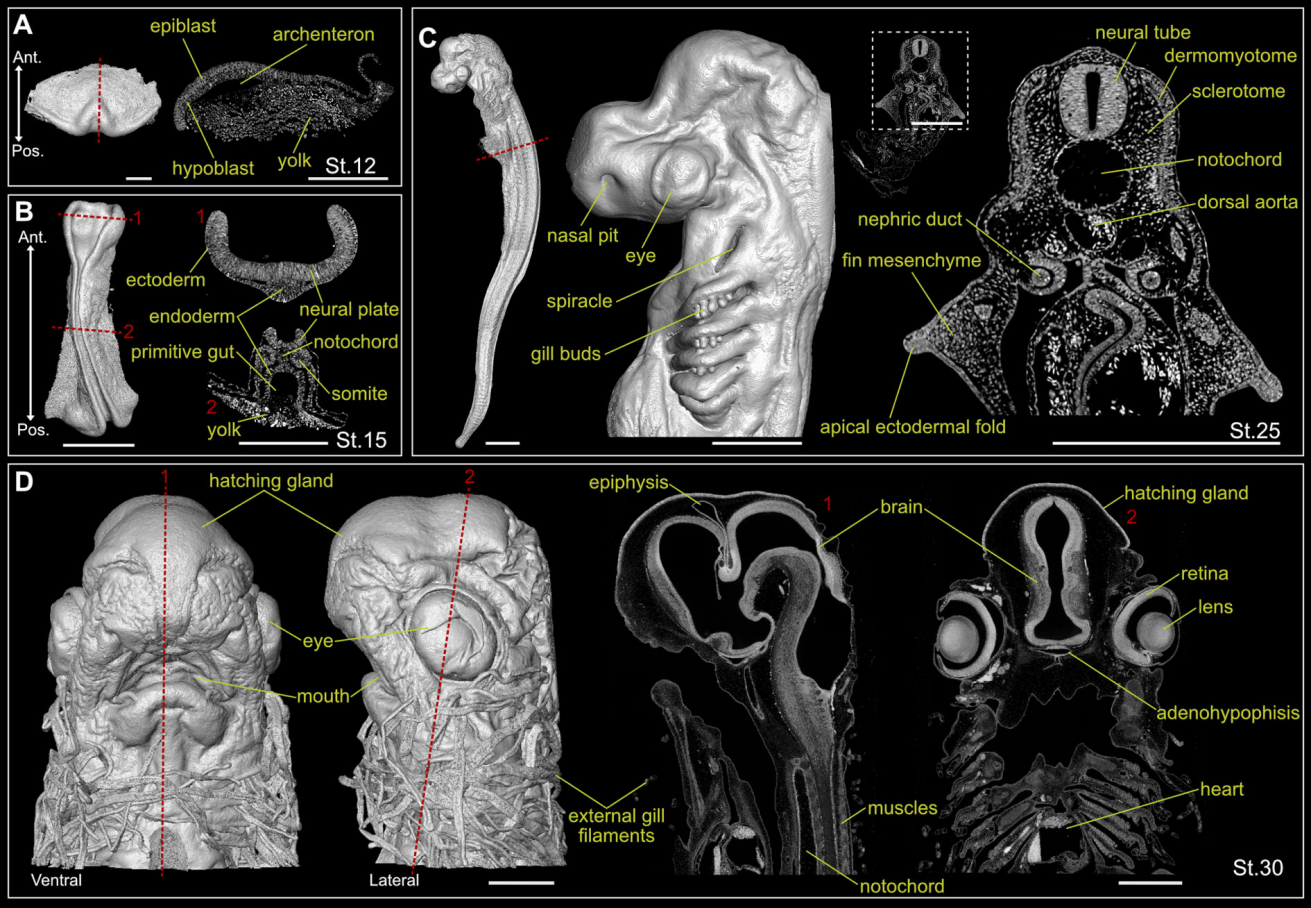
Examples of 3D reconstructions and tomographic slices of small-spotted catshark embryos at different developmental stages. Yellow labeling indicates distinct developing structures. Red dashed lines indicate the anatomical position corresponding to the tomographic slices. (A) St.12 small-spotted catshark embryo. Dorsal view 3D reconstruction (left) and tomographic slice (right). Scale bars: 500 µm. (B) St.15 small-spotted catshark embryo. Dorsal view 3D reconstruction (left) and tomographic slices (right). Tomographic slice 1 through the prospective head region shows the elevation of the neural plate at this stage. Tomographic slice 2 through the trunk region shows the formation of early developmental structures like the somites and primitive gut. Scale bars: 500 µm. (C) St.25 small-spotted catshark embryo. Whole embryo lateral view 3D reconstruction (left) and close-up of the head region (middle). Scale bars: 1 mm. Tomographic slice through the trunk region, where the pectoral fin buds are located, and close-up (right). Scale bars: 500 µm. (D) Ventral and lateral view 3D reconstruction of the head of a St.30 small-spotted catshark embryo (left) and corresponding tomographic slices (right). Scale bars: 500 µm. Ant, anterior; Pos, posterior.

**Figure 5:**
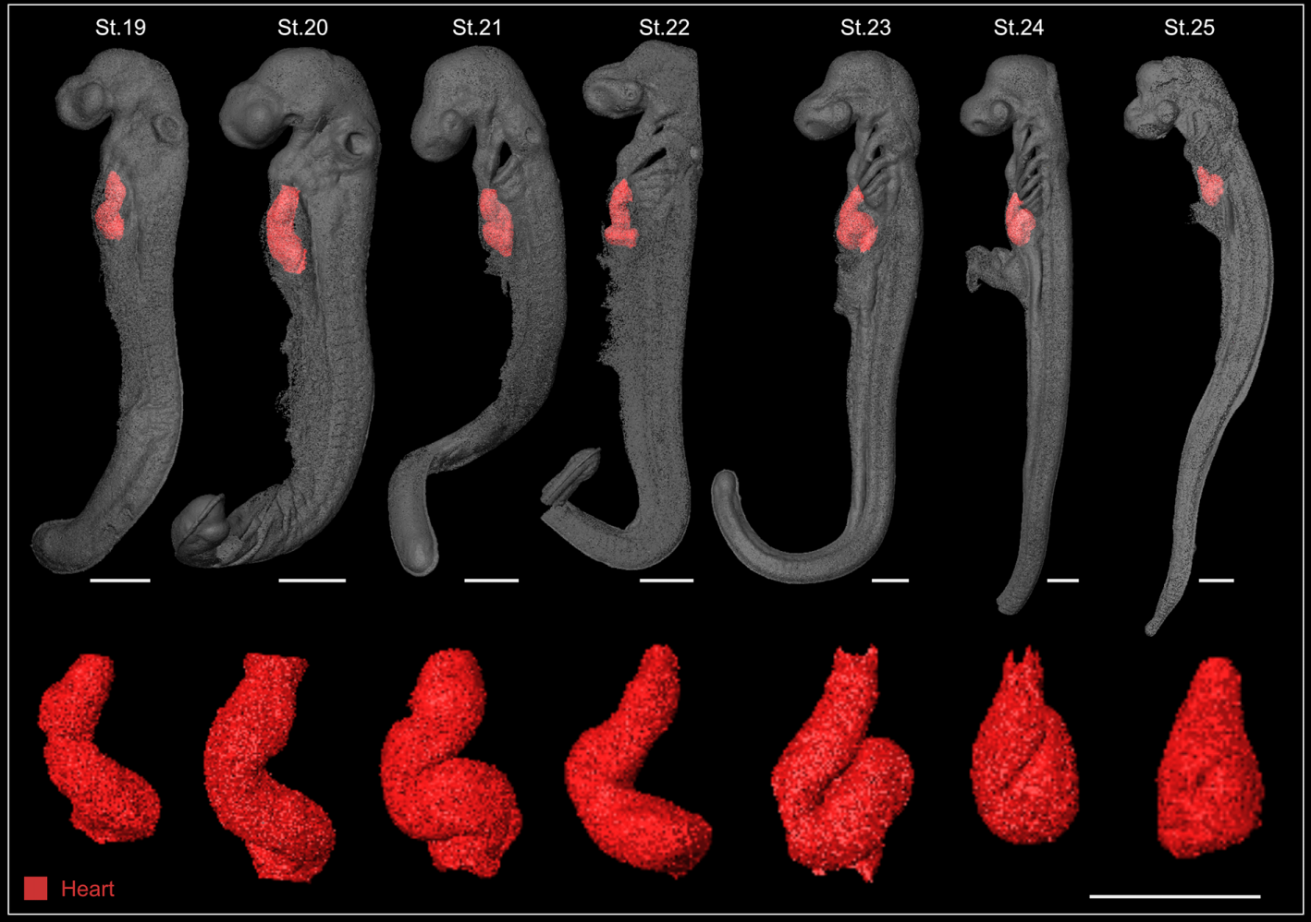
Heart morphogenesis in the small-spotted catshark. 3D reconstructions of whole embryos in lateral view at consecutive developmental stages (top) and corresponding frontal view of the segmented heart (bottom). Imaging of consecutive embryonic stages allowed the reconstruction of heart morphogenesis, from a nearly straight tube at St.19 to the bending into an S-shape at St.21–22, and the acquisition of a compact structure by St.24–25. Scale bars: 500 µm.

Beyond the representative examples shown (Figs 2 and 3), there are numerous opportunities to employ this dataset in a wide spectrum of studies, allowing to analyze the formation of virtually any identifiable embryonic structure: cranial placodes (adenohypophyseal, olfactory, lens, trigeminal, profundal, lateral line, otic, and epibranchial), sensory organs, teeth, skin, skin denticles, head cavities, neural crest cells, facial mesenchyme, cartilage, vertebral column, skull, gills, pharyngeal arches, pharyngeal pouches, somites, muscles, central nervous system (forebrain, midbrain, hindbrain, and spinal cord), peripheral nervous system (cranial ganglia, dorsal root ganglia, nerves), endocrine organs, kidneys, stomach, liver, gut, heart, blood vessels, fins (pectoral, pelvic, dorsal, anal, caudal), reproductive system, and many more (see examples of different structures from representative developmental stages in Fig. 4). This dataset captures the early morphogenetic events that lead to the formation of the general chondrichthyan body plan from the 3 main embryonic germ layers (endoderm, mesoderm, ectoderm), till later stages of organogenesis, when species-specific differences begin to emerge among embryos of distinct chondrichthyan species [87]. The analysis of consecutive developmental stages allows researchers to reconstruct and trace the embryonic origin of the developing anatomical features (Fig. 5), providing key insights into the developmental trajectories and formation of tissues and organs in Chondrichthyes. Moreover, during embryogenesis, tissues and organs arise and grow in a synchronous manner, establishing tight connections and integrating to form a functional organism. Such high-dimensional interactions are challenging to study by conventional 2D and 3D methods and require high-resolution non-destructive imaging techniques. This SRµCT dataset represents a comprehensive resource to resolve tissue architecture and the integration of embryonic structures in 3D space.

Small-spotted catshark embryos at the same developmental stage often exhibit slight morphological differences depending on their precise developmental timing. For instance, early St.26 embryos (~37 days after deposition) have ~85 pairs of somites, while late St.26 embryos (~42 days after deposition) have around 90 pairs of somites [41]. This within-stage variation highlights that embryonic development is a continuous process despite being conventionally subdivided into discrete stages. The presented dataset can serve as a starting point for future studies of within-stage developmental differences since it provides scans of at least 2 independent embryos from St.17 to 29, allowing a more refined characterization of small-spotted catshark development (Supplementary Fig. 3).

Research on Chondrichthyes development is challenging due to the difficulties in obtaining fertilized eggs or embryonic material. The presented SRµCT dataset serves as a valuable and information-rich resource to investigate shark embryonic development and incorporate Chondrichthyes into modern comparative embryology studies, enabling a deeper understanding of the divergence and conservation of the developmental programs shaping vertebrate morphological diversity.

## Additional files

**Supplementary Figure S1:** Brightness and contrast adjustments. Brightness and contrast were adjusted using the Brightness/Contrast function in ImageJ/Fiji 2.9.0 [1] by modifying the display range (minimum and maximum intensity values) following the guidelines from [2]. Intensity values range from 0 to 255 in 8-bit images. Thresholds are shown in blue for minimum and red for maximum values. Pixels with intensities above the maximum threshold are shown in white and pixels below the minimum threshold in black. Images represent a tomographic slice through the head region of a St.23 embryo, accompanied by their respective Brightness/Contrast panel from ImageJ/Fiji. Arrowheads point to the minimum (blue) and maximum (red) intensity values. Scale bar: 500 µm. (A) Progressive clipping of high-intensity values. Excessive clipping of high-intensity values results in overexposed (white) images. (B) Progressive clipping of low-intensity values. Excessive clipping of low-intensity values results in underexposed (black) images. (C) Simultaneous clipping of high- and low-intensity values narrows the intensity range and increases contrast. All images were processed so that the intensity values would remain in an optimal range (C1), meaning that the clipping of low-intensity values removed the gray background, leaving only contrasted tissues, and the clipping of high-intensity values enhanced the brightness of the contrasted structures, without overexposing (C2) or underexposing (C3) them. Each image stack was processed independently depending on its intrinsic intensity range to obtain an optimal clipping range.

**Supplementary Figure S2:** Tissue shrinkage in whole-mount PTA-contrasted small-spotted catshark embryos. 3D reconstructions of representative small-spotted catshark embryos at different developmental stages. Yellow arrowheads indicate the areas where tissue shrinkage is most noticeable, resulting in a wrinkled surface ectoderm. This wrinkling becomes more visible beginning at St.25. The red dashed line indicates the anatomical position of the St.30 tomographic slice. Note that despite the wrinkling of the surface ectoderm, the overall morphology and position of internal structures (e.g., brain) remain preserved. Scale bars: 500 µm.

**Supplementary Figure S3:** Within-stage differences in neural tube closure at St.17. Although the neural tube closes at St.17 in the small-spotted catshark [3] within-stage differences can be observed between early and mid-St.17. At early-St.17 (ScCan_St17_1), the neural folds begin to fuse, but the surface ectoderm has not yet fully fused into a single layer, which results in a slight depression in the midline (yellow arrowheads). By mid-St17 (ScCan_St17_2), the surface ectoderm has fused, and only a small depression can be observed where the anterior neuropore was (yellow arrowhead). Red dashed lines indicate the anatomical position of the corresponding tomographic slices. Scale bars: 500 µm for 3D reconstructions and 250 µm for tomographic slices.

**Supplementary Table S1:** PTA-contrasting time for each small-spotted catshark developmental stage.

**Supplementary Table S2:** Detailed SRµCT scanning parameters.

## List of abbreviations

µCT: micro-computed tomography; µm: micrometer; cm: centimeter; CPU, central processing unit; evo-devo: evolutionary developmental biology; EMPIAR, Electron Microscopy Public Image Archive; FOV: field of view; h: hour; GB: gigabyte; GPU, graphics processing unit; HCR: hybridization chain reaction; min: minute; mm: millimeter; ms: millisecond; PBS: Dulbecco’s phosphate buffered saline; PFA: paraformaldehyde; PTA: phosphotungstic acid; RAM, random-access memory; St: stage; SRµCT: synchrotron radiation micro-computed tomography; TB: terabyte; UV: ultraviolet.

## Supplementary Material

giag054_Supplemental_Files

giag054_Authors_Response_To_Reviewer_Comments_original_submission

giag054_GIGA-D-25-00317_original_submission

giag054_GIGA-D-25-00317_revision_1

giag054_Reviewer_1_Report_original_submission Reviewer 1 -- 9/8/2025

giag054_Reviewer_2_Report_original_submission Reviewer 2 -- 12/3/2025

giag054_Reviewer_2_Report_revision_1 Reviewer 2 -- 3/16/2026

## Data Availability

The SRµCT dataset behind this manuscript is available in the Electron Microscopy Public Image Archive (EMPIAR, accession number: EMPIAR-12984). We provide the raw reconstructed SRµCT data as well as the pre-processed files. Because of the large size of the raw reconstructed files, we recommend using the pre-processed data instead. If using the raw reconstructed files, please note that small misalignments of the vertical tiles may appear due to stitching artifacts and will need to be manually corrected by the operator. All data analysis and segmentations were performed on a workstation equipped with the following hardware components: an NVIDIA Quadro P5000 graphics processing unit (GPU), an Intel Xeon W-2145 central processing unit (CPU), and 64 GB of system memory (RAM). Based on our experience with this configuration, we recommend resampling the files to obtain a final working image stack of less than 2 GB in size, or ideally less than 1 GB when extensive segmentation is expected. However, file size limitations may vary depending on the specification of the workstation used. The dataset is presented as TIFF stacks of the corresponding tomographic slices. Each folder contains the information for one single embryo. The naming of files is as follows: ScCan_stage_replicate_32b.tiff for raw reconstructed files and ScCan_stage_replicate_8b.tiff for pre-processed files. Information about the voxel size for each sample can be found in Supplementary Table 2. For the production, curation, and analysis of this SRµCT dataset, we used ImageJ/Fiji [79] and Avizo3D Pro Software. However, other software options are available to segment, visualize, and analyze this dataset, such as 3D Slicer [88], Amira (Thermo Fisher Scientific), VG Studio MAX (Volume Graphics GmbH, Germany), Mimics (Materialize NV), Dragonfly 3D (Object Research Systems Inc., Comet group), or MeshLab [89]. All additional Supplementary Materials [90] are available in the *GigaScience* repository, GigaDB [91].
