# Supplementary material for: Synchrotron radiation micro-computed tomography of the small-spotted catshark embryonic development (Chondrichthyes: Scyliorhinus canicula)
Source: Gigascience. 2026 May 7;15:giag054. doi: 10.1093/gigascience/giag054 (PMC13240850; doi:10.1093/gigascience/giag054)
Supplement: giag054_Supplemental_Files [file giag054_supplemental_files.zip › SupplementaryFiles_GigaScience_14_02_2026.docx]

**Supplementary Fig. 1. Brightness and contrast adjustments.** Brightness and contrast were adjusted using the Brightness/Contrast function in ImageJ/Fiji 2.9.0 [1] by modifying the display range (minimum and maximum intensity values) following the guidelines from [2]. Intensity values range from 0 to 255 in 8 bits images. Thresholds are shown in blue for minimum and red for maximum values. Pixels with intensities above the maximum threshold are shown in white and pixels below the minimum threshold in black. Images represent a tomographic slice through the head region of a St.23 embryo accompanied by their respective Brightness/Contrast panel from ImageJ/Fiji. Arrowheads point to the minimum (blue) and maximum (red) intensity values. Scale bar: 500µm. **(A)** Progressive clipping of high-intensity values. Excessive clipping of high-intensity values results in overexposed (white) images. **(B)** Progressive clipping of low-intensity values. Excessive clipping of low-intensity values results in underexposed (black) images **(C)** Simultaneous clipping of high- and low-intensity values narrows the intensity range and increases contrast. All images were processed so that the intensity values would remain in an optimal range (C1), meaning that the clipping of low-intensity values removed the gray background leaving only contrasted tissues and the clipping of high-intensity values enhanced the brightness of the contrasted structures, without overexposing (C2) or underexposing (C3) them. Each image stack was processed independently depending on its intrinsic intensity range to obtain an optimal clipping range.

**Supplementary Fig. 2. Tissue shrinkage in whole-mount PTA-contrasted small-spotted catshark embryos.** 3D reconstructions of representative small-spotted catshark embryos at different developmental stages. Yellow arrowheads indicate the areas where tissue shrinkage is most noticeable, resulting in a wrinkled surface ectoderm. This wrinkling becomes more visible beginning at St.25. The red dashed line indicates the anatomical position of the St.30 tomographic slice. Note that despite the wrinkling of the surface ectoderm, the overall morphology and position of internal structures (e.g., brain) remain preserved. Scale bars: 500µm.

**Supplementary Fig. 3. Within-stage differences in neural tube closure at St.17.** Although the neural tube closes at St.17 in the small-spotted catshark [3] within-stage differences can be observed between early and mid-St.17. At early-St.17 (ScCan_St17_1) the neural folds begin to fuse but the surface ectoderm has not yet fully fused into a single layer, which results in a slight depression in the midline (yellow arrowheads). By mid-St17 (ScCan_St17_2), the surface ectoderm has fused and only a small depression can be observed where the anterior neuropore was (yellow arrowhead). Red dashed lines indicate the anatomical position of the corresponding tomographic slices. Scale bars: 500µm for 3D reconstructions and 250µm for tomographic slices.

**Supplementary Table 1. PTA-contrasting time for each small-spotted catshark developmental stage.**

| **Developmental stage** | **PTA-contrasting time** |
| --- | --- |
| St.12-22 | 1 week |
| St.23-28 | 2 weeks |
| St.29-31 | 3 weeks |

**Supplementary Table 2. Detailed SRµCT scanning parameters.**

| **Sample ID** | **Embryonic stage** | **Somite number** | **Embryo length (mm)** | **Projections** | **FOV (mm)** | **Exposure time (ms)** | **Voxel size (µm)** | **Scan time** | **Multiscan (tiles)** |
| --- | --- | --- | --- | --- | --- | --- | --- | --- | --- |
| ScCan_St12_1 | St.12 | 0 | 0.60 | 3001 | 3.29 x 2.47 | 280 | 1.28 | 16.4min | 1 |
| ScCan_St13_1 | St.13 | 0 | 0.64 | 3001 | 3.29 x 2.47 | 280 | 1.28 | 16.4min | 1 |
| ScCan_St14_1 | St.14 | 1 | 1.31 | 3001 | 3.29 x 2.47 | 280 | 1.28 | 16.4min | 1 |
| ScCan_St14_2 | Late St.14 | 4 | 1.72 | 3001 | 3.29 x 2.47 | 280 | 1.28 | 16.4min | 1 |
| ScCan_St15_1 | St.15 | 11 | 2.17 | 2501 | 3.29 x 2.47 | 270 | 1.32 | 13.4min | 1 |
| ScCan_St16_1 | St.16 | 15 | 1.49 | 2501 | 3.29 x 2.47 | 270 | 1.32 | 13.4min | 1 |
| ScCan_St17_1 | Early St.17 | 16 | 2.53 | 3001 | 3.29 x 2.47 | 280 | 1.28 | 16.4min | 1 |
| ScCan_St17_2 | St.17 | 19 | 2.72 | 3001 | 3.29 x 2.47 | 280 | 1.28 | 32.8min | 2 |
| ScCan_St18_1 | St.18 | 27 | 3.99 | 3001 | 3.29 x 2.47 | 280 | 1.28 | 32.8min | 2 |
| ScCan_St19_1 | Early St.19 | 30 | 3.69 | 3001 | 3.29 x 2.47 | 280 | 1.28 | 16.4min | 1 |
| ScCan_St19_2 | St.19 | 37 | 4.84 | 3001 | 3.29 x 2.47 | 280 | 1.28 | 32.8min | 2 |
| ScCan_St19_3 | St.19 | 39 | 5.22 | 3001 | 3.29 x 2.47 | 280 | 1.28 | 49.2min | 3 |
| ScCan_St20_1 | St.20 | 43 | 5.95 | 3001 | 3.29 x 2.47 | 280 | 1.28 | 32.8min | 2 |
| ScCan_St20_2 | St.20 | 46 | 5.92 | 3001 | 3.29 x 2.47 | 280 | 1.28 | 32.8min | 2 |
| ScCan_St21_1 | Early St.21 | 51 | 6.55 | 3001 | 3.29 x 2.47 | 280 | 1.28 | 32.8min | 2 |
| ScCan_St21_2 | St.21 | 52 | 7.14 | 3001 | 3.29 x 2.47 | 280 | 1.28 | 49.2min | 3 |
| ScCan_St21_3 | St.21 | 55 | 8.06 | 3001 | 3.29 x 2.47 | 280 | 1.28 | 49.2min | 3 |
| ScCan_St22_1 | St.22 | 55 | 8.07 | 3001 | 3.29 x 2.47 | 280 | 1.28 | 49.2min | 3 |
| ScCan_St22_2 | Early St.22 | 57 | 7.33 | 3001 | 3.29 x 2.47 | 280 | 1.28 | 49.2min | 3 |
| ScCan_St22_3 | St.22 | 59 | 8.94 | 3001 | 3.29 x 2.47 | 280 | 1.28 | 65.6min | 4 |
| ScCan_St23_1 | St.23 | 61 | 9.52 | 3001 | 3.29 x 2.47 | 280 | 1.28 | 49.2min | 3 |
| ScCan_St23_2 | St.23 | 69 | 10.3 | 3001 | 3.29 x 2.47 | 280 | 1.28 | 65.6min | 4 |
| ScCan_St24_1 | St.24 | 69 | 9.01 | 3001 | 3.29 x 2.47 | 280 | 1.28 | 65.6min | 4 |
| ScCan_St24_2* | Late St.24 | 61 | 9.93 | 3001 | 3.29 x 2.47 | 280 | 1.28 | 98.4min | 6 |
| ScCan_St25_1 | St.25 | 82 | 14.5 | 3001 | 3.29 x 2.47 | 280 | 1.28 | 98.4min | 6 |
| ScCan_St25_2 | St.25 | 85 | 12.0 | 3001 | 3.29 x 2.47 | 280 | 1.28 | 98.4min | 6 |
| ScCan_St26_1* | St.26 | - | 15.0 | 3001 | 6.57 x 2.70 | 80 | 2.57 | 32.9min | 7 |
| ScCan_St26_2* | St.26 | - | 15.2 | 3001 | 6.57 x 2.70 | 80 | 2.57 | 32.9min | 7 |
| ScCan_St27_1 | St.27 | - | 18.0 | 3001 | 6.57 x 2.70 | 80 | 2.57 | 37.6min | 8 |
| ScCan_St27_2 | St.27 | - | 19.1 | 3001 | 6.57 x 2.70 | 80 | 2.57 | 37.6min | 8 |
| ScCan_St28_1 | St.28 | - | 21.3 | 3001 | 6.57 x 2.70 | 80 | 2.57 | 42.3min | 9 |
| ScCan_St28_2 | St.28 | - | 19.2 | 3001 | 6.57 x 2.70 | 80 | 2.57 | 37.6min | 8 |
| ScCan_St29_1 | St.29 | - | 26.0 | 3001 | 6.57 x 2.70 | 80 | 2.57 | 51.7min | 11 |
| ScCan_St29_2 | St.29 | - | 26.3 | 3001 | 6.57 x 2.70 | 80 | 2.57 | 51.7min | 11 |
| ScCan_St30_1 | St.30 | - | 31.3 | 3001 | 6.57 x 2.70 | 80 | 2.57 | 65.8min | 14 |
| ScCan_St31_1 | St.31 | - | 35.5 | 3001 | 6.57 x 2.70 | 80 | 2.57 | 75.2min | 16 |

Additionally, all samples were scanned with a photon energy of 20keV and a sample-to-detector distance of 80mm. Embryo length was measured from anterior to posterior ends. Somite counts are provided until St.25, after which anterior somites begin to differentiate and are no longer readily distinguishable. *These samples got damaged during preparation and the last portion of their tails is missing.

**Supplementary References**

1. Schindelin J, Arganda-Carreras I, Frise E, Kaynig V, Longair M, Pietzsch T, et al.. Fiji: an open-source platform for biological-image analysis. *Nat Methods*. Nature Publishing Group; 2012; doi: 10.1038/nmeth.2019.

2. Schmied C, Jambor HK. Effective image visualization for publications – a workflow using open access tools and concepts. F1000Research;

3. Ballard WW, Mellinger J, Lechenault H. A series of normal stages for development of Scyliorhinus canicula, the lesser spotted dogfish (Chondrichthyes: Scyliorhinidae). *Journal of Experimental Zoology*. 1993; doi: 10.1002/jez.1402670309.
