# Supplementary figures and images for: Synchrotron radiation micro-computed tomography of the small-spotted catshark embryonic development (Chondrichthyes: Scyliorhinus canicula)
Source: Gigascience. 2026 May 7;15:giag054. doi: 10.1093/gigascience/giag054 (PMC13240850; doi:10.1093/gigascience/giag054)

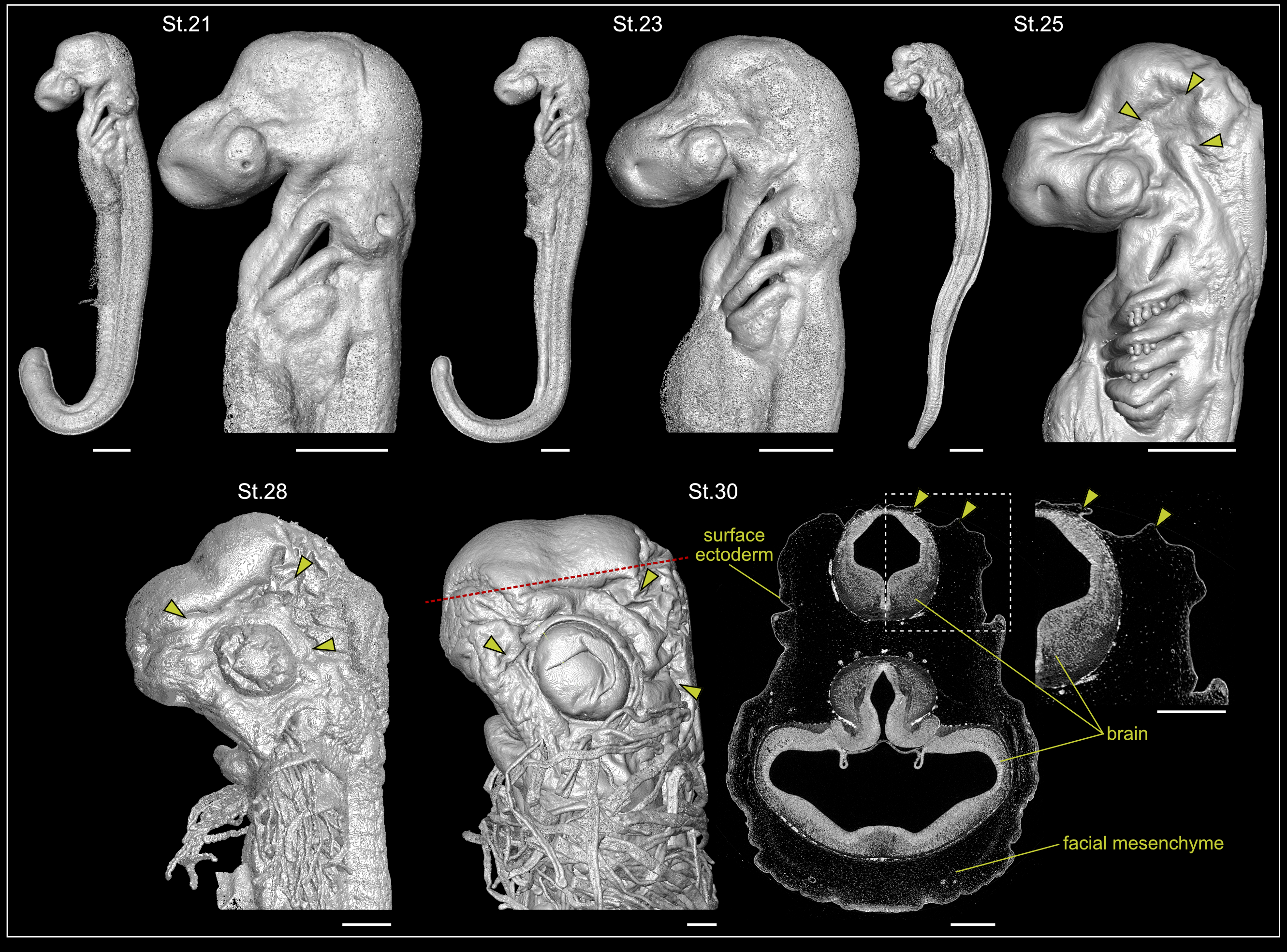

Supplement: giag054_Supplemental_Files [file giag054_supplemental_files.zip › Supplementary_Figure_1.tiff]

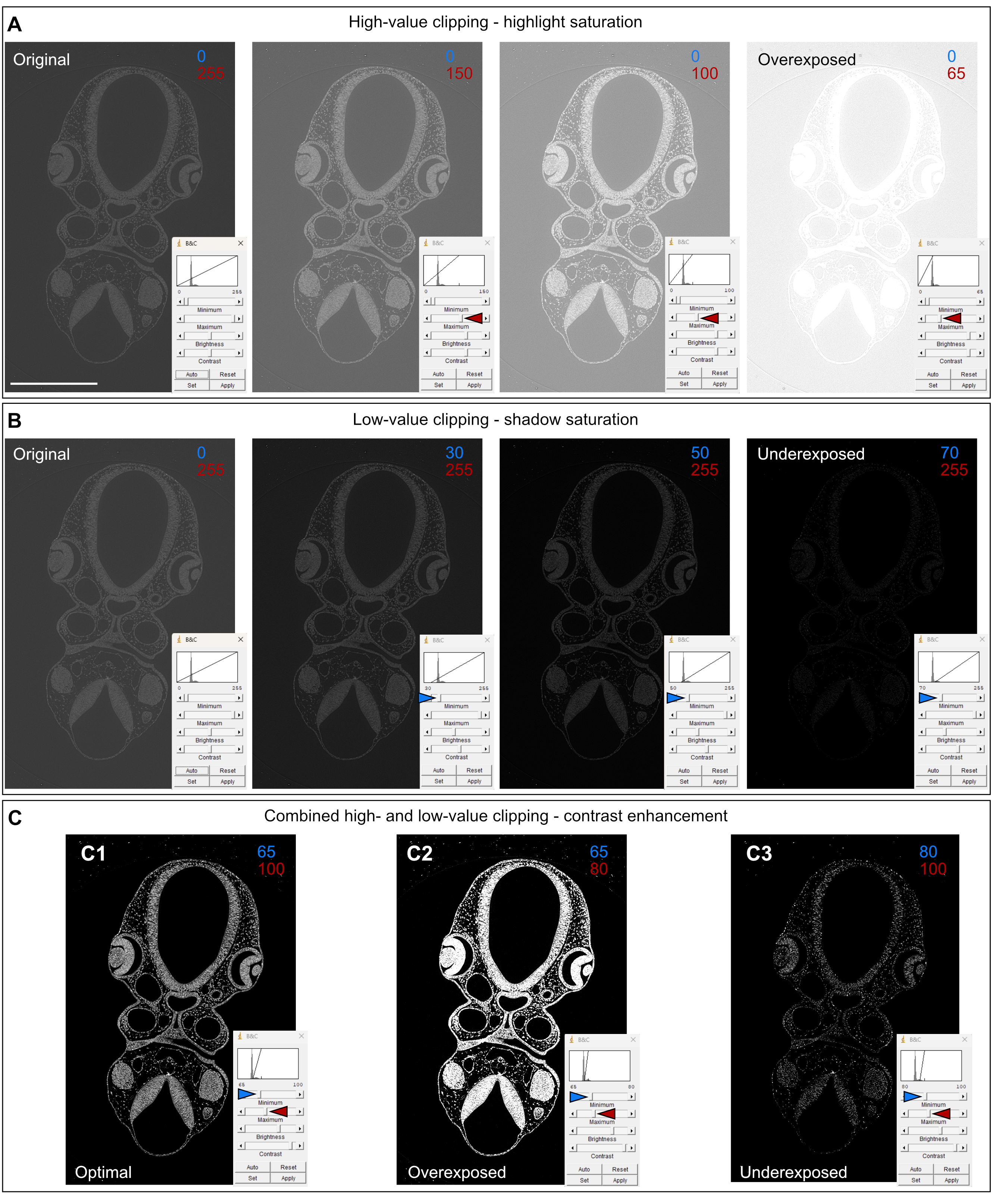

Supplement: giag054_Supplemental_Files [file giag054_supplemental_files.zip › Supplementary_Figure_2.tiff]

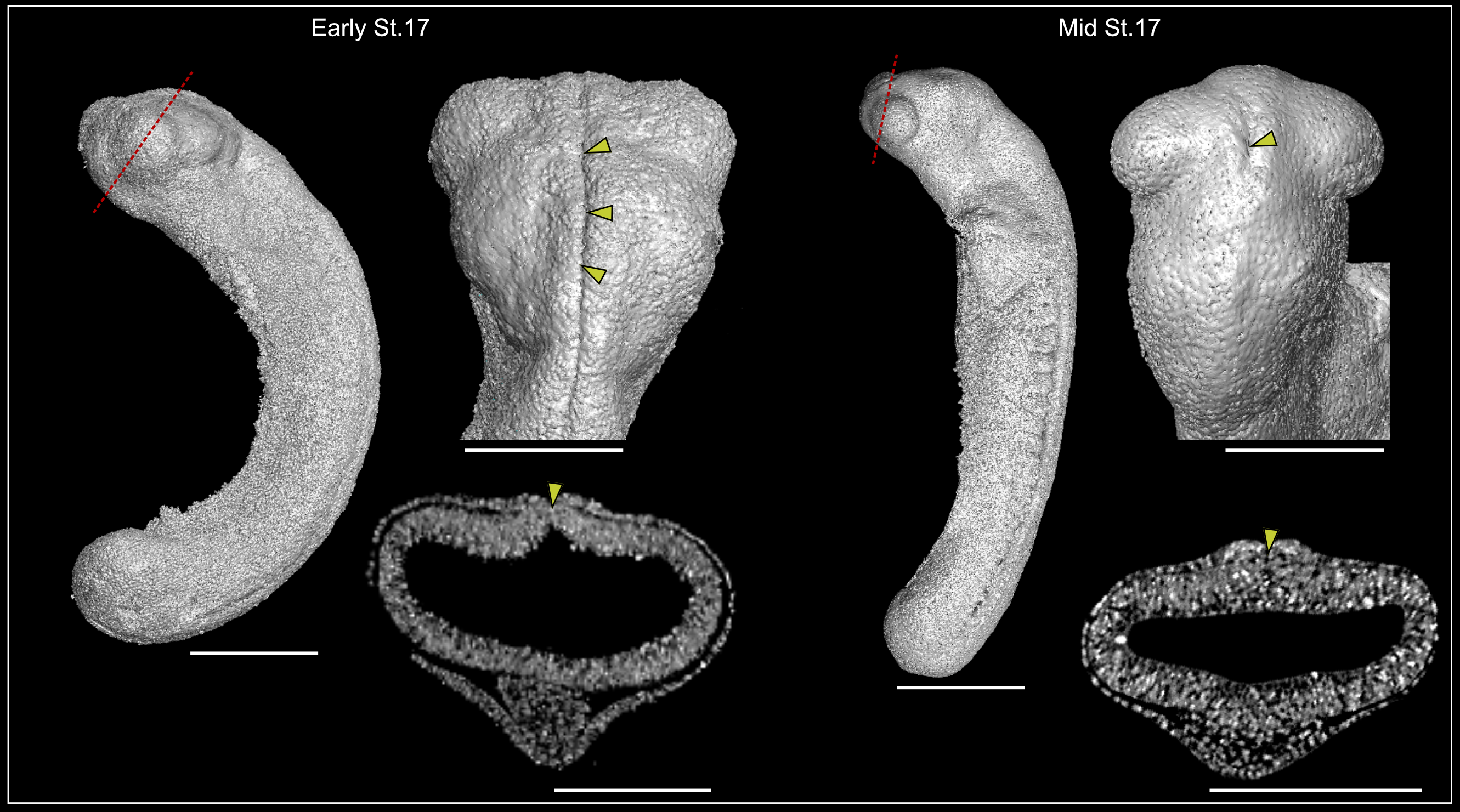

Supplement: giag054_Supplemental_Files [file giag054_supplemental_files.zip › Supplementary_Figure_3.tiff]
