# Supplementary material for: Synchrotron radiation micro-computed tomography of the small-spotted catshark embryonic development (Chondrichthyes: Scyliorhinus canicula)
Source: Gigascience. 2026 May 7;15:giag054. doi: 10.1093/gigascience/giag054 (PMC13240850; doi:10.1093/gigascience/giag054)
Supplement: giag054_GIGA-D-25-00317_original_submission [file giag054_giga-d-25-00317_original_submission.pdf]

GigaScience

Synchrotron radiation micro-computed tomography of the small-spotted catshark embryonic development (Chondrichthyes: Scyliorhinus canicula)

--Manuscript Draft--

**Manuscript Number:**	GIGA-D-25-00317
**Full Title:**	Synchrotron radiation micro-computed tomography of the small-spotted catshark embryonic development (Chondrichthyes: Scyliorhinus canicula)
**Article Type:**	Data Note
**Funding Information:**	
**Abstract:**	Background<br><br>Sharks occupy a key position on vertebrate phylogeny, making them essential for understanding the early origins of jawed vertebrates (gnathostomes) and the functional adaptation of vertebrate traits like jaws or complex sensory systems. As such, sharks are important model organisms in evolutionary developmental biology (evo-devo), but sparse data and limited availability of samples hinder their inclusion in contemporary evo-devo research. The knowledge of their distinctive morphology will not only shed light on the anatomical architecture and physiology of basal living gnathostomes but also reveal the evolutionary divergence of developmental processes that establish the foundational vertebrate blueprint.<br><br>Findings<br><br>We performed synchrotron radiation micro-computed tomography (SRµCT) scanning of the small-spotted catshark (Scyliorhinus canicula) embryonic development, spanning from gastrulation (stage 12) to late-organogenesis (stage 31). We obtained 36 whole-embryo scans that encompass the formation of key embryonic structures, such as sensory organs, teeth, muscles and skeletal elements. Cellular resolution was achieved by enhanced tissue contrasting with phosphotunstic acid. The achieved resolution allows for segmentation of all tissue types and both internal and external structures.<br><br>Conclusions<br><br>We present a comprehensive dataset of 4D high-resolution SRµCT of the small-spotted catshark embryonic development. The dataset spans consecutive embryonic stages, allowing the reconstruction and morphometric analyses of tissues, organs, and structures, along with the tracking of their development. The deposited data are publicly available, and provide a valuable resource for comparative research, additionally allowing the identification of conserved and derived developmental processes and features and understanding the evolution of vertebrates.
**Corresponding Author:**	Marketa Kaucka, PhD<br>Max Planck Institute for Evolutionary Biology: Max-Planck-Institut fur Evolutionsbiologie<br>Ploen, GERMANY
**Corresponding Author Secondary Information:**	
**Corresponding Author's Institution:**	Max Planck Institute for Evolutionary Biology: Max-Planck-Institut fur Evolutionsbiologie
**Corresponding Author's Secondary Institution:**	
**First Author:**	Elio Escamilla-Vega
**First Author Secondary Information:**	
**Order of Authors:**	Elio Escamilla-Vega
	Ann-Katrin Koch

Powered by Editorial Manager® and ProduXion Manager® from Aries Systems Corporation

	Louk W. G. Seton
	Andrea P. Murillo-Rincón
	Stella Kyomen
	Jörg U. Hammel
	Timo Moritz
	Marketa Kaucka
**Order of Authors Secondary Information:**	
**Additional Information:**	
**Question**	**Response**
Are you submitting this manuscript to a special series or article collection?	No
**Experimental design and statistics**<br><br>Full details of the experimental design and statistical methods used should be given in the Methods section, as detailed in our Minimum Standards Reporting Checklist. Information essential to interpreting the data presented should be made available in the figure legends.<br><br>Have you included all the information requested in your manuscript?	No
If not, please give reasons for any omissions below.<br><br>as follow-up to "**Experimental design and statistics**<br><br>Full details of the experimental design and statistical methods used should be given in the Methods section, as detailed in our Minimum Standards Reporting Checklist. Information essential to interpreting the data presented should be made available in the figure legends.<br><br>Have you included all the information requested in your manuscript?<br><br>"	No statistical analyses were performed in this study.
**Resources**	Yes

A description of all resources used, including antibodies, cell lines, animals and software tools, with enough information to allow them to be uniquely identified, should be included in the Methods section. Authors are strongly encouraged to cite Research Resource Identifiers (RRIDs) for antibodies, model organisms and tools, where possible.<br><br>Have you included the information requested as detailed in our Minimum Standards Reporting Checklist?	
**Availability of data and materials**<br><br>All datasets and code on which the conclusions of the paper rely must be either included in your submission or deposited in publicly available repositories (where available and ethically appropriate), referencing such data using a unique identifier in the references and in the "Availability of Data and Materials" section of your manuscript.<br><br>Have you have met the above requirement as detailed in our Minimum Standards Reporting Checklist?	Yes
GigaScience has policies and guidelines in place for the use of generative AI-writing tools such as ChatGPT. If you have used such writing tools to assist with writing the manuscript this must be declared and cited in the text. Authors should not list AI-writing tools and other AI-assisted technologies as an author or co-author and should acknowledge that they are fully responsible for text generated or refined by AI-writing tools.<p><br><br>A summary of use (particularly in the introduction or among methods) needs to be included at the end of the paper, and the outputs should also be included as a supplementary file hosted in GigaDB or other open repositories. Please <a href=https://academic.oup.com/gigascienc	No

e/pages/editorial_policies_and_reporting_standards target="_new" > read our guidelines for more information. <p><br><br>By submitting to GigaScience, you are aware of the journal's AI-writing tools policy, and if you have declared use of such tools below, you have acknowledged this where appropriate in your manuscript and have made a summary of use and outputs available. <p><br><b>AI-assisted writing tools have been used in the preparation of this manuscript?	

Manuscript file
Click here to access/download;Manuscript;MainText_GigaScience_Final.docx

Synchrotron radiation micro-computed tomography of the small-spotted catshark embryonic development (Chondrichthyes: *Scyliorhinus canicula*)

Authors

Elio Escamilla-Vega[1], Ann-Katrin Koch[2,3], Louk W. G. Seton[1], Andrea P. Murillo-Rincón[1], Stella Kyomen[1], Jörg U. Hammel[4], Timo Moritz[2,5], Markéta Kaucká[1*]

[1]Max Planck Institute for Evolutionary Biology, August-Thienemann-Str. 2, 24306 Plön, Germany

[2]Ocean Museum Germany, Katharinenberg 14–20, 18439 Stralsund, Germany

[3]Institute of Biosciences, University of Rostock, Albert-Einstein-Str. 3, 18059 Rostock, Germany

[4]Institute of Materials Physics, Helmholtz-Zentrum Hereon, Max-Planck-Str. 1, 21502 Geesthacht, Germany

[5]Leibniz Institute for the Analysis of Biodiversity Change, Martin-Luther-King-Platz 3, D-20146 Hamburg, Germany

e-mail address of all authors:

Elio Escamilla-Vega: escamilla@evolbio.mpg.de

Ann-Katrin Koch: Ann-Katrin.Koch@meeresmuseum.de

Louk W.G. Seton: seton@evolbio.mpg.de

Andrea P. Murillo-Rincón: amurillo@evolbio.mpg.de

Stella Kyomen: kyomen@evolbio.mpg.de

Jörg U. Hammel: joerg.hammel@hereon.de

Timo Moritz: T.Moritz@leibniz-lib.de

Markéta Kaucká: kaucka@evolbio.mpg.de

***Corresponding author**

Markéta Kaucká: kaucka@evolbio.mpg.de

Abstract

Background. Sharks occupy a key position on vertebrate phylogeny, making them essential for understanding the early origins of jawed vertebrates (gnathostomes) and the functional adaptation of vertebrate traits like jaws or complex sensory systems. As such, sharks are important model organisms in evolutionary developmental biology (evo-devo), but sparse data and limited availability of samples hinder their inclusion in contemporary evo-devo research. The knowledge of their distinctive morphology will not only shed light on the anatomical architecture and physiology of basal living gnathostomes but also reveal the evolutionary divergence of developmental processes that establish the foundational vertebrate blueprint.

Findings. We performed synchrotron radiation micro-computed tomography (SRμCT) scanning of the small-spotted catshark (*Scyliorhinus canicula*) embryonic development, spanning from gastrulation (stage 12) to late-organogenesis (stage 31). We obtained 36 whole-embryo scans that encompass the formation of key embryonic structures, such as sensory organs, teeth, muscles and skeletal elements. Cellular resolution was achieved by enhanced tissue contrasting with phosphotunstic acid. The achieved resolution allows for segmentation of all tissue types and both internal and external structures.

Conclusions. We present a comprehensive dataset of 4D high-resolution SRμCT of the small-spotted catshark embryonic development. The dataset spans consecutive embryonic stages, allowing the reconstruction and morphometric analyses of tissues, organs, and structures, along with the tracking of their development. The deposited data are publicly available, and provide a valuable resource for

comparative research, additionally allowing the identification of conserved and derived developmental processes and features and understanding the evolution of vertebrates.

Issue section: Data Note

Keywords: synchrotron radiation micro-computed tomography, X-rays, tissue contrast, cartilaginous fish, Chondrichthyes, small-spotted catshark, *Scyliorhinus canicula*, shark ontogeny, evo-devo.

Background

Vertebrate development represents a series of tightly regulated steps that orchestrate the formation of all body structures and their functional integration. While many fundamental processes, such as axis formation, segmentation, and organ primordia patterning, are shared across taxa, there are also significant differences, resulting in the remarkable morphological diversity of vertebrates [1–4]. The differences in embryogenesis reflect both phylogenetic divergence and species-specific adaptations, such as ecological and reproductive pressures [5–7]. Comparative embryology across a broad range of vertebrates is therefore essential for disentangling ancestral traits from lineage-specific innovations and for understanding the evolution of developmental processes that generate morphological diversity.

In the past century, a limited repertoire of model vertebrate organisms has been used for comparative embryology research. These studies provided a solid foundation for our understanding of vertebrate development, and identified events, structures and embryonic stages where the evolutionary divergence of developmental programs arises. Mouse, chicken, clawed frogs, and zebrafish are among the most commonly used species in developmental biology [8,9]. More recently, the taxon sampling has expanded with the introduction of emerging experimental organisms like bats [10,11], ostriches [12,13], lizards

[14,15] and non-teleost fishes [16,17], which has enabled comparative embryology to provide valuable insights into the mechanisms underlying the evolution of vertebrate developmental programs. However, all these species belong to the same superclass of bony vertebrates (Osteichthyes), thus not covering the entire vertebrate subphylum. Despite notable progress in breeding and maintaining jawless vertebrates (cyclostomes – lamprey and hagfish) [18], the vast majority of vertebrate evo-devo research remains mostly restricted to representatives of Osteichthyes. Consequently, a key phylogenetic node, cartilaginous fishes (Chondrichthyes), remains largely unexplored.

Chondrichthyes are the sister group of Osteichthyes and represent one of the two lineages of living jawed vertebrates (gnathostomes). This morphologically diverse group of animals, which diverged from a common ancestor about 420 million years ago, is divided into two classes: Elasmobranchii (sharks, rays, skates and sawfish) and Holocephali (chimaeras) [19]. Their unique features and phylogenetic position render Chondrichthyes a valuable taxon in vertebrate comparative evo-devo research [20–23]. However, a broader inclusion of Chondrichthyes in contemporary embryological research remains limited due to several inherent challenges. The difficulty in obtaining sufficient numbers of embryos resulting from reduced mating in captivity [24–26], restricted habitats (primarily chimaeras), seasonal breeding with low fecundity [27], reproductive modes that often require the sacrifice of adult females [28,29], high extinction risks [30], long generation times (approximately 175 days in the small-spotted catshark) [31], and the challenges associated with maintaining these species in aquarium settings, hinder their establishment as laboratory model system. Nevertheless, despite these limitations, two oviparous species have emerged as promising model organisms in evo-devo research: the little skate (*Leucoraja erinacea*) and the small-spotted catshark (*Scyliorhinus canicula*) [26]. The small-spotted catshark is an abundant, non-endangered species commonly kept in aquaria, capable of mating in captivity and laying eggs throughout most of the year. Moreover, this shark species is well-suited for modern genomic approaches, supported by a chromosomal-level genome assembly and numerous transcriptomic resources [32–35].

Non-destructive three-dimensional imaging techniques, such as confocal laser scanning microscopy by immunofluorescence and *in situ* hybridization, have been used to visualize and follow the development of specific anatomical structures [36–39]. However, applying these techniques to non-model organisms remains challenges due to the limited availability of specific and effective antibodies for immunostaining, as well as the lack of high-quality genome assemblies, which hinder the design of *in situ* hybridization probes. Additionally, although confocal microscopy remains as one of the most popular and widespread 3D imaging techniques, it is limited by the need for fluorescent labelling of the structures of interest, and insufficient light penetration becomes problematic in larger samples. To overcome these limitations and image larger samples, we explored the use of X-ray-based methods, which allow imaging of thick samples at the centimeter scale.

Micro-computed tomography (µCT) is a frequently used approach in developmental biology [40–42]. Samples are irradiated with X-rays from multiple angles, obtaining 2D projections which are later computationally reconstructed into 3D models. Even though µCT provides an excellent way to obtain high-resolution 3D data, it also has two main limitations. The first one is its inability to effectively differentiate soft tissues due to insufficient differences in their X-ray attenuation coefficients. As a result, only dense structures like bones, teeth and scales are readily visible in the reconstructed tomographic images [43,44]. This limitation can be overcome by using contrasting agents, which are differentially absorbed by soft tissues, allowing their visualization in the final images [45]. Various contrast-enhancing agents have been tested, including iodine [41,46–48], ruthenium red [49], and phosphotungstic acid (PTA) [40,50,51]. The second, unavoidable limitation arises from the physical properties of conventional laboratory-based µCT systems. These conventional scanners are prone to beam hardening artifacts, low signal-to-noise ratios, and resolution constraints, particularly when imaging smaller samples, which can make fine structures indistinguishable in the final images [52,53]. In synchrotron facilities, the high-energy nearly-parallel monochromatic X-ray beam makes it possible to obtain high spatial resolution (i.e.

micron scale) images of macroscopic samples (centimeter range) with improved quality and reduced imaging times [54,55].

Here, we took advantage of synchrotron radiation micro-computed tomography (SRµCT) combined with PTA-contrasting to generate a high-resolution 4D (space and time) atlas of the small-spotted catshark development. SRµCT is particularly valuable for studying shark embryos, which are often large and difficult to image using optical techniques. This strategy allowed the manual segmentation of a wide range of embryonic structures, and in turn, the reconstruction of the developmental progression that shape the shark body plan and anatomical features. We provide the raw reconstructed tomographic slices together with the pre-processed files. The provided dataset with superior quality and excellent contrast will benefit researchers interested in Chondrichthyes embryology and comparative evo-devo studies, addressing a wide spectrum of research questions, without requiring access to new specimens or museum-preserved samples.

Methods

Small-spotted catshark sample preparation

Fertilized small-spotted catshark eggs were obtained from Ozeaneum (Stralsund, Germany) and Sea Life Berlin (Berlin, Germany) (Fig. 1A), opened using sharp dissecting scissors and the whole content of the eggs were placed in glass Petri dishes with oxygenated sea water (Fig. 1B). The embryos were then carefully separated from the yolk under a stereomicroscope, staged according to Ballard [31], euthanized by a tricaine (Ethyl-3-aminobenzoat-methansulfonat, Merck, E10521) overdose, and fixed in freshly prepared 4% paraformaldehyde (PFA) in 1X Dulbecco's Phosphate Buffered Saline (PBS – Sigma D5652) for 24h at 4°C with gentle rotation. Following fixation, embryos were washed with PBS and subsequently dehydrated in increasing ethanol (Fisher Chemical E/0650DF/17) steps (30%, 50%, 75% and 90%) for 24h each on slow rotation. The slow rotation together with the long ethanol steps ensures an evenly

dehydration while minimizing tissue shrinking and preserving of the morphological features. Embryos were then incubated in 1.5% PTA (Sigma P4006) in 90% methanol (Fisher Chemical M/4056/17) to enhance the contrast and visualize soft tissues [40,50,51]. The PTA contrasting solution was replaced twice a week. The staining times for each developmental stage are detailed in Supplementary Table 1. Once the embryos were saturated with contrasting solution, they were washed twice in 100% ethanol for 24h to remove any PTA excess and stored in 100% ethanol till scanning.

SRµCT image acquisition

PTA-contrasted small-spotted catshark embryos were placed inside bottom-sealed plastic pipette tips filled with 100% ethanol (Fig. 1C). The pipette tips were bottom-sealed using UV-light sensitive resin and their volume was chosen with respect to the size of the embryo (Fisher Scientific - 10µL 0030073371, 200µL 0030073436, 1000µL 0030000927), with larger embryos placed in larger tips and vice versa. The conical shape of the pipette tips keeps the embryos in place and prevents movements during scanning. Once the embryos were fixed inside the pipette tips, they were carefully aligned as straight as possible using fine forceps in the center to avoid scanning artifacts. Subsequently, the tips were top-sealed using a hot glue gun to avoid drying of the samples during the scans, and glued to a standardized sample holder that fits in the rotation stage.

Attenuation-contrast SRµCT measurements were acquired at the Imaging Beamline P05 of the storage ring PETRA III (Deutsches Elektronen Synchrotron–DESY, Hamburg, Germany) operated by the Helmholtz-Zentrum Geesthacht [56] (Fig. 1D). All embryos were imaged with a photon energy of 20keV and a sample-to-detector distance of 80mm. 3001 projections equally spaced between 0 and π were obtained for each tomographic scan. To optimize scanning time while obtaining the highest possible resolution for each sample, embryos were divided in two groups depending on their size and imaged with a different field of view (FOV) and exposure time. Embryos ranging from St.12-25 were imaged with a

FOV of 3.29mm x 2.47mm, with an exposure time of 280ms. Embryos ranging from St.26-31 were imaged with a FOV of 6.57mm x 2.70mm, with an exposure time of 80ms. Multiscan vertical tiling was applied depending on embryo length to cover the whole specimen, with 16 tiles for the largest embryo (St.31). The scanning time for one tile was 16.4min and 4.7min for each FOV, respectively. Interestingly, the interaction between the X-ray radiation and the PTA contrasting agent causes the samples to turn temporarily blue after scanning (Fig. 1E). Detailed information regarding the SRμCT scanning parameters per sample can be found in Supplementary Table 2.

Image reconstruction and pre-processing

Tomographic reconstruction of the 2D projections into 3D volumes was performed with a two-fold binning based on a custom reconstruction pipeline implemented in MATLAB and the Astra Toolbox [57–59] (Fig. 1F). The resulting reconstructed 3D images were saved as image stacks with an effective isotropic voxel size of 1.28μm and 2.57μm for each FOV, respectively. For samples requiring multiple vertical scans, the resulting tiles were stitched together using the same custom-designed algorithm. Manual adjustment of the stitched tiles was required to correct for minor misalignment artifacts observed in some samples. The final reconstructed and stitched files ranged between 46.8GB and 381GB. In total, we generated 5.04TB of 3D data for further analysis.

Due to the large size of the reconstructed files, additional pre-processing was required before image analysis or manual segmentations could be performed. The 2D reconstructed stacks were first loaded into ImageJ/Fiji 2.9.0 [60] and cropped to retain only the embryos while removing the surrounding empty space. Afterwards, the brightness and contrast were adjusted to refine the range of color values and enhance visualization of the embryonic structures. Finally, images were converted from 32-bit into 8-bit format (Fig. 1F). By performing this series of transformations, a reconstructed stack of 46.8GB reduced its size to approximately 1.3GB. While the pre-processing steps were typically sufficient to reduce the

size of most files, larger samples (St.25-31) required additional resampling to further reduce loading times and ensure efficient segmentations. Although resampling decreased the number of pixels and, thus, reduced image quality, the obtained processed images remained of high quality and resolution.

Manual segmentation of embryonic structures

Based on the pre-processed reconstructed SRµCT images, the different embryonic structures of the developing small-spotted catshark embryos were manually segmented using the interpolation and wrap functions from Avizo3D Pro Software (ThermoFisher Scientific, Konrad-Zuse-Zentrum, Berlin, Germany) (Fig. 2). With the interpolation function, the experienced operator manually segmented every third slice on the same orthogonal projection and the rest was automatically calculated by linear interpolation between the adjacent manually segmented slices. The interpolation tool was particularly useful when changes from slice-to-slice are small and progressive, like for the brain or ectodermal placodes. With the wrap selection function, the operator first created a scaffold by manually segmenting slices in the three orthogonal projections (XY, XZ, YZ) and then, an algorithm automatically computed the remaining slices. The wrap tool was especially helpful for structures with more complex three-dimensional shapes, like the somites. These tools considerably helped reduce the workload and increased the segmentation speed without impacting the accuracy [61], particularly important for large datasets such as the one provided here. Nonetheless, manual fine-tuning was necessary to ensure the accuracy of the segmentation in complex and fine structures. The manual segmentation of the individual embryonic elements took 1-5 days per sample for the smaller embryos (St.12-20) and up to 2 weeks for larger ones with more complex anatomy (St.21-31). Smoothing of 3D renderings was performed to reduce the staircase artifacts that appeared due to the manual segmentations of the structures in 2D slices.

Combination Validation of SRµCT data by confocal microscopy

The segmentation of different embryonic structures was performed based on morphological landmarks, contrast differences between adjacent tissues, and publicly available information on Chondrichthyes and vertebrate development. The segmented 3D reconstructions of the developing structures can be visualized simultaneously in the same model, which helps understand the spatial relationships between distinct tissues and organs (Fig. 3A). The segmented 3D models can be directly compared between consecutive developmental stages to understand the complex morphogenetic processes during embryogenesis together with organ growth and shaping. This approach can be applied to virtually any of the developing structures. Moreover, the obtained 3D models can be additionally integrated with other popular non-destructive 3D techniques such as confocal laser scanning microscopy with the use of immunofluorescence or HCR *in situ* hybridization (Fig. 3B, C). These two methods are essential tools in modern evo-devo research, enabling precise labelling of distinct cellular populations and anatomical structures within the developing embryo based on protein location and differential gene expression, respectively [62]. By combining the high-resolution SRμCT morphological data with fluorescence spatial gene expression mapping, we are able to establish a powerful framework to link genetic programs with tissue architecture and organismal morphology.

Reuse potential

There are numerous opportunities to employ this dataset in a wide spectrum of studies, allowing to analyse the formation of virtually any embryonic structure: cranial placodes (adenohypophyseal, olfactory, lens, trigeminal, profundal, lateral line, otic and epibranchial), sensory organs, teeth, skin, skin denticles, head cavities, neural crest cells, facial mesenchyme, cartilage, vertebral column, skull, gills, pharyngeal arches, pharyngeal pouches, somites, muscles, tendons, central nervous system (forebrain, midbrain, hindbrain and spinal cord), peripheral nervous system (cranial ganglia, dorsal root ganglia, nerves), endocrine organs, kidneys, stomach, liver, gut, heart, blood vessels, fins (pectoral, pelvic, dorsal, anal, caudal), and

reproductive system (see examples of different structures from representative developmental stages in Fig. 4). This dataset captures the early morphogenetic events that lead to the formation of the general chondrichthyan body plan from the three main embryonic germ layers (endoderm, mesoderm, ectoderm), till later stages of organogenesis, when species-specific differences begin to emerge among embryos of distinct chondrichthyan species [63]. The analysis of consecutive developmental stages allows researchers to reconstruct and trace the embryonic origin of the developing anatomical features, providing key insights into the developmental trajectories and formation of tissues and organs in Chondrichthyes. Moreover, during embryogenesis, tissues and organs arise and grow in a synchronous manner, establishing tight connections and integrating to form a functional organism. Such high-dimensional interactions are challenging to study by conventional 2D and 3D methods and require high-resolution non-destructive imaging techniques. This SRμCT dataset represents a comprehensive resource to resolve tissue architecture and the integration of embryonic structures in 3D space.

Chondrichthyes are well-known for their asynchronous development [26,31,64]; embryos from eggs laid at the same time might not develop at the same rate even under identical conditions (e.g., St.25 embryos range from 31 to 38 days after deposition) [31]. Hence, even though information on days-after deposition is a good measure of the approximate developmental stage, each embryo must be individually assessed based on the present morphological landmarks. Nonetheless, embryos at the same developmental stage often exhibit morphological differences, most notoriously in the number of somites (e.g., St.24 embryos possess between 64-78 somites). This dataset can be used to study the natural variability in Chondrichthyes development since it provides scans of at least two independent embryos from St.17-29, when differences in somite number are apparent [31].

Research on Chondrichthyes development is challenging due to the difficulties in obtaining fertilized eggs or embryonic material. The presented SRμCT dataset serves as a valuable and information-rich resource to investigate shark embryonic development and incorporate Chondrichthyes into modern comparative

embryology studies, enabling a deeper understanding of the divergence and conservation of the developmental programs shaping vertebrate morphological diversity.

Data availability

The SRµCT dataset behind this manuscript is available in the GigaScience Database repository. We provide the raw reconstructed SRµCT data together with the pre-processed files. Because of the large size of the raw reconstructed files, we recommend using the pre-processed data instead. If using the raw reconstructed files, please note that small misalignments of the vertical tiles may appear due to stitching artifacts and will need to be manually corrected by the operator. All data analysis and segmentations were performed on a workstation equipped with the following hardware components: an NVIDIA Quadro P5000 graphics processing unit (GPU), an Intel Xeon W-2145 central processing unit (CPU), and 64GB of system memory (RAM). Based on our experience with this configuration, we recommend resampling the files to obtained a final working image stack of less than 2GB of size, or ideally less than 1GB when extensive segmentation is expected. However, file size limitations may vary depending on the specification of the workstation used. The dataset is presented as TIFF stacks of the corresponding tomographic slices. Each folder contains the information for one single embryo. The naming of files is as follows: sample_name_32b.tiff for raw reconstructed files and sample_name_8b.tiff for pre-processed files. Information about the voxel size for each sample can be found in Supplementary Table 2. For the production, curation and analysis of this SRµCT dataset we used ImageJ/Fiji [60] and Avizo3D Pro Software. However, other software options are available to manipulate, visualize and analyze this dataset such as 3D Slicer [65], Amira (ThermoFisher Scientific), VG Studio MAX (Volume Graphics GmbH, Germany) or MeshLab [66].

List of abbreviations

µCT: micro-computed tomography; µm: micrometer; cm: centimeter; CPU, central processing unit; evo-devo: evolutionary developmental biology; FOV: field of view; h: hour; GB: gigabyte; GPU, graphics processing unit; HCR: hybridization chain reaction; min: minute; mm: millimeter; ms: millisecond; PBS: Dulbecco's Phosphate Buffered Saline; PFA: paraformaldehyde; PTA: phosphotungstic acid; RAM, random-access memory; St: stage; SRµCT: synchrotron radiation micro-computed tomography; TB: terabyte; UV: ultraviolet.

Declarations

All animal work was conducted following Directive 2010/63/EU, the German Animal Welfare Act (Tierschutzgesetz § 11) and in compliance with the Federation of European Laboratory Animal Science Associations' guidelines for the housing, handling and euthanasia of laboratory animals. The collection of small-spotted catshark embryonic stages used in this study does not require ethical permit and short-term housing of the eggs was approved by the local veterinary officer (Veterinäramt Kreis, Plön).

Consent for publication

Not applicable

Competing interests

The authors declare that they have no competing interests.

Funding

Not applicable

Author contributions

E.E.V.	Conceptualization, formal analysis, funding acquisition, investigation, visualization, writing – original draft, writing – review and editing.
A.K.	Investigation, resources, writing – review and editing.
L.W.G.S.	Data curation, investigation, writing – review and editing.
A.P.M.R.	Investigation, writing – review and editing.
S.K.	Investigation, writing – review and editing.
J.U.H.	Methodology, software, writing – review and editing.
T.M.	Investigation, resources, writing – review and editing.
M.K.	Conceptualization, funding acquisition, project administration, supervision, writing – original draft, writing – review and editing.

Acknowledgements

E.E.V., L.W.G.S., A.P.M.R., S.K., and M.K. were supported by the Max Planck Society. The authors would like to thank animal caretakers Erika Teßmann and Ulrich Friese for their assistance and dedication in caring for the small-spotted catsharks in Ozeaneum (Stralsund). The authors would also like to extend their gratitude to Martin Hansel and his team from Sea Life Berlin for donating the small-spotted catshark eggs. The authors acknowledge DESY (Hamburg, Germany), a member of the Helmholtz Association HGF, for the provision of experimental facilities where SRµCT was carried out at the PETRA III beamline P05. Beamtime was allocated for proposals I-20230087 and I-20240871.

References

1. Coolen M, Sauka-Spengler T, Nicolle D, Le-Mentec C, Lallemand Y, Silva CD, et al.. Evolution of Axis Specification Mechanisms in Jawed Vertebrates: Insights from a Chondrichthyan. *PLoS One*. 2007; doi: 10.1371/journal.pone.0000374.

2. Square T, Jandzik D, Cattell M, Coe A, Doherty J, Medeiros DM. A gene expression map of the larval Xenopus laevis head reveals developmental changes underlying the evolution of new skeletal elements. *Dev Biol*. 2015; doi: 10.1016/j.ydbio.2014.10.016.

3. Piekarski N, Gross JB, Hanken J. Evolutionary innovation and conservation in the embryonic derivation of the vertebrate skull. *Nat Commun*. Nature Publishing Group; 2014; doi: 10.1038/ncomms6661.

4. Bhullar B-AS, Morris ZS, Sefton EM, Tok A, Tokita M, Namkoong B, et al.. A molecular mechanism for the origin of a key evolutionary innovation, the bird beak and palate, revealed by an integrative approach to major transitions in vertebrate history. *Evolution*. 2015; doi: 10.1111/evo.12684.

5. Kalinka AT, Tomancak P. The evolution of early animal embryos: conservation or divergence? *Trends in Ecology & Evolution*. 2012; doi: 10.1016/j.tree.2012.03.007.

6. Carroll SB. Evo-Devo and an Expanding Evolutionary Synthesis: A Genetic Theory of Morphological Evolution. *Cell*. 2008; doi: 10.1016/j.cell.2008.06.030.

7. Tanaka Y, Kudoh H, Abe G, Yonei-Tamura S, Tamura K. Evo-Devo of the Fin-to-Limb Transition. *Evolutionary Developmental Biology*. Springer, Cham;

8. Irion U, Nüsslein-Volhard C. Developmental genetics with model organisms. *Proceedings of the National Academy of Sciences*. Proceedings of the National Academy of Sciences; 2022; doi: 10.1073/pnas.2122148119.

9. Jenner RA, Wills MA. The choice of model organisms in evo–devo. *Nat Rev Genet*. Nature Publishing Group; 2007; doi: 10.1038/nrg2062.

10. Nojiri T, Fukui D, Werneburg I, Saitoh T, Endo H, Koyabu D. Embryonic staging of bats with special reference to Vespertilio sinensis and its cochlear development. *Developmental Dynamics*. 2021; doi: 10.1002/dvdy.325.

11. Anthwal N, Urban DJ, Sadier A, Takenaka R, Spiro S, Simmons N, et al.. Insights into the formation and diversification of a novel chiropteran wing membrane from embryonic development. *BMC Biology*. 2023; doi: 10.1186/s12915-023-01598-y.

12. Bai S, Li S, Li X, Zhu S, Shan Z, Zhang J, et al.. Comparison of embryonic development, from HH21 to HH40, between ostrich (Struthio camelus) and chicken (Gallus gallus). *Developmental Dynamics*. 2023; doi: 10.1002/dvdy.568.

13. Curantz C, Bailleul R, Castro-Scherianz M, Hidalgo M, Durande M, Graner F, et al.. Cell shape anisotropy contributes to self-organized feather pattern fidelity in birds. *PLOS Biology*. Public Library of Science; 2022; doi: 10.1371/journal.pbio.3001807.

14. Pranter R, Feiner N. Spatiotemporal distribution of neural crest cells in the common wall lizard Podarcis muralis. *Developmental Dynamics*. 2025; doi: 10.1002/dvdy.758.

15. Diaz Jr RE, Shylo NA, Roellig D, Bronner M, Trainor PA. Filling in the phylogenetic gaps: Induction, migration, and differentiation of neural crest cells in a squamate reptile, the veiled chameleon (Chamaeleo calyptratus). *Developmental Dynamics*. 2019; doi: 10.1002/dvdy.38.

16. Stundl J, Pospisilova A, Matějková T, Psenicka M, Bronner ME, Cerny R. Migratory patterns and evolutionary plasticity of cranial neural crest cells in ray-finned fishes. *Developmental Biology*. 2020; doi: 10.1016/j.ydbio.2020.08.007.

17. Horackova A, Pospisilova A, Stundl J, Minarik M, Jandzik D, Cerny R. Pre-mandibular pharyngeal pouches in early non-teleost fish embryos. *Proceedings of the Royal Society B: Biological Sciences*. Royal Society; 2023; doi: 10.1098/rspb.2023.1158.

18. Shimeld SM, Donoghue PCJ. Evolutionary crossroads in developmental biology: cyclostomes (lamprey and hagfish). *Development*. 2012; doi: 10.1242/dev.074716.

19. Amaral CRL, Pereira ,Filipe, Silva ,Dayse A., Amorim ,António, and de Carvalho EF. The mitogenomic phylogeny of the Elasmobranchii (Chondrichthyes). *Mitochondrial DNA Part A*. Taylor & Francis; 2018; doi: 10.1080/24701394.2017.1376052.

20. Compagnucci C, Debiais-Thibaud M, Coolen M, Fish J, Griffin JN, Bertocchini F, et al.. Pattern and polarity in the development and evolution of the gnathostome jaw: Both conservation and heterotopy in the branchial arches of the shark, *Scyliorhinus canicula*. *Developmental Biology*. 2013; doi: 10.1016/j.ydbio.2013.02.022.

21. Ermakova GV, Meyntser IV, Zaraisky AG, Bayramov AV. Loss of noggin1, a classic embryonic inducer gene, in elasmobranchs. *Sci Rep*. Nature Publishing Group; 2024; doi: 10.1038/s41598-024-54435-9.

22. Gillis JA, Alsema EC, Criswell KE. Trunk neural crest origin of dermal denticles in a cartilaginous fish. *Proceedings of the National Academy of Sciences*. Proceedings of the National Academy of Sciences; 2017; doi: 10.1073/pnas.1713827114.

23. Cole NJ, Currie PD. Insights from sharks: Evolutionary and developmental models of fin development. *Developmental Dynamics*. 2007; doi: 10.1002/dvdy.21268.

24. : First observation of the mating, egg-laying and hatching behaviour of a captive female Kong skate, Okamejei kenojei (Müller & Henle, 1841) - Gao - 2022 - Journal of Fish Biology - Wiley Online Library. https://onlinelibrary.wiley.com/doi/full/10.1111/jfb.15165 Accessed 2025 Jun 6.

25. : The Elasmobranch Husbandry Manual: Captive Care of Sharks, Rays, and Their Relatives [1 ed.] 0867271523, 9780867271522. dokumen.pub. https://dokumen.pub/the-elasmobranch-husbandry-manual-captive-care-of-sharks-rays-and-their-relatives-1nbsped-0867271523-9780867271522.html Accessed 2025 Jun 6.

26. Gillis JA, Bennett S, Criswell KE, Rees J, Sleight VA, Hirschberger C, et al.. Big insight from the little skate: *Leucoraja erinacea* as a developmental model system. In: Goldstein B, Srivastava M, editors. *Current Topics in Developmental Biology*. Academic Press;

27. Maruska KP, Gelsleichter J. Chapter 11 - Hormones and Reproduction in Chondrichthyan Fishes. In: Norris DO, Lopez KH, editors. *Hormones and Reproduction of Vertebrates*. London: Academic Press;

28. Buddle AL, Dyke JUV, Thompson MB, Simpfendorfer CA, Whittington CM. Evolution of placentotrophy: using viviparous sharks as a model to understand vertebrate placental evolution. *Mar Freshwater Res*. CSIRO PUBLISHING; 2018; doi: 10.1071/MF18076.

29. Katona G, Szabó F, Végvári Z, Székely Jr T, Liker A, Freckleton RP, et al.. Evolution of reproductive modes in sharks and rays. *Journal of Evolutionary Biology*. 2023; doi: 10.1111/jeb.14231.

30. Pacoureau N, Rigby CL, Kyne PM, Sherley RB, Winker H, Carlson JK, et al.. Half a century of global decline in oceanic sharks and rays. *Nature*. Nature Publishing Group; 2021; doi: 10.1038/s41586-020-03173-9.

31. Ballard WW, Mellinger J, Lechenault H. A series of normal stages for development of *Scyliorhinus canicula* , the lesser spotted dogfish *(Chondrichthyes: Scyliorhinidae)*. *J Exp Zool*. 1993; doi: 10.1002/jez.1402670309.

32. Mayeur H, Leyhr J, Mulley J, Leurs N, Michel L, Sharma K, et al.. The Sensory Shark: High-quality Morphological, Genomic and Transcriptomic Data for the Small-spotted Catshark Scyliorhinus Canicula Reveal the Molecular Bases of Sensory Organ Evolution in Jawed Vertebrates. *Molecular Biology and Evolution*. 2024; doi: 10.1093/molbev/msae246.

33. Vidal-Vázquez N, Hernández-Núñez I, Carballo-Pacoret P, Salisbury S, Villamayor PR, Hervas-Sotomayor F, et al.. A single-nucleus RNA sequencing atlas of the postnatal retina of the shark Scyliorhinus canicula. *Sci Data*. Nature Publishing Group; 2025; doi: 10.1038/s41597-025-04547-2.

34. Mayeur H, Lanoizelet M, Quillien A, Menuet A, Michel L, Martin KJ, et al.. When Bigger Is Better: 3D RNA Profiling of the Developing Head in the Catshark Scyliorhinus canicula. *Front Cell Dev Biol*. Frontiers; 2021; doi: 10.3389/fcell.2021.744982.

35. Pearce J, Fraser MW, Sequeira AMM, Kaur P. State of Shark and Ray Genomics in an Era of Extinction. *Front Mar Sci*. Frontiers; 2021; doi: 10.3389/fmars.2021.744986.

36. Ziermann JM, Freitas R, Diogo R. Muscle development in the shark Scyliorhinus canicula: implications for the evolution of the gnathostome head and paired appendage musculature. *Frontiers in Zoology*. 2017; doi: 10.1186/s12983-017-0216-y.

37. Elagoz AM, Styfhals R, Maccuro S, Masin L, Moons L, Seuntjens E. Optimization of Whole Mount RNA Multiplexed in situ Hybridization Chain Reaction With Immunohistochemistry, Clearing and Imaging to Visualize Octopus Embryonic Neurogenesis. *Front Physiol*. Frontiers; 2022; doi: 10.3389/fphys.2022.882413.

38. Randlett O, Wee CL, Naumann EA, Nnaemeka O, Schoppik D, Fitzgerald JE, et al.. Whole-brain activity mapping onto a zebrafish brain atlas. *Nat Methods*. Nature Publishing Group; 2015; doi: 10.1038/nmeth.3581.

39. André M, Dinvaut S, Castellani V, Falk J. 3D exploration of gene expression in chicken embryos through combined RNA fluorescence in situ hybridization, immunofluorescence, and clearing. *BMC Biology*. 2024; doi: 10.1186/s12915-024-01922-0.

40. Matula J, Tesarova M, Zikmund T, Kaucka M, Adameyko I, Kaiser J. X-ray microtomography–based atlas of mouse cranial development. *GigaScience*. 2021; doi: 10.1093/gigascience/giab012.

41. Laznovsky J, Kavkova M, Helena Reis A, Robovska-Havelkova P, Maia LA, Krivanek J, et al.. Unveiling vertebrate development dynamics in frog Xenopus laevis using micro-CT imaging. *GigaScience*. 2024; doi: 10.1093/gigascience/giae037.

42. Metscher BD. MicroCT for developmental biology: A versatile tool for high-contrast 3D imaging at histological resolutions. *Developmental Dynamics*. 2009; doi: 10.1002/dvdy.21857.

43. Deeming DC, Kundrát M. Interpretation of fossil embryos requires reasonable assessment of developmental age. *Paleobiology*. 2023; doi: 10.1017/pab.2022.21.

44. Enault S, Adnet S, Debiais-Thibaud M. Skeletogenesis during the late embryonic development of the catshark Scyliorhinus canicula (Chondrichthyes; Neoselachii). *MorphoMuseum*. Association Palæovertebrata; 2016; doi: 10.18563/m3.1.4.e2.

45. Metscher BD. MicroCT for comparative morphology: simple staining methods allow high-contrast 3D imaging of diverse non-mineralized animal tissues. *BMC Physiology*. 2009; doi: 10.1186/1472-6793-9-11.

46. Callahan S, Crowe-Riddell JM, Nagesan RS, Gray JA, Davis Rabosky AR. A guide for optimal iodine staining and high-throughput diceCT scanning in snakes. *Ecology and Evolution*. 2021; doi: 10.1002/ece3.7467.

47. Babaei F, Hong TLC, Yeung K, Cheng SH, Lam YW. Contrast-Enhanced X-Ray Micro-Computed Tomography as a Versatile Method for Anatomical Studies of Adult Zebrafish. *Zebrafish*. Mary Ann Liebert, Inc., publishers; 2016; doi: 10.1089/zeb.2016.1245.

48. Criswell KE, Coates MI, Gillis JA. Embryonic development of the axial column in the little skate, *Leucoraja erinacea*. *Journal of Morphology*. 2017; doi: 10.1002/jmor.20637.

49. Gabner S, Böck P, Fink D, Glösmann M, Handschuh S. The visible skeleton 2.0: phenotyping of cartilage and bone in fixed vertebrate embryos and foetuses based on X-ray microCT. *Development*. 2020; doi: 10.1242/dev.187633.

50. Theodosiou NA, Oppong E. 3D morphological analysis of spiral intestine morphogenesis in the little skate, Leucoraja erinacea. *Developmental Dynamics*. 2019; doi: 10.1002/dvdy.34.

51. Rzhepakovsky I, Piskov S, Avanesyan S, Shakhbanov M, Sizonenko M, Timchenko L, et al.. High-Performance Microcomputing Tomography of Chick Embryo in the Early Stages of Embryogenesis. *Applied Sciences*. Multidisciplinary Digital Publishing Institute; 2023; doi: 10.3390/app131910642.

52. Tafforeau P, Boistel R, Boller E, Bravin A, Brunet M, Chaimanee Y, et al.. Applications of X-ray synchrotron microtomography for non-destructive 3D studies of paleontological specimens. *Appl Phys A*. 2006; doi: 10.1007/s00339-006-3507-2.

53. Betz O, Wegst U, Weide D, Heethoff M, Helfen L, Lee W-K, et al.. Imaging applications of synchrotron X-ray phase-contrast microtomography in biological morphology and biomaterials science. I. General aspects of the technique and its advantages in the analysis of millimetre-sized arthropod structure. *Journal of Microscopy*. 2007; doi: 10.1111/j.1365-2818.2007.01785.x.

54. Tesařová M, Mancini L, Simon A, Adameyko I, Kaucká M, Elewa A, et al.. A quantitative analysis of 3D-cell distribution in regenerative muscle-skeletal system with synchrotron X-ray computed microtomography. *Sci Rep*. Nature Publishing Group; 2018; doi: 10.1038/s41598-018-32459-2.

55. Leyhr J, Sanchez S, Dollman KN, Tafforeau P, Haitina T. Enhanced contrast synchrotron X-ray microtomography for describing skeleton-associated soft tissue defects in zebrafish mutants. *Front Endocrinol*. Frontiers; 2023; doi: 10.3389/fendo.2023.1108916.

56. Wilde F, Ogurreck M, Greving I, Hammel JU, Beckmann F, Hipp A, et al.. Micro-CT at the imaging beamline P05 at PETRA III. *AIP Conference Proceedings*. 2016; doi: 10.1063/1.4952858.

57. Moosmann J, Ershov A, Weinhardt V, Baumbach T, Prasad MS, LaBonne C, et al.. Time-lapse X-ray phase-contrast microtomography for in vivo imaging and analysis of morphogenesis. *Nat Protoc*. Nature Publishing Group; 2014; doi: 10.1038/nprot.2014.033.

58. van Aarle W, Palenstijn WJ, De Beenhouwer J, Altantzis T, Bals S, Batenburg KJ, et al.. The ASTRA Toolbox: A platform for advanced algorithm development in electron tomography. *Ultramicroscopy*. 2015; doi: 10.1016/j.ultramic.2015.05.002.

59. Aarle W van, Palenstijn WJ, Cant J, Janssens E, Bleichrodt F, Dabravolski A, et al.. Fast and flexible X-ray tomography using the ASTRA toolbox. *Opt Express, OE*. Optica Publishing Group; 2016; doi: 10.1364/OE.24.025129.

60. Schindelin J, Arganda-Carreras I, Frise E, Kaynig V, Longair M, Pietzsch T, et al.. Fiji: an open-source platform for biological-image analysis. *Nat Methods*. Nature Publishing Group; 2012; doi: 10.1038/nmeth.2019.

61. Tesařová M, Zikmund T, Kaucká M, Adameyko I, Jaroš J, Paloušek D, et al.. Use of micro computed-tomography and 3D printing for reverse engineering of mouse embryo nasal capsule. *J Inst*. 2016; doi: 10.1088/1748-0221/11/03/C03006.

62. Choi HMT, Schwarzkopf M, Fornace ME, Acharya A, Artavanis G, Stegmaier J, et al.. Third-generation in situ hybridization chain reaction: multiplexed, quantitative, sensitive, versatile, robust. *Development*. 2018; doi: 10.1242/dev.165753.

63. Byrum SR, Frazier BS, Grubbs RD, Naylor GJP, Fraser GJ. Embryonic development in the bonnethead (Sphyrna tiburo), a viviparous hammerhead shark. *Developmental Dynamics*. 2024; doi: 10.1002/dvdy.658.

64. Onimaru K, Motone F, Kiyatake I, Nishida K, Kuraku S. A staging table for the embryonic development of the brownbanded bamboo shark (Chiloscyllium punctatum). *Developmental Dynamics*. 2018; doi: 10.1002/dvdy.24623.

65. Fedorov A, Beichel R, Kalpathy-Cramer J, Finet J, Fillion-Robin J-C, Pujol S, et al.. 3D Slicer as an image computing platform for the Quantitative Imaging Network. *Magn Reson Imaging*. 2012; doi: 10.1016/j.mri.2012.05.001.

66. Cignoni P, Callieri M, Corsini M, Dellepiane M, Ganovelli F, Ranzuglia G. MeshLab: an Open-Source Mesh Processing Tool. The Eurographics Association;

Figure legends

Figure 1: Experimental setup for SRµCT of small-spotted catshark embryos. (A) Fertilized small-spotted catshark eggs. White arrowhead indicates an embryo at approximately St.28 within the eggcase. Scale bar: 1cm. (B) St.31 small-spotted catshark embryo, attached to its yolk sac and removed from the eggcase. Scale bar: 1cm. (C) PTA-contrasted small-spotted catshark embryos at two developmental stages: St.28 (left), and St.15 (right, indicated by black arrowhead). The embryos are mounted in plastic pipette tips and glued to standardized holders. Scale bar: 1cm. (D) Experimental setup at the beamline P05 at PETRA III. Red arrowhead indicates the sample positioned in the center of the stage prior to imaging. (E) St.15 small-spotted catshark embryo from Fig. 1C immediately after scanning. Scale bar: 1.5mm. (F) Raw tomographic slice of PTA-contrasted St.30 small-spotted catshark embryo (left) and the same tomographic

slice after pre-processing in ImageJ/Fiji (right). The dotted white circles indicate the pipette tip containing the embryo. Scale bar: 4mm.

Figure 2: 3D reconstruction of a fully segmented St.19 small-spotted catshark embryo. (A) 3D reconstruction of all segmented structures together. Note that some structures may not visible in this model because they are located beneath other anatomical structures. Scale bar: 500µm. (B) 3D reconstructions of segmented structures separated in distinct models, which allows for better visualization of all of them. Scale bar: 500µm.

Figure 3: Combination of SRµCT with 3D confocal laser microscopy gene expression data. (A) 3D reconstruction of the developing small-spotted catshark nervous system at St.23. Blue 3D models represent the segmented central nervous system (CNS). Red 3D models represent the segmented peripheral nervous system (PNS). Scale bars: 1.5mm. A' and A'' indicate tomographic slices of the pre-processed SRµCT data used to reconstruct the nervous system. Scale bar: 250µm. (B) TUJ1 immunofluorescence labelling the PNS and immature neurons of the CNS at St.23. Scale bar: 1.5mm. (C) HCR *in situ* hybridization labelling different subpopulations within the developing nervous system at St.23. Scale bar: 1mm.

Figure 4: Examples of 3D reconstructions and tomographic slices of small-spotted catshark embryos at different developmental stages. Yellow labelling indicates distinct developing structures. Red dashed lines indicate the anatomical position corresponding to the tomographic slices. (A) St.12 small-spotted catshark embryo. Dorsal view 3D reconstruction (left) and tomographic slice (right). Scale bars: 500µm. (B) St.15 small-spotted catshark embryo. Dorsal view 3D reconstruction (left) and tomographic slices (right). Top

tomographic slice through the prospective head region shows the elevation of the neural plate at this stage. Lower tomographic slice through the trunk region shows the formation of early developmental structures like the somites and primitive gut. Scale bars: 500µm. (C) St.25 small-spotted catshark embryo. Whole embryo lateral view 3D reconstruction (left) and close-up of the head region (middle). Scale bars: 1mm. Tomographic slice through the trunk region, where the pectoral fin buds are located, and close-up (right). Scale bars: 500µm. (D) Ventral and lateral view 3D reconstruction of the head of a St.31 small-spotted catshark embryo (left). Tomographic slice through the pharyngeal area showing the internal gills. Scale bars: 5mm.

Click here to access/download;Figure;Fig1.pdf

Figure 1

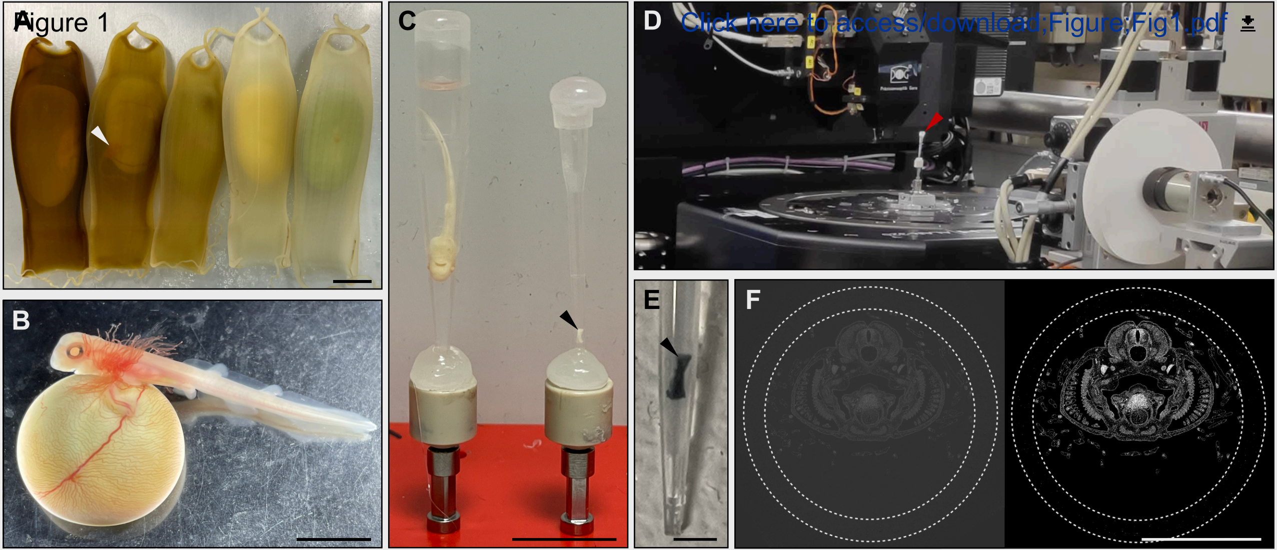

Click here to access/download;Figure;Fig2.pdf

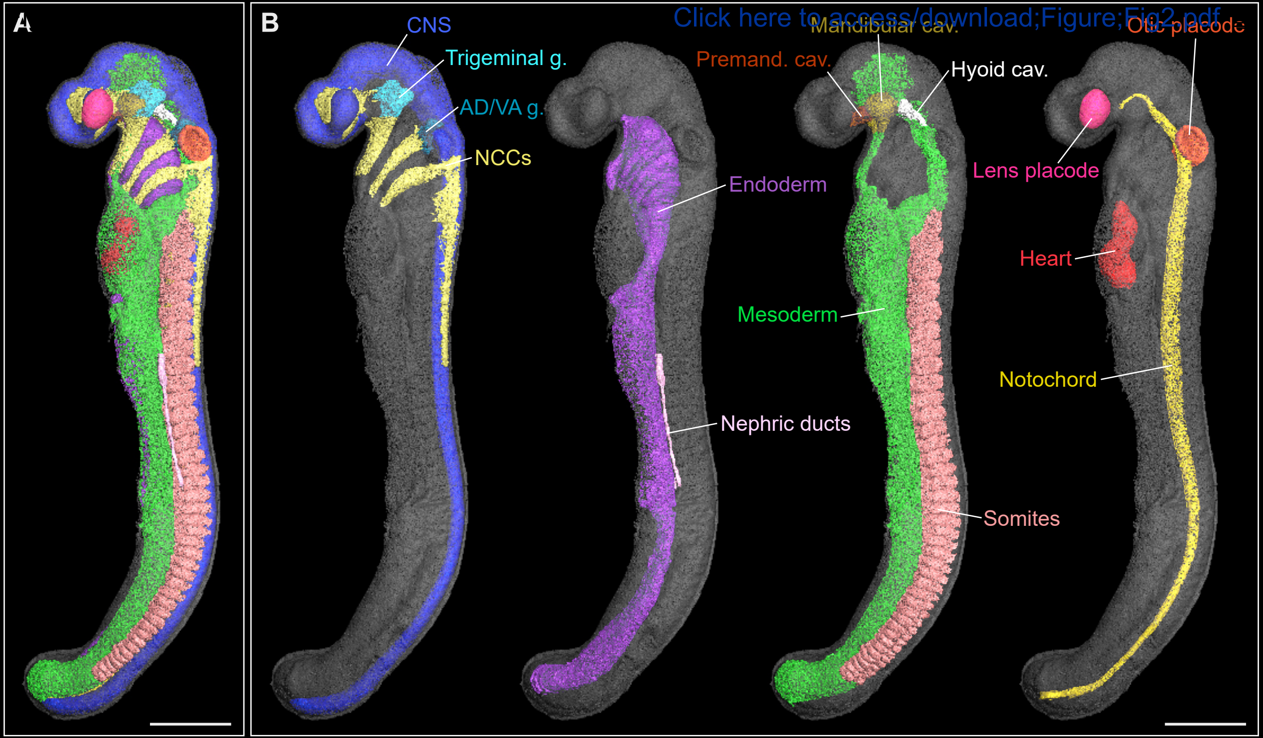

Click here to access/download;Figure;Fig3.pdf

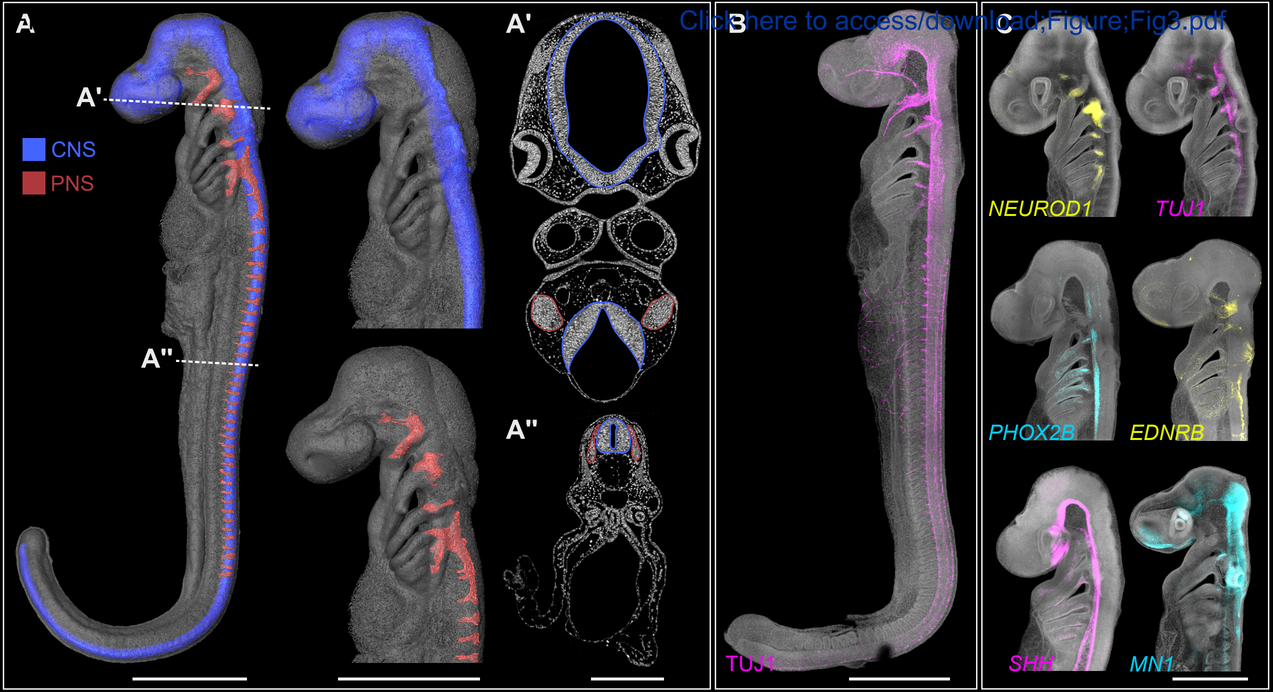

Click here to access/download;Figure;Fig4.pdf

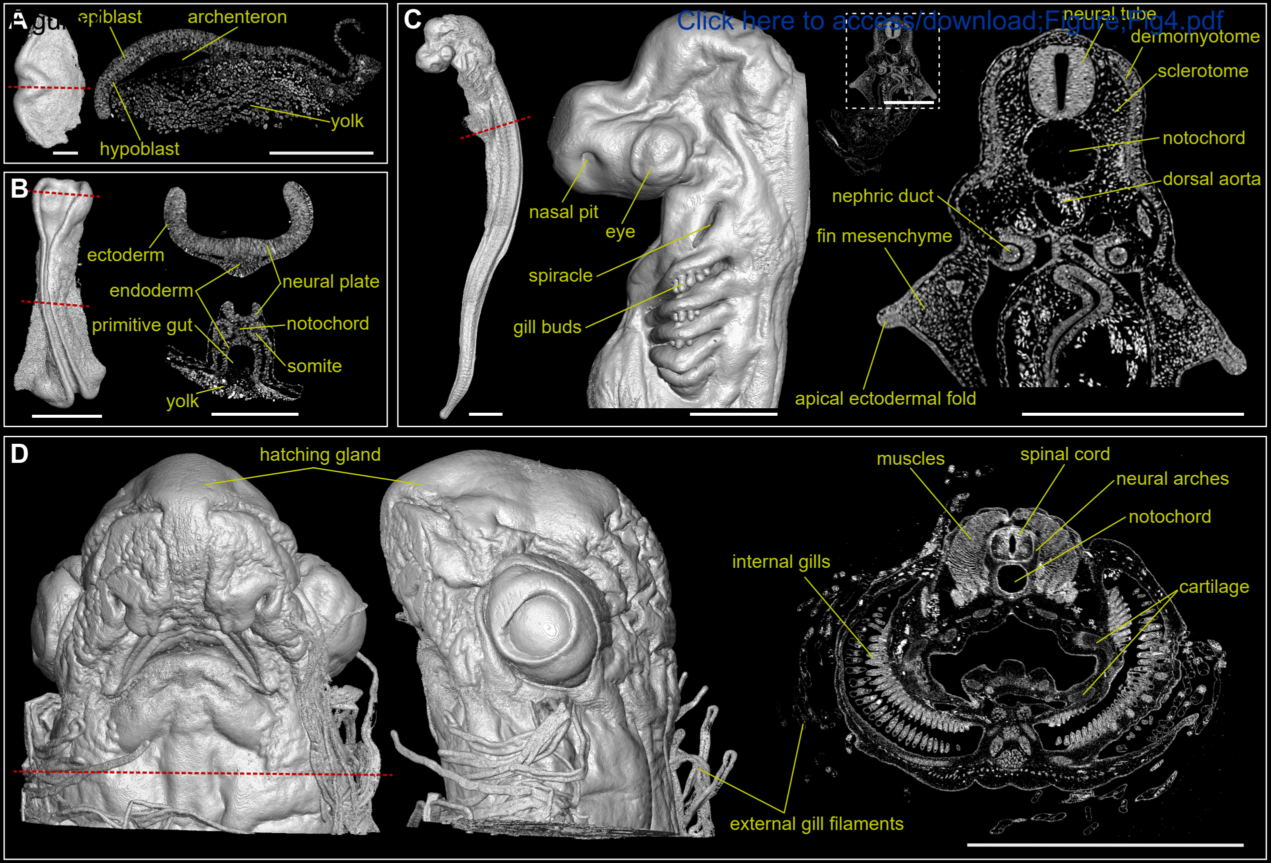

Click here to access/download
Supplementary Material
Supplementary_GigaScience_Final.docx

Cover letter

Click here to access/download;Personal Cover;CoverLetter_Gigascience_Final.docx

MAX PLANCK INSTITUTE
FOR EVOLUTIONARY BIOLOGY

To: Editorial Board
GigaScience

Dr. Markéta Kaucká

Max Planck Research Group Leader
Evolutionary Developmental Dynamics

MPI for Evolutionary Biology• August-Thienemann-Str. 2 • D-24306 Plön

Tel.: 00 49-(0) 15203 100 942
kaucka@evolbio.mpg.de

August 8, 2025

Dear Editors of *GigaScience*,

We are pleased to present our manuscript entitled "Synchrotron radiation micro-computed tomography of the small-spotted catshark embryonic development (Chondrichthyes: *Scyliorhinus canicula*)" for your consideration.

This manuscript presents a comprehensive 3D imaging dataset of embryonic development in the small-spotted catshark, generated using synchrotron-radiation micro-computed tomography (SRµCT). The dataset spans from the early embryonic development (stage 12, gastrula) till late embryogenesis (stage 31, organs and tissues are present and patterned). As such, the presented work provides high-resolution volumetric data that captures the emergence of internal and external anatomical structures across development.

The data will be deposited and made available to the community in accordance with *GigaScience's* requirements, to support reuse in a wide range of research contexts, allowing the researchers to explore shark development and vertebrate evolution.

We believe this work is a strong fit for *GigaScience* due to its emphasis on high-quality data publication and reproducibility. The dataset is of particular value to researchers in evolutionary developmental biology (EvoDevo), comparative anatomy, and vertebrate evolution. It enables detailed investigation of developmental timing, organogenesis, and morphogenesis, while also supporting analyses of evolutionary innovations and conserved traits across vertebrates. By providing such comprehensive dataset of shark embryogenesis—a lineage that occupies a key phylogenetic position among jawed vertebrates—it facilitates testing hypotheses on the origins and diversification of vertebrate traits. Thus, we are confident that our manuscript will capture the attention of the *GigaScience* readership.

We confirm that the manuscript has not been published or submitted for publication elsewhere, and all authors have reviewed and approved the submitted version. We declare no competing interests. AI-assisted technologies were not used in the drafting of this manuscript. There are no issues related to journal policies requiring further disclosure and this submission is not part of a special issue.

We cordially thank you for considering our work for publication in *GigaScience* and for your time! Please don't hesitate to contact me with any question you may have.

With best wishes

Markéta
(on behalf of all authors)

Kaucká Markéta
