# Supplementary material for: Synchrotron radiation micro-computed tomography of the small-spotted catshark embryonic development (Chondrichthyes: Scyliorhinus canicula)
Source: Gigascience. 2026 May 7;15:giag054. doi: 10.1093/gigascience/giag054 (PMC13240850; doi:10.1093/gigascience/giag054)
Supplement: giag054_GIGA-D-25-00317_revision_1 [file giag054_giga-d-25-00317_revision_1.pdf]

GigaScience

Synchrotron radiation micro-computed tomography of the small-spotted catshark embryonic development (Chondrichthyes: Scyliorhinus canicula)

--Manuscript Draft--

**Manuscript Number:**	GIGA-D-25-00317R1
**Full Title:**	Synchrotron radiation micro-computed tomography of the small-spotted catshark embryonic development (Chondrichthyes: Scyliorhinus canicula)
**Article Type:**	Data Note
**Funding Information:**	Deutsches Elektronen-Synchrotron (I-20230087 and I-20240871) — Dr. Marketa Kaucka
**Abstract:**	Background<br><br>Sharks occupy a key position on vertebrate phylogeny, making them essential for understanding the early origins of jawed vertebrates (gnathostomes) and the functional adaptation of vertebrate traits like jaws or complex sensory systems. As such, sharks are important model organisms in evolutionary developmental biology (evo-devo), but sparse data and limited availability of samples hinder their inclusion in contemporary evo-devo research. The knowledge of their distinctive morphology will not only shed light on the anatomical architecture and physiology of basal living gnathostomes but also reveal the evolutionary divergence of developmental processes that establish the foundational vertebrate blueprint.<br><br>Findings<br><br>We performed synchrotron radiation micro-computed tomography (SRµCT) scanning of the small-spotted catshark (Scyliorhinus canicula) embryonic development, spanning from gastrulation (stage 12) to late-organogenesis (stage 31), enhanced by tissue contrasting with phosphotungstic acid. We obtained 36 whole-embryo scans that encompass the formation of key embryonic structures, such as sensory organs, fins, muscles and skeletal elements. The achieved resolution allows for segmentation of all tissue types and both internal and external structures.<br><br>Conclusions<br><br>We present a comprehensive dataset of 4D high-resolution SRµCT of the small-spotted catshark embryonic development. The dataset spans consecutive embryonic stages, allowing the reconstruction and morphometric analyses of tissues, organs, and structures, along with the tracking of their development. The deposited data are publicly available, and provide a valuable resource for comparative research, additionally allowing the identification of conserved and derived developmental processes and features and understanding the evolution of vertebrates.<br><br>Issue section<br><br>Data Note
**Corresponding Author:**	Marketa Kaucka, PhD<br>Max Planck Institute for Evolutionary Biology: Max-Planck-Institut fur Evolutionsbiologie<br>Ploen, GERMANY
**Corresponding Author Secondary Information:**	
**Corresponding Author's Institution:**	Max Planck Institute for Evolutionary Biology: Max-Planck-Institut fur Evolutionsbiologie
**Corresponding Author's Secondary Institution:**	
**First Author:**	Elio Escamilla-Vega
**First Author Secondary Information:**	

Powered by Editorial Manager® and ProduXion Manager® from Aries Systems Corporation

**Order of Authors:**	Elio Escamilla-Vega
	Ann-Katrin Koch
	Louk W. G. Seton
	Andrea P. Murillo-Rincón
	Stella Kyomen
	Jörg U. Hammel
	Timo Moritz
	Marketa Kaucka
**Order of Authors Secondary Information:**	
**Response to Reviewers:**	Response to the Reviewers<br>We thank the Reviewers for their careful and thoughtful evaluation of our manuscript and for their constructive feedback. We have revised the manuscript accordingly and responded to each of their comments below.<br><br>Reviewer #1<br><br>The authors describe a 3D developmental image series of Scyliorhinus canicula, an important research animal. This is certainly a valuable data set, well documented and accessible. The images are of sufficient completeness and detail to be useful to other researchers. X-ray microtomographic images are an excellent complement to other modes of visualization. The report is well written and sufficient for the purpose.<br><br>We would like to cordially thank Reviewer #1 for their positive assessment of our manuscript and for their valuable and constructive suggestions.<br><br>A few specific items that merit attention:<br><br>p. 7: Sample shrinkage is always an issue with whole-mount imaging of soft specimens and PTA is well known to shrink tissues significantly. Were there any measurements of shrinkage or distortion, or comparisons with unstained samples? This would be worth mentioning.<br><br>Tissue shrinkage is indeed a known, unavoidable technical limitation of PTA-contrasting [1–3], and a slight tissue distortion is expected across samples and tissues. However, tissue shrinkage does not result in differences in the relative positions of organs or in the order of their appearance during embryogenesis.<br>We absolutely agree with the reviewer that it would have been very valuable to scan all the embryos prior to PTA-contrasting; however, due to the strict time limitations imposed by the granted funding and assigned beamtime, we could not carry out such scans and measurements to focus specifically on this issue. We now explicitly mention this phenomenon in the text and point the readers to the known effect of contrasting agents and tissue shrinkage (see lines 227-234 or quoted text below). Additionally, we included a new Supplementary Figure showing examples of tissue distortion (Supplementary Fig. 2).<br><br>“A slight tissue shrinkage was observed due to the overall tissue dehydration and PTA-contrasting enhancement (1.5% PTA in 90% methanol), which represents a known, unavoidable technical limitation of enhanced soft tissue contrast [65,81,82]. This effect was particularly evident in ectodermal structures starting at St.25 (Supplementary Fig. 2). However, despite the slight tissue shrinkage, the overall morphology and relative anatomical position of the different embryonic structures remain intact. Moreover, the relative order in which individual structures emerge along the developmental timeline are not changed, important for studies on developmental heterochrony.”<br><br>Line 163: Degrees are more conventional in practical measurements of angles<br><br>Thank you for this suggestion, we have modified the text and added the information in degrees.

Powered by Editorial Manager® and ProduXion Manager® from Aries Systems Corporation

p. 8 lines 187f.: Could say a bit more about how the brightness and contrast ranges were chosen: e.g. to suppress background, how much saturation, and refer to Fig. 1 F.

In response to your comment, we have incorporated an additional Supplementary Figure (Supplementary Fig. 1) where we show and explain how the brightness and contrast were adjusted using the Brightness/Contrast function in ImageJ/Fiji 2.9.0 following the guidelines from [4]. This function allows for modifying (clipping) the intensity range (maximum and minimum values) but does not provide information about saturation. We have also included the intensity thresholds selected for the original and pre-processed tomographic slices in Fig. 1F.

p. 11 lines 257-9 Two specimens are not enough to claim more than a baseline o starting point for studying variation.

We agree with the reviewer and have removed the statement from the text. Instead, we now emphasize the potential use of the dataset to investigate within-stage differences in future studies (see lines 309-316 or quoted text below). This is accompanied by a new Supplementary Figure (Supplementary Fig. 3) in which we present an example of within-stage differences, namely neural tube closure at early- and mid-St.17, to bring this topic to the attention of the readers.

“Small-spotted catshark embryos at the same developmental stage often exhibit slight morphological differences depending on their precise developmental timing. For instance, early St.26 embryos (approximately 37 days after deposition) have approximately 85 pairs of somites, while late St.26 embryos (approximately 42 days after deposition) have around 90 pairs of somites [41]. This within-stage variation highlights that embryonic development is a continuous process despite being conventionally subdivided into discrete stages. The presented dataset can serve as a starting point for future studies of within-stage developmental differences since it provides scans of at least two independent embryos from St.17-29, allowing a more refined characterization of small-spotted catshark development (Supplementary Fig. 3).”

p. 12 line 277 Do the archived datasets include the label files (the segmentations)? Good to mention this as it increases the value of the image data.

The datasets include the raw and pre-processed tomographic images, but not the segmentation files. Given the dataset's scientific relevance, we wanted to make it publicly available as soon as possible and provide a valuable resource to the evo-devo community. We agree with the Reviewer that providing the segmentations would increase the value of the dataset, and we are currently working towards an extensive segmentation resource. However, due to the large dataset spanning several developmental stages and the numerous embryonic structures to be segmented, these data are far from complete. All such segmentations, accompanied by extensive molecular mapping, will be summarized in a separate manuscript and made publicly available in the future.

Are the original specimens still intact and vouchered somewhere?

The PTA-contrasted embryos were not retained for long-term storage after SRµCT imaging. During synchrotron scanning, the specimens received a high X-ray dose, which is known to induce radiolysis of water and organic material and to chemically modify PTA (visible as the formation of “tungsten blue”) (Fig. 1E). These radiation-induced changes can alter both the tissue and the staining chemistry and may continue to develop during further storage, making the specimens unsuitable for subsequent quantitative imaging, histology, or other analyses.
In addition, PTA staining is not readily reversible, and prolonged storage of contrasted/fixed/scanned embryonic material often leads to further diffusion of stain, precipitation, or tissue brittleness, which compromises morphology. After weighing the pros and cons of further storage, we opted to discard these samples safely.

Fig. 3F: Thank you for showing pre- and post-processed images.

We thank the Reviewer for the positive feedback.

Powered by Editorial Manager® and ProduXion Manager® from Aries Systems Corporation

Reviewer #2

This manuscript presents a high-resolution dataset of embryonic development in the small-spotted catshark (Scyliorhinus canicula), an emerging important model for studying vertebrate development and evolution. Using synchrotron radiation micro-computed tomography (SRμCT), 36 whole-embryo scans were obtained across key developmental stages (gastrulation to late organogenesis, stages 12-31). Enhanced tissue contrast with phosphotungstic acid enabled high-level resolution, allowing semi-automated reconstruction and pre-processing for 3D segmentation of anatomical structures during the development. The dataset has a potential to provide a comprehensive resource for morphometric analyses and comparative evo-devo research, facilitating the identification of conserved and derived developmental processes in vertebrates.

This dataset has a great potential to enhance the evo-devo research on catshark, however, there are several major points that need to be addressed by the authors.

We would like to cordially thank Reviewer #2 for their positive assessment of the manuscript and for their valuable comments and suggestions, which helped us improve its clarity and address its limitations.

Major points:

1. It is important to make it clear that this is not the first attempt to image catshark embryos with a synchrotron radiation micro-computed tomography by using enhanced tissue contrast. For example, the recent paper by Mayeur et al (2024) described the 3D segmentation of the catshark late embryo sensory structures obtained by this technique using another contrast agent. Even though the authors cite this paper, it is not made clear in the text. However, I agree that this a first catshark embryo dataset spanning a large range of developmental stages that provides a great comparative potential.

We thank the Reviewer for pointing this out and apologize for not discussing the publication by Mayeur et al. (2024) sufficiently. While the publication was indeed cited in the original manuscript, we agree that its relevance was not adequately discussed. We have introduced the following text to acknowledge its contributions.

“Synchrotron radiation micro-computed tomography (SRμCT) combined with iodine-based contrast enhancement was previously employed to reconstruct and characterize the sensory organs in a pre-hatching small-spotted catshark embryo [42], highlighting the potential of this imaging technology to study chondrichthyan development in great detail.”

2. Catshark is an important vertebrate model, however it is very derived and during the evolution of vertebrates cartilaginous fishes lost perichondral, all major dermal and possibly even the endochondral bones (Ryll et al., 2014; Brazeau et al., 2020). This is important to add to the description of the species, to be able to describe for example the skeletal structures in the catshark embryo dataset.

We agree with the reviewer and, in response to this comment, we have added information on the evolution of skeletal tissues to the description of Chondrichthyes.

“Chondrichthyans are primarily known for having a cartilaginous endoskeleton throughout their lives, and the loss of perichondral, endochondral, and most dermal bones [26–29]. These traits render them important models for the research of skeletal tissue evolution.”

3. The information on image resolution, tissue enhancement properties of PTA and identified anatomical structures requires editing.

3.1 Authors state in several places in the text that they have acquired a dataset with a

Powered by Editorial Manager® and ProduXion Manager® from Aries Systems Corporation

cellular resolution, however this would require a resolution below 1 micron. It is also visible from the virtual histology sections on Figure 3 and 4 for example, that the resolution in not at the cellular level. This statement should be changed. In addition, a virtual histology section on Figure 4D has a lower resolution comparing to other virtual histology sections presented and should be replaced, if possible.

We agree with the reviewer and have carefully revised the manuscript and removed all references to "cellular resolution". We also apologize for the lower resolution of the tomographic slice shown in Fig. 4D and have replaced it with a higher-quality one.

We would like to note that scans from St.26-31 were acquired at a lower resolution (2.57µm) than those from St.12-25 (1.28µm), which likely explains why the tomographic slice in Fig. 4D appears slightly different from the others. However, we have ensured that the revised figure/panels are representative and of an appropriate quality.

3.2 It should be made clear in the text that PTA might not provide a good contrast for all types of tissues. In this dataset for example it is not well suitable for contrasting skeletal tissues, like muscle, and cartilage, which is visible on virtual histology sections on Figure 4.
Showing a few corresponding slides from a traditional histology for comparison would be valuable if authors would like to discuss PTA contrast as universal for all tissue types.

Thank you for this comment, we have clarified in the manuscript that different contrasting agents exhibit distinct tissue-specific absorption rates and contrasting properties, and we explicitly note that PTA does not stain cartilage – please see this adapted text:

"This limitation can be overcome by using contrasting agents, which are differentially absorbed by soft tissues, allowing their visualization in the final images [55]. Various contrast-enhancing agents have been tested, including iodine [51,56–58], ruthenium red [59], and phosphotungstic acid (PTA) [50,60,61], each with distinct tissue-specific absorption rates and, thus, contrasting abilities (e.g., PTA does not stain cartilage) [62–65]."

However, we would like to note that PTA has previously been successfully applied to the study of skeletal tissues, specifically, because cartilage remains uncontrasted while the surrounding softer tissues exhibit strongly enhanced contrast, allowing the identification and segmentation of skeletal structures (e.g., cartilage) based on the negative contrast [1,5–7]. We would like to further note that muscles do absorb PTA, as shown in the tomographic slice in Fig. 4 and in other publications [8].

Given the existing literature on this topic and because the aim of this manuscript is to present the small-spotted catshark developmental SRµCT dataset (rather than to compare or evaluate contrasting agents), we refrained from including traditional histological sections. We provide relevant references describing the benefits and disadvantages of PTA and contrasting different tissues in the manuscript.

3.3 There are no labeled teeth, skin denticles, tendons, liver, reproductive organs in the Figures, despite that the authors have included these tissues in a long list of tissues and structures that can be analyzed in this dataset (Lines 232-239). The suggestion is to edit this list of tissues and structures to include only the identified tissues in the dataset and all tissues mentioned should be also labeled in the Figures.

We thank the Reviewer for pointing out that the list of tissues and structures (lines 232-239, now 291-296) could be interpreted as referring only to structures explicitly labelled in the figures. Our intention was different: this list was meant to indicate the broader range of anatomical features that can be identified in the full dataset and therefore its potential for reuse, whereas the figures were designed only to show representative examples of some of the identifiable structures rather than to provide a comprehensive anatomical atlas.
Several tissues listed (e.g., teeth, skin denticles, liver, and reproductive organs) are present in the dataset at their respective developmental stages and anatomical

regions, but were not specifically labelled in the figures to maintain figure clarity and readability. Labelling all identifiable structures across multiple stages would substantially reduce legibility without improving the interpretation of the example images.

To avoid confusion, we have revised the manuscript to clarify that (i) the list of tissues and structures refers to features that can be identified in the dataset as a whole and highlights potential avenues for reuse, and (ii) the figures illustrate selected representative structures rather than an exhaustive inventory. We believe this revision addresses the Reviewer’s concern while preserving both figure clarity and the intended description of the dataset’s scope.

4. Include mid-lateral virtual histology sections on Figure 3 and 4?

We thank the Reviewer for this suggestion. A mid-lateral virtual histology section has now been included in Fig. 4D to provide an additional anatomical perspective. We have not added a comparable section to Fig. 3 because the figure is already densely populated, and inclusion of an additional panel would reduce clarity and readability. In Fig. 3, the coronal sections were intentionally chosen, as they provide a clearer visualization of both central and peripheral components of the nervous system, which are the primary features highlighted in this figure. We hope that the additional section in Fig. 4, together with the existing views in Fig. 3, provides sufficient anatomical context while maintaining figure clarity.

5. Because the authors emphasize that "The segmented 3D models can be directly compared between consecutive developmental stages to understand the complex morphogenetic processes during embryogenesis together with organ growth and shaping.", by providing a 3D reconstruction of several consecutive stages showing organ growth and shaping changes (for example inner organs like heart and additional stages with segmented CNS) they would demonstrate this. The stages should be clearly indicated in the Figures and not only in the Figure legend, which will also make a comparative aspect more pronounced.

We fully agree with the Reviewer and have now included a new main figure (Fig. 5) illustrating the process of heart morphogenesis. In this figure, we segmented the developing heart from St.19 to St.25 and show how it changes from an initial near-straight tube, through an S-shape, to a compact structure more similar to its final morphology. In addition, following the Reviewer’s suggestion, we have clearly indicated the corresponding developmental stages directly within the figure (and not only in the figure legend), as well as in all other figures.

6. Additional information is required for the growth series provided in Supplementary Table 2, this should include the length of the specimen and other important parameters like somite number.

We have incorporated embryo length and somite number into Supplementary Table 2. Although counting somites in early-developing catshark embryos is relatively easy, at later developmental stages, the anterior somites begin to differentiate, and only the posterior ones are recognizable. For this reason, and to avoid introducing potential errors, somite counts are provided up to St.25.

7. Author state that all data are publicly available for further exploration. However, there was no link available for reviewer for deposited raw or pre-processed small-size datasets. Therefore, the availability of all datasets described in Supplementary Table 2 could not be confirmed.
It is important that the authors make available the image stacks before and after processing i.e. of large and small size, since file size reduction might have resulted in the image quality reduction.

We sincerely apologize and regret that the Reviewer was unable to access the dataset during the review process. Due to the large size of the raw dataset, we were initially instructed by the editorial office (specifically the data curator) to provide only the pre-processed files. These files should have been made available to the Reviewers.

	We fully agree with the Reviewer that both raw image stacks and pre-processed datasets should be available. Accordingly, we have ensured that both the raw datasets and the pre-processed smaller files have been deposited in the Electron Microscopy Public Image Archive (EMPIAR). The datasets are assigned accession number EMPIAR-12984 and will be publicly accessible upon publication of the manuscript, in accordance with EMPIAR release policies. We are also contacting the journal to confirm that reviewer access is enabled where possible, so that the files can be examined during the review process.<br><br>Minor points:<br><br>Line 41-42. "Cellular resolution was achieved by enhanced tissue contrasting with phosphotunstic acid." The resolution of the scans is very good but not yet at the cellular level. Better to provide the actual voxel sizes here or amend "cellular".<br><br>Thank you for this comment, we have modified the sentence to tone down the claim on resolution.<br><br>Line 42/main text. Phosphotungstic is misspelled in several places throughout the manuscript.<br><br>We sincerely apologize for these grammatical mistakes and thank the reviewer for pointing them out. We have carefully checked the manuscript and corrected the errors.<br><br>Lines 68-69. "Mouse, chicken, clawed frogs, and zebrafish are among the most commonly used species in developmental biology". Add "vertebrate" before "species in developmental biology"<br><br>We have clarified that these are all vertebrate species.<br><br>Lines 74-77. This sentence needs adjustment. There is a constantly growing number of evo-devo labs exploiting sea lamprey and catshark models. The advances of genome editing with CRISPR/Cas9 has allowed several labs to perform genome editing in lamprey and many studies on catshark gene expression, cell tracing etc.<br>We thank the Reviewer for this comment and agree that there has been substantial progress in recent years, including the adoption of techniques such as CRISPR/Cas9-mediated genome editing in lampreys and a growing number of studies on gene expression, lineage (dye) tracing, and developmental processes in catsharks. We also agree that the number of laboratories working with these organisms is steadily increasing.<br><br>The intent of the sentence in lines 74-77, however, was not to suggest that lampreys and catsharks are rarely used or that methodological advances have not occurred, but rather to emphasize that, despite this progress, studies on cyclostomes and chondrichthyans still represent a comparatively small proportion of developmental and evo-devo research when viewed alongside the extensive body of work on osteichthyan models. To avoid any unintended implication that research activity in these systems is limited, we have revised the wording of the sentence to explicitly acknowledge the recent expansion of work in lampreys and catsharks while retaining the original point regarding their relative underrepresentation.<br><br>“Notable progress in breeding and maintaining jawless vertebrates (cyclostomes – lamprey and hagfish) [18], has allowed their broader inclusion in modern evo-devo [19–21]. In addition, cartilaginous fishes (Chondrichthyes) such as elephant sharks (Callorhinchus milii) and bamboo sharks (Chiloscyllium sp.) are also emerging as key research species [22–24]. However, the vast majority of vertebrate evo-devo research remains mostly restricted to representatives of Osteichthyes, leaving cyclostomes and Chondrichthyes comparatively underrepresented.”<br><br>Lines 93-94. Disagree to some extent, as the non-accessible one cell stage embryos makes genome editing very challenging in chondrichthyan species.<br><br>We agree and have now clarified that genome editing remains challenging in Chondrichthyes.

Lines 95-96. "by immunofluorescence and in situ hybridization" change to "combined with immunofluorescence and in situ hybridization"

We have changed the wording following the Reviewer’s suggestion.

Line 98. "remains challenges" change to " remains challenging"

We thank the reviewer for noting this grammatical error, we have corrected it.

Line 99-100. ' as well as the lack of high-quality genome assemblies, which hinder the design of in situ hybridization probes". Amend this phrase. With the projects like Vertebrate Genomes Project, the number of sequenced vertebrate genomes and their quality has increased dramatically. There are also numerous transcriptome datasets deposited in the GenBank even when the genome assembly is not complete.

We have removed the sentence following the Reviewer’s suggestion.

Line 103-104. "…we explored the use of X-ray-based methods, which allow imaging of thick samples at the centimeter scale." There are published studies applying X-ray synchrotron micro-tomography to catshark embryos. Add a citation for the recent study using synchrotron X-ray and iodine contrast on catshark late embryo (Mayeur et al 2024 MolBiolEvol).

We have added the reference following the Reviewer’s suggestion.

Line 108-109. "The first one is its inability to effectively differentiate soft tissues due to insufficient differences in their X-ray attenuation coefficients." By placing the sample at different distances from the objective and applying propagation phase contrast, thick soft tissues can be imaged and soft tissues can be differentiated, this is actively used in Human Organ Atlas Project by applying hierarchical phase-contrast synchrotron tomography (Walsh et al 2021 Nature Methods).

We thank the Reviewer for this important clarification and agree that propagation-based phase-contrast synchrotron tomography can enable visualization and differentiation of soft tissues, including in relatively thick specimens, as demonstrated for example in hierarchical phase-contrast approaches such as those used in the Human Organ Atlas Project (Walsh et al., 2021).

The statement in Lines 108–109 (now 113-114), however, refers specifically to conventional absorption-based µCT, for which limited intrinsic soft-tissue contrast remains a general constraint and is one of the reasons contrast agents are widely used. While phase-contrast methods can in principle be applied to unstained embryonic material, their effectiveness depends strongly on imaging geometry, beam coherence, and the intrinsic density gradients within the specimen. In small embryonic tissues, where density differences between adjacent structures can be subtle, unstained phase-contrast imaging does not always provide sufficient anatomical discrimination for the types of analyses performed here, and staining approaches remain common practice [9–11].

To avoid any misunderstanding, we have revised the sentence to clarify that this limitation applies primarily to conventional absorption-based µCT and to acknowledge that phase-contrast methods can overcome this constraint under appropriate conditions, while keeping the introduction concise for a general readership.

Line 111-112. Some contrasting agents are also absorbed by hard tissues, but not PTA, this is important to mention here.

We have now stated that different contrasting agents exhibit distinct absorptions, as reflected in the resulting contrasted images, and explicitly mentioned that PTA is not absorbed by hard tissues (please see answer to point 3.2).

Line 122. "(space and time)" change to "(volume and time)"

Powered by Editorial Manager® and ProduXion Manager® from Aries Systems Corporation

We have changed the wording following the Reviewer's suggestion.

Line 123. "often large" change to "non-transparent and thick"

We have changed the wording following the Reviewer's suggestion.

Line 127. "The provided dataset with superior quality and excellent contrast will benefit…" Amend this phrase. The quality is based on resolution, the resolution above micron is not a superior resolution. A dataset at submicron resolution would produce a better quality dataset.
Contrast quality is not excellent, as it is dependent on individual sample preparation and PTA is also known to be less suitable contrast agent for some of the skeletal tissues. Contrast quality could also have been improved by changing propagation distances of imaged sample to detector, depending on the sample sizes.

We have toned down this statement.

"The provided dataset will benefit researchers interested in Chondrichthyes embryology and comparative evo-devo studies,"

Line 138. State the concentration of tricaine for overdose treatment

We have added the tricaine concentration used for the overdose treatment.

Line 141. "increasing ethanol … steps" change to "increasing ethanol …concentrations in PBS"

We have changed the wording following the Reviewer's suggestion.

Line 142. "the long ethanol steps" change to " the long incubation in ethanol"

We have changed the wording following the Reviewer's suggestion.

Line 148. "Till scanning" change to "Until scanning"

We have changed the wording following the Reviewer's suggestion.

Line 152. Provide details of resin product number and manufacturer.

We apologize for the lack of information on the UV-light-sensitive resin used to seal the pipette tips. We have now incorporated this information into the text.

Line 160-173. Provide the details of the technical equipment i.e. camera, lens, scintillator used.

We have added the requested information to the main text.

Was propagation distance i.e. sample to detector distance changed between smaller and larger specimen to maximize a phase contrast effect?

Since all the samples were well contrasted and could be clearly visualized, we did not employ the phase-contrast scanning mode but instead used attenuation contrast. We first scanned a small and a large sample at the same sample-to-detector distance and observed that the quality and contrast of the resulting images were good for both. Hence, the sample-to-detector distance was not changed between smaller and larger specimens, and all were scanned with the same distance (80mm).

Describe if the beam was filtered and how.

The monochromatic beam was filtered using a double Christal monochromator with 1,1,1-silicon crystals to set a final photon energy of 20keV.

It is important to write if imaging was done over 360 degrees, and in a half or full acquisition mode.

Scanning was performed on full acquisition mode over 180° and an angular step of 0.059°. We have added this information to the main text.

Line 282. "manipulate" change to "segment"

We have changed the wording following the Reviewer's suggestion.

Line 284. Add Mimics, and Dragonfly to the list of software.

We thank the reviewer for pointing these two additional software options and have added them to the list.

Figure 1A. What is dark/light color of eggshells linked to? This should be explained in the Figure legend.

We appreciate this very interesting question raised by the Reviewer. The small-spotted catshark eggshells are known to display a range of coloration from light beige to dark brown, as shown in Fig. 1A [12,13]. However, the underlying causes of this phenotypic variation remain unknown. In our breeding tanks, all adult catshark females were maintained under the same stable conditions (temperature, salinity, oxygenation, diet). It has been suggested that each female lays eggs of the same color [12]. Hence, eggshell coloration is likely linked to genetic factors rather than environmental ones. However, proving this hypothesis would require additional investigation. Please note that eggshell color does not affect embryo development in any way [13]. We have incorporated this information in the Fig. 1 legend (see lines 618-621).

"Small-spotted catshark eggshells display a broad range of coloration from light beige to dark brown [89,90]. The underlying causes of this phenotypic variation remain unknown, although genetic factors have been suggested as contributing influences [89]. Importantly, eggshell pigmentation does not affect embryo development [90]."

Figure 1C. Is St15 embryo on the right mounted with rostral part down or up, add this information to the Figure legend.

We apologize for the lack of clarity regarding the embryonic axis and orientation of the embryos in the figure, we have clarified them in the figure as well as in the figure legends. For the Reviewer's information, the St.15 embryo on the right is positioned dorsally with the rostral (anterior) part up.

Figure 2-4. Label corresponding developmental stages on the Figure, not only in the Figure legend.

We thank the reviewer for this suggestion and have now incorporated the corresponding embryonic stages into all the figures.

Supplementary Table 2. Provide embryo length and/or somite number for each sample.

We have incorporated the requested information on embryo length and somite number in Supplementary Table 2 (please see answer to point 6).

References

1. Lesciotto KM, Perrine SMM, Kawasaki M, Stecko T, Ryan TM, Kawasaki K, et al.. Phosphotungstic acid enhanced microCT: optimized protocols for embryonic and early postnatal mice. Dev Dyn. 2020; doi: 10.1002/dvdy.136.
2. Pétré M, Balcaen T, Schneidewind P, Mazy L, Pyka G, Fehervary H, et al.. Screening staining agents for contrast-enhanced microCT of vascular tissues: Assessing the effect on microstructural and mechanical properties. Tomography of Materials and Structures. 2024; doi: 10.1016/j.tmater.2024.100038.
3. Balint R, Lowe T, Shearer T. Optimal Contrast Agent Staining of Ligaments and Tendons for X-Ray Computed Tomography. PLOS ONE. Public Library of Science;

	2016; doi: 10.1371/journal.pone.0153552.<br>4. Schmied C, Jambor HK. Effective image visualization for publications – a workflow using open access tools and concepts. F1000Research;<br>5. Matula J, Polakova V, Salplachta J, Tesarova M, Zikmund T, Kaucka M, et al.. Resolving complex cartilage structures in developmental biology via deep learning-based automatic segmentation of X-ray computed microtomography images. Sci Rep. Nature Publishing Group; 2022; doi: 10.1038/s41598-022-12329-8.<br>6. Kaucka M, Zikmund T, Tesarova M, Gyllborg D, Hellander A, Jaros J, et al.. Oriented clonal cell dynamics enables accurate growth and shaping of vertebrate cartilage. Bronner M, editor. eLife. eLife Sciences Publications, Ltd; 2017; doi: 10.7554/eLife.25902.<br>7. Kaucka M, Petersen J, Tesarova M, Szarowska B, Kastriti ME, Xie M, et al.. Signals from the brain and olfactory epithelium control shaping of the mammalian nasal capsule cartilage. White RM, editor. eLife. eLife Sciences Publications, Ltd; 2018; doi: 10.7554/eLife.34465.<br>8. Sunadome K, Erickson AG, Kah D, Fabry B, Adori C, Kameneva P, et al.. Directionality of developing skeletal muscles is set by mechanical forces. Nat Commun. Nature Publishing Group; 2023; doi: 10.1038/s41467-023-38647-7.<br>9. Matula J, Tesarova M, Zikmund T, Kaucka M, Adameyko I, Kaiser J. X-ray microtomography–based atlas of mouse cranial development. GigaScience. 2021; doi: 10.1093/gigascience/giab012.<br>10. Laznovsky J, Kavkova M, Helena Reis A, Robovska-Havelkova P, Maia LA, Krivanek J, et al.. Unveiling vertebrate development dynamics in frog Xenopus laevis using micro-CT imaging. GigaScience. 2024; doi: 10.1093/gigascience/giae037.<br>11. Metscher BD. MicroCT for developmental biology: A versatile tool for high-contrast 3D imaging at histological resolutions. Developmental Dynamics. 2009; doi: 10.1002/dvdy.21857.<br>12. Mellinger J. Egg-case diversity among dogfish, Scyliorhinus canicula (L.): a study of egg laying rate and nidamental gland secretory activity. Journal of Fish Biology. 1983; doi: 10.1111/j.1095-8649.1983.tb04728.x.<br>13. Koch A-K, Grunow B, Moritz T. Recommendations for scientific fish husbandry: Scyliorhinus canicula (Carcharhiniformes, Scyliorhinidae) – a model species for sharks. Bulletin of Fish Biology. 2025; doi: doi: 10.53188/BFB0012.
**Additional Information:**	
**Question**	**Response**
Are you submitting this manuscript to a special series or article collection?	No
**Experimental design and statistics**<br><br>Full details of the experimental design and statistical methods used should be given in the Methods section, as detailed in our Minimum Standards Reporting Checklist. Information essential to interpreting the data presented should be made available in the figure legends.<br><br>Have you included all the information requested in your manuscript?	No
If not, please give reasons for any omissions below.	No statistical analyses were performed in this study.

as follow-up to "**Experimental design and statistics**<br><br>Full details of the experimental design and statistical methods used should be given in the Methods section, as detailed in our Minimum Standards Reporting Checklist. Information essential to interpreting the data presented should be made available in the figure legends.<br><br>Have you included all the information requested in your manuscript?<br><br>"	
**Resources**<br><br>A description of all resources used, including antibodies, cell lines, animals and software tools, with enough information to allow them to be uniquely identified, should be included in the Methods section. Authors are strongly encouraged to cite Research Resource Identifiers (RRIDs) for antibodies, model organisms and tools, where possible.<br><br>Have you included the information requested as detailed in our Minimum Standards Reporting Checklist?	Yes
**Availability of data and materials**<br><br>All datasets and code on which the conclusions of the paper rely must be either included in your submission or deposited in publicly available repositories (where available and ethically appropriate), referencing such data using a unique identifier in the references and in the "Availability of Data and Materials" section of your manuscript.<br><br>Have you have met the above requirement as detailed in our Minimum	Yes

Powered by Editorial Manager® and ProduXion Manager® from Aries Systems Corporation

Standards Reporting Checklist?	
GigaScience has policies and guidelines in place for the use of generative AI-writing tools such as ChatGPT. If you have used such writing tools to assist with writing the manuscript this must be declared and cited in the text. Authors should not list AI-writing tools and other AI-assisted technologies as an author or co-author and should acknowledge that they are fully responsible for text generated or refined by AI-writing tools.<p><br><br>A summary of use (particularly in the introduction or among methods) needs to be included at the end of the paper, and the outputs should also be included as a supplementary file hosted in GigaDB or other open repositories. Please read our guidelines for more information. <p><br><br>By submitting to GigaScience, you are aware of the journal's AI-writing tools policy, and if you have declared use of such tools below, you have acknowledged this where appropriate in your manuscript and have made a summary of use and outputs available. <p><br><b>AI-assisted writing tools have been used in the preparation of this manuscript?	No

Powered by Editorial Manager® and ProduXion Manager® from Aries Systems Corporation

Manuscript
Click here to access/download;Manuscript;Escamilla_MainText_GigaD250031

Synchrotron radiation micro-computed tomography of the small-spotted catshark embryonic development (Chondrichthyes: *Scyliorhinus canicula*)

Authors

Elio Escamilla-Vega[1], Ann-Katrin Koch[2,3], Louk W. G. Seton[1], Andrea P. Murillo-Rincón[1], Stella Kyomen[1], Jörg U. Hammel[4], Timo Moritz[2,5], Markéta Kaucká[1*]

[1]Max Planck Institute for Evolutionary Biology, August-Thienemann-Str. 2, 24306 Plön, Germany

[2]Ocean Museum Germany, Katharinenberg 14–20, 18439 Stralsund, Germany

[3]Institute of Biosciences, University of Rostock, Albert-Einstein-Str. 3, 18059 Rostock, Germany

[4]Institute of Materials Physics, Helmholtz-Zentrum Hereon, Max-Planck-Str. 1, 21502 Geesthacht, Germany

[5]Leibniz Institute for the Analysis of Biodiversity Change, Martin-Luther-King-Platz 3, D-20146 Hamburg, Germany

e-mail address and ORCID of all authors:

Elio Escamilla-Vega [0009-0005-6106-0802]: escamilla@evolbio.mpg.de

Ann-Katrin Koch [0000-0001-8810-9171]: Ann-Katrin.Koch@meeresmuseum.de

Louk W.G. Seton [0009-0000-6946-7686]: seton@evolbio.mpg.de

Andrea P. Murillo-Rincón [0009-0003-7532-3736]: amurillo@evolbio.mpg.de

Stella Kyomen [0000-0003-0105-4636]: kyomen@evolbio.mpg.de

Jörg U. Hammel [0000-0002-6744-6811]: joerg.hammel@hereon.de

Timo Moritz [0000-0003-1281-7432]: T.Moritz@leibniz-lib.de

Markéta Kaucká [0000-0002-8781-9769]: kaucka@evolbio.mpg.de

***Corresponding author**

Markéta Kaucká [0000-0002-8781-9769]: kaucka@evolbio.mpg.de

Abstract

Background. Sharks occupy a key position on vertebrate phylogeny, making them essential for understanding the early origins of jawed vertebrates (gnathostomes) and the functional adaptation of vertebrate traits like jaws or complex sensory systems. As such, sharks are important model organisms in evolutionary developmental biology (evo-devo), but sparse data and limited availability of samples hinder their inclusion in contemporary evo-devo research. The knowledge of their distinctive morphology will not only shed light on the anatomical architecture and physiology of basal living gnathostomes but also reveal the evolutionary divergence of developmental processes that establish the foundational vertebrate blueprint.

Findings. We performed synchrotron radiation micro-computed tomography (SRμCT) scanning of the small-spotted catshark (*Scyliorhinus canicula*) embryonic development, spanning from gastrulation (stage 12) to late-organogenesis (stage 31), enhanced by tissue contrasting with phosphotungstic acid. We obtained 36 whole-embryo scans that encompass the formation of key embryonic structures, such as sensory organs, fins, muscles and skeletal elements. The achieved resolution allows for segmentation of all tissue types and both internal and external structures.

Conclusions. We present a comprehensive dataset of 4D high-resolution SRμCT of the small-spotted catshark embryonic development. The dataset spans consecutive embryonic stages, allowing the reconstruction and morphometric analyses of tissues, organs, and structures, along with the tracking of their development. The deposited data are publicly available, and provide a valuable resource for

comparative research, additionally allowing the identification of conserved and derived developmental processes and features and understanding the evolution of vertebrates.

Issue section: Data Note

Keywords: synchrotron radiation micro-computed tomography, X-rays, tissue contrast, cartilaginous fish, Chondrichthyes, small-spotted catshark, *Scyliorhinus canicula*, shark ontogeny, evo-devo.

Background

Vertebrate development represents a series of tightly regulated steps that orchestrate the formation of all body structures and their functional integration. While many fundamental processes, such as axis formation, segmentation, and organ primordia patterning, are shared across taxa, there are also significant differences that result in the remarkable morphological diversity of vertebrates [1–4]. The differences in embryogenesis reflect both phylogenetic divergence and species-specific adaptations, such as ecological and reproductive pressures [5–7]. Comparative embryology across a broad range of vertebrates is therefore essential for disentangling ancestral traits from lineage-specific innovations and for understanding the evolution of developmental processes that generate morphological diversity.

In the past century, a limited repertoire of model vertebrate organisms has been used for comparative embryology research. These studies provided a solid foundation for our understanding of vertebrate development, and identified events, structures and embryonic stages where the evolutionary divergence of developmental programs arises. Mouse, chicken, clawed frogs, and zebrafish are among the most commonly used vertebrate species in developmental biology [8,9]. More recently, the taxon sampling has expanded with the introduction of emerging experimental organisms like bats [10,11], ostriches [12,13],

lizards [14,15] and non-teleost fishes [16,17], which has enabled comparative embryology to provide valuable insights into the mechanisms underlying the evolution of vertebrate developmental programs. All these species belong to the same superclass of bony vertebrates (Osteichthyes), thus not covering the entire vertebrate subphylum. Notable progress in breeding and maintaining jawless vertebrates (cyclostomes – lamprey and hagfish) [18], has allowed their broader inclusion in modern evo-devo [19–21]. In addition, cartilaginous fishes (Chondrichthyes) such as elephant sharks (*Callorhinchus milii*) and bamboo sharks (*Chiloscyllium* sp.) are also emerging as key research species [22–24]. However, the vast majority of vertebrate evo-devo research remains mostly restricted to representatives of Osteichthyes, leaving cyclostomes and Chondrichthyes comparatively underrepresented.

Chondrichthyes are the sister group of Osteichthyes and represent one of the two lineages of living jawed vertebrates (gnathostomes). This morphologically diverse group of animals, which diverged from a common ancestor about 420 million years ago, is divided into two classes: Elasmobranchii (sharks, rays, skates and sawfish) and Holocephali (chimaeras) [25]. Chondrichthyans are primarily known for having a cartilaginous endoskeleton throughout their lives, and the loss of perichondral, endochondral, and most dermal bones [26–29]. These traits render them important models for the research of skeletal tissue evolution. Their unique features and phylogenetic position render Chondrichthyes a valuable taxon in vertebrate comparative evo-devo research [30–33]. However, a broader inclusion of Chondrichthyes in contemporary embryological research remains limited due to several inherent challenges. The difficulty in obtaining sufficient numbers of embryos resulting from reduced mating in captivity [34–36], restricted habitats (primarily chimaeras), seasonal breeding with low fecundity [37], reproductive modes that often require the sacrifice of adult females [38,39], high extinction risks [40], long generation times (approximately 175 days in the small-spotted catshark) [41], and the challenges associated with maintaining these species in aquarium settings, hinder their establishment as laboratory model system. Nevertheless, despite these limitations, two oviparous species have emerged as promising model organisms in evo-devo research: the little skate (*Leucoraja erinacea*) and the small-spotted catshark

(*Scyliorhinus canicula*) [36]. The small-spotted catshark is an abundant, non-endangered species commonly kept in aquaria, capable of mating in captivity and laying eggs throughout most of the year. Moreover, this shark species is well-suited for modern genomic approaches, supported by a chromosomal-level genome assembly and numerous transcriptomic resources [42–45], although genome editing remains a major challenge not only in the small-spotted catshark but generally across chondrichthyans.

Non-destructive three-dimensional imaging techniques, such as confocal laser scanning microscopy combined with immunofluorescence and *in situ* hybridization, have been used to visualize and follow the development of specific anatomical structures [46–49]. However, applying these techniques to non-model organisms remains challenging, for instance, due to the limited availability of specific and effective antibodies for immunostaining. Additionally, although confocal microscopy remains as one of the most popular and widespread 3D imaging techniques, it is limited by the need for fluorescent labelling of the structures of interest, and insufficient light penetration becomes problematic in larger samples. To overcome these limitations and image larger samples, we explored the use of X-ray-based methods, which allow imaging of thick samples at the centimeter scale [42].

Micro-computed tomography (µCT) is a frequently used approach in developmental biology [50–52]. Samples are irradiated with X-rays from multiple angles, obtaining 2D projections which are later computationally reconstructed into 3D models. Even though absorption-based µCT provides an excellent way to obtain high-resolution 3D data, it also has two main limitations. The first one is its inability to effectively differentiate soft tissues due to insufficient differences in their X-ray attenuation coefficients, (phase-contrast imaging may overcome this constraint under appropriate conditions). As a result, only dense structures like bones, teeth and scales are readily visible in the reconstructed tomographic images [53,54]. This limitation can be overcome by using contrasting agents, which are differentially absorbed by soft tissues, allowing their visualization in the final images [55]. Various contrast-enhancing agents have been tested, including iodine [51,56–58], ruthenium red [59], and phosphotungstic acid (PTA) [50,60,61], each with distinct tissue-specific absorption rates and, thus, contrasting abilities (e.g., PTA

does not stain cartilage) [62–65]. The second, unavoidable limitation arises from the physical properties of conventional laboratory-based µCT systems. These conventional scanners are prone to beam hardening artifacts, low signal-to-noise ratios, and resolution constraints, particularly when imaging smaller samples, which can make fine structures indistinguishable in the final images [66,67]. In synchrotron facilities, the high-energy nearly-parallel monochromatic X-ray beam makes it possible to obtain high spatial resolution (i.e. micron scale) images of macroscopic samples (centimeter range) with improved quality and reduced imaging times [68,69]. Synchrotron radiation micro-computed tomography (SRµCT) combined with iodine-based contrast enhancement was previously employed to reconstruct and characterize the sensory organs in a pre-hatching small-spotted catshark embryo [42], highlighting the potential of this imaging technology to study chondrichthyan development in great detail.

Here, we took advantage of SRµCT combined with PTA-contrasting to generate a high-resolution 4D (volume and time) atlas of the small-spotted catshark development and extend the available information from pre-hatching stages [42]. SRµCT is particularly valuable for studying shark embryos, which are non-transparent and thick and difficult to image using optical techniques. This strategy allowed the manual segmentation of a wide range of embryonic structures, and in turn, the reconstruction of the developmental progression that shape the shark body plan and anatomical features. We provide the raw reconstructed tomographic slices together with the pre-processed files. The provided dataset will benefit researchers interested in Chondrichthyes embryology and comparative evo-devo studies, addressing a wide spectrum of research questions, without requiring access to new specimens or museum-preserved samples.

Methods

Small-spotted catshark sample preparation

Fertilized small-spotted catshark eggs were obtained from Ozeaneum (Stralsund, Germany) and Sea Life Berlin (Berlin, Germany) (Fig. 1A), opened using sharp dissecting scissors and the whole content of the

eggs were placed in glass Petri dishes with oxygenated sea water (Fig. 1B). The embryos were then carefully separated from the yolk under a stereomicroscope, staged according to Ballard [41], euthanized by a tricaine (Ethyl-3-aminobenzoat-methansulfonat, Merck, E10521) overdose (0.04% tricaine in sea water) [70,71], and fixed in freshly prepared 4% paraformaldehyde (PFA) in 1X Dulbecco's Phosphate Buffered Saline (PBS – Sigma D5652) for 24h at 4°C with gentle rotation. Following fixation, embryos were washed with PBS and subsequently dehydrated in increasing ethanol (Fisher Chemical E/0650DF/17) concentrations in PBS (30%, 50%, 75% and 90%) for 24h each on slow rotation. The slow rotation together with the long incubation in ethanol ensures an evenly dehydration while minimizing tissue shrinking and preserving of the morphological features. Embryos were then incubated in 1.5% PTA (Sigma P4006) in 90% methanol (Fisher Chemical M/4056/17) to enhance the contrast and visualize soft tissues [50,60,61,63,64]. The PTA contrasting solution was replaced twice a week. The staining times for each developmental stage are detailed in Supplementary Table 1. Once the embryos were saturated with contrasting solution, they were washed twice in 100% ethanol for 24h to remove any PTA excess and stored in 100% ethanol until scanning.

SRμCT image acquisition

PTA-contrasted small-spotted catshark embryos were placed inside bottom-sealed plastic pipette tips filled with 100% ethanol (Fig. 1C). The pipette tips were bottom-sealed using UV-light sensitive resin (UVR-100, MOCOBO) and their volume was chosen with respect to the size of the embryo (Fisher Scientific - 10μL 0030073371, 200μL 0030073436, 1000μL 0030000927), with larger embryos placed in larger tips and vice versa. The conical shape of the pipette tips keeps the embryos in place and prevents movements during scanning. Once the embryos were fixed inside the pipette tips, they were carefully aligned as straight as possible using fine forceps in the center to avoid scanning artifacts. Subsequently,

the tips were top-sealed using a hot glue gun to avoid drying of the samples during the scans, and glued to a standardized sample holder that fits in the rotation stage (Fig. 1C).

Attenuation-contrast SRµCT measurements on full acquisition mode were acquired at the Imaging Beamline P05 of the storage ring PETRA III (Deutsches Elektronen Synchrotron–DESY, Hamburg, Germany) operated by the Helmholtz-Zentrum Geesthacht [72] (Fig. 1D). A double Christal monochromator with 1,1,1-silicon crystals was used to set a photon energy of 20keV. All embryos were imaged with the same photon energy (20keV) and a sample-to-detector distance of 80mm. 3001 projections equally spaced between 0° and 180° were obtained for each tomographic scan (angular step of 0.059°) using a custom-developed 20 MP CMOS camara system [73], custom-made lenses (Präzisions Optik Gera, Germany), and a 100µm CdWo4 scintillator. To optimize scanning time while obtaining the highest possible resolution for each sample, embryos were divided in two groups depending on their size and imaged with a different field of view (FOV) and exposure time. Embryos ranging from St.12-25 were imaged with a FOV of 3.29mm x 2.47mm, with an exposure time of 280ms. Embryos ranging from St.26-31 were imaged with a FOV of 6.57mm x 2.70mm, with an exposure time of 80ms. Multiscan vertical tiling was applied depending on embryo length to cover the whole specimen, with 16 tiles for the largest embryo (St.31). The scanning time for one tile was 16.4min and 4.7min for each FOV, respectively. Interestingly, the interaction between the X-ray radiation and the PTA contrasting agent causes the samples to turn temporarily blue after scanning (Fig. 1E). Detailed information regarding the SRµCT scanning parameters per sample can be found in Supplementary Table 2.

Image reconstruction and pre-processing

Tomographic reconstruction of the 2D projections into 3D volumes was performed using a classical filtered back projection (FBP) with a two-fold binning based on a custom reconstruction pipeline implemented in MATLAB and the Astra Toolbox [74–77] (Fig. 1F). The reconstruction pipeline is

deposited in GitHub [78]. The resulting reconstructed 3D images were saved as tiff. image stacks with an effective isotropic voxel size of 1.28µm and 2.57µm for each FOV, respectively. For samples requiring multiple vertical scans, the resulting tiles were stitched together using the same custom-designed algorithm. Manual adjustment of the stitched tiles was required to correct for minor misalignment artifacts observed in some samples. The final reconstructed and stitched files ranged between 46.8GB and 381GB. In total, we generated 5.04TB of 3D data for further analysis.

Due to the large size of the reconstructed files, additional pre-processing was required before image analysis or manual segmentations could be performed. The 2D reconstructed stacks were first loaded into ImageJ/Fiji 2.9.0 [79] and cropped to retain only the embryos while removing the surrounding empty space. Afterwards, the brightness and contrast were adjusted using the Brightness/Contrast function to refine the range of color values and enhance visualization of the embryonic structures [80] (Fig. 1F and Supplementary Fig. 1). Finally, images were converted from 32-bit into 8-bit format (Fig. 1F). By performing this series of transformations, a reconstructed stack of 46.8GB reduced its size to approximately 1.3GB. While the pre-processing steps were typically sufficient to reduce the size of most files, larger samples (St.25-31) required additional resampling to further reduce loading times and ensure efficient segmentations. Although resampling decreased the number of pixels and, thus, reduced image quality, the obtained pre-processed images remained of high quality and resolution.

Manual segmentation of embryonic structures

Based on the pre-processed reconstructed SRµCT images, the different embryonic structures of the developing small-spotted catshark embryos were manually segmented using the interpolation and wrap functions from Avizo3D Pro Software (ThermoFisher Scientific, Konrad-Zuse-Zentrum, Berlin, Germany) (Fig. 2). With the interpolation function, the experienced operator manually segmented every third slice on the same orthogonal projection and the rest was automatically calculated by linear

interpolation between the adjacent manually segmented slices. The interpolation tool was particularly useful when changes from slice-to-slice are small and progressive, like for the brain or ectodermal placodes. With the wrap selection function, the operator first created a scaffold by manually segmenting slices in the three orthogonal projections (XY, XZ, YZ) and then, an algorithm automatically computed the remaining slices. The wrap tool was especially helpful for structures with more complex three-dimensional shapes, like the somites. These tools considerably helped reduce the workload and increased the segmentation speed without impacting the accuracy [81], particularly important for large datasets such as the one provided here. Nonetheless, manual fine-tuning was necessary to ensure the accuracy of the segmentation in complex and fine structures. The manual segmentation of the individual embryonic elements took 1-5 days per sample for the smaller embryos (St.12-20) and up to 2 weeks for larger ones with more complex anatomy (St.21-31). Smoothing of 3D renderings was performed to reduce the staircase artifacts that appeared due to the manual segmentations of the structures in 2D slices. A slight tissue shrinkage was observed due to the overall tissue dehydration and PTA-contrasting enhancement (1.5% PTA in 90% methanol), which represents a known, unavoidable technical limitation of enhanced soft tissue contrast [65,82,83]. This effect was particularly evident in ectodermal structures starting at St.25 (Supplementary Fig. 2). However, despite the slight tissue shrinkage, the overall morphology and relative anatomical position of the different embryonic structures remain intact. Moreover, the relative order in which individual structures emerge along the developmental timeline are not changed, important for studies on developmental heterochrony.

Validation of SRµCT data by confocal microscopy

The segmentation of different embryonic structures was performed based on morphological landmarks, contrast differences between adjacent tissues, and publicly available information on Chondrichthyes and vertebrate development. The segmented 3D reconstructions of the developing structures can be visualized

simultaneously in the same model, which helps understand the spatial relationships between distinct tissues and organs (Fig. 3A). The segmented 3D models can be directly compared between consecutive developmental stages to understand the complex morphogenetic processes during embryogenesis together with organ growth and shaping. This approach can be applied to virtually any of the developing structures. Moreover, the obtained 3D models can be additionally integrated with other popular non-destructive 3D techniques such as confocal laser scanning microscopy with the use of immunofluorescence or HCR *in situ* hybridization (Fig. 3B, C). These two methods are essential tools in modern evo-devo research, enabling precise labelling of distinct cellular populations and anatomical structures within the developing embryo based on protein location and differential gene expression, respectively [84]. By combining the high-resolution SRµCT morphological data with fluorescence spatial gene expression mapping, we are able to establish a powerful framework to link genetic programs with tissue architecture and organismal morphology.

Briefly, following embryo collection and fixation, small-spotted catshark embryos were washed with PBS and subsequently dehydrated in increasing methanol (Fisher Chemical M/4056/17) steps (25%, 50%, 75% and 100%) diluted in 0.1% PBST for 15min each on slow rotation on ice. Subsequently, they were incubated in methanol for 1h on slow rotation at room temperature and stored in methanol at -20°C till further use. To reduce autofluorescence and improve the fluorescence signal, samples were bleached by incubation in Dent's Bleach solution (1 volume Vaprox® – Steris PB006EUR; 2 volumes Dent's Fix solution – 80% methanol, 20% DMSO – Roth A994.1) overnight on slow rotation at 4°C. Afterwards, samples were washed 3x10min in methanol and incubated overnight at 4°C in Dent's Fix solution.

Immunofluorescence. Bleached embryos were rinsed 3 times in 0.1% PBST and subsequently washed 3x20min in 0.1% PBST at 4°C to remove any traces of DMSO and methanol. Subsequently, they were incubated at room temperature for 5 days in primary antibody diluted in blocking solution composed of 20% DMSO and 5% donkey serum (Interchim UP77719A-K) in PBS. The following antibody was used to label the peripheral nervous system and immature neurons of the central nervous system: mouse anti-

Tuj1 (Promega G7121; 1:500). Following primary antibody incubation, samples were rinsed 3 times in 0.1% PBST, washed 3x20min in 0.1% PBST and incubated in secondary antibody at RT for 3 days diluted in blocking solution. The secondary antibody used was produced in donkey and conjugated with Alexa Fluor 647 (ThermoFisher A31571; 1:1000). Nuclear stain was performed by incubating embryos overnight at 4°C in 1X DAPI (4′,6-diamidino-2-phenylindole; ThermoFisher D21490) in PBST. Finally, samples were rinsed 3 times in 0.1% PBST, washed 3x20min in 0.1% PBST and cleared using BABB [85]. Whole-mount imaging was performed using glass bottom imaging dishes (Eppendorf 0030740017) in a Zeiss LSM980 with Airyscan2 confocal microscope with the Plan-Apochromat 10x/0.45 M27 (Zeiss 420640-9900) objective. Final confocal images were processed and exported from ZEN 3.9 software.

HCR *in situ* hybridization. Bleached embryos were rehydrated in decreasing methanol series 15min each (75%, 50%, 25%, 0% - in 0.1% PBST) at 4°C on slow rotation, post-fixed 15min at room temperature in 4% PFA and pre-hybridized in 30% probe hybridization buffer for 30min at 37°C. Once samples sank to the bottom of the tube, they considered equilibrated in probe hybridization buffer and subsequently incubated overnight at 37°C in 2pmol of HCR probes diluted in 30% probe hybridization buffer in a thermomixer (Eppendorf 5355) with gentle shaking (450rpm). The following morning, samples were washed 4x15min in 30% probe wash buffer at 37°C, 4x15min in 0.1% 5X SSC-Tween (SSCT) at room temperature (20X SSC; Fisher bioreagents BP1325-4) and pre-amplified in HCR amplification buffer for 30min. Once equilibrated, samples were incubated overnight at room temperature in 30pmol of fluorescent-labelled hairpins diluted in amplification buffer, in the dark [84,86]. On the following morning, samples were washed 4x15min in 5X SSCT to remove excess hairpins and incubated overnight at 4°C in 1X DAPI in 5X SSCT to counterstain the nuclei. Finally, samples were washed 4x15min in 5X SSCT, cleared using BABB and imaged using a Zeiss LSM980 with Airyscan2 confocal microscope.

Reuse potential

Beyond the representative examples shown (Fig. 2 and 3), there are numerous opportunities to employ this dataset in a wide spectrum of studies, allowing to analyse the formation of virtually any identifiable embryonic structure: cranial placodes (adenohypophyseal, olfactory, lens, trigeminal, profundal, lateral line, otic and epibranchial), sensory organs, teeth, skin, skin denticles, head cavities, neural crest cells, facial mesenchyme, cartilage, vertebral column, skull, gills, pharyngeal arches, pharyngeal pouches, somites, muscles, central nervous system (forebrain, midbrain, hindbrain and spinal cord), peripheral nervous system (cranial ganglia, dorsal root ganglia, nerves), endocrine organs, kidneys, stomach, liver, gut, heart, blood vessels, fins (pectoral, pelvic, dorsal, anal, caudal), reproductive system, and many more (see examples of different structures from representative developmental stages in Fig. 4). This dataset captures the early morphogenetic events that lead to the formation of the general chondrichthyan body plan from the three main embryonic germ layers (endoderm, mesoderm, ectoderm), till later stages of organogenesis, when species-specific differences begin to emerge among embryos of distinct chondrichthyan species [87]. The analysis of consecutive developmental stages allows researchers to reconstruct and trace the embryonic origin of the developing anatomical features (Fig. 5), providing key insights into the developmental trajectories and formation of tissues and organs in Chondrichthyes. Moreover, during embryogenesis, tissues and organs arise and grow in a synchronous manner, establishing tight connections and integrating to form a functional organism. Such high-dimensional interactions are challenging to study by conventional 2D and 3D methods and require high-resolution non-destructive imaging techniques. This SRµCT dataset represents a comprehensive resource to resolve tissue architecture and the integration of embryonic structures in 3D space.

Small-spotted catshark embryos at the same developmental stage often exhibit slight morphological differences depending on their precise developmental timing. For instance, early St.26 embryos (approximately 37 days after deposition) have approximately 85 pairs of somites, while late St.26 embryos (approximately 42 days after deposition) have around 90 pairs of somites [41]. This within-stage variation highlights that embryonic development is a continuous process despite being conventionally subdivided

into discrete stages. The presented dataset can serve as a starting point for future studies of within-stage developmental differences since it provides scans of at least two independent embryos from St.17-29, allowing a more refined characterization of small-spotted catshark development (Supplementary Fig. 3).

Research on Chondrichthyes development is challenging due to the difficulties in obtaining fertilized eggs or embryonic material. The presented SRµCT dataset serves as a valuable and information-rich resource to investigate shark embryonic development and incorporate Chondrichthyes into modern comparative embryology studies, enabling a deeper understanding of the divergence and conservation of the developmental programs shaping vertebrate morphological diversity.

Data availability

The SRµCT dataset behind this manuscript is available in the Electron Microscopy Public Image Archive (EMPIAR, accession number: EMPIAR-12984). We provide the raw reconstructed SRµCT data as well as the pre-processed files. Because of the large size of the raw reconstructed files, we recommend using the pre-processed data instead. If using the raw reconstructed files, please note that small misalignments of the vertical tiles may appear due to stitching artifacts and will need to be manually corrected by the operator. All data analysis and segmentations were performed on a workstation equipped with the following hardware components: an NVIDIA Quadro P5000 graphics processing unit (GPU), an Intel Xeon W-2145 central processing unit (CPU), and 64GB of system memory (RAM). Based on our experience with this configuration, we recommend resampling the files to obtained a final working image stack of less than 2GB of size, or ideally less than 1GB when extensive segmentation is expected. However, file size limitations may vary depending on the specification of the workstation used. The dataset is presented as TIFF stacks of the corresponding tomographic slices. Each folder contains the information for one single embryo. The naming of files is as follows: ScCan_stage_replicate_32b.tiff for raw reconstructed files and ScCan_stage_replicate_8b.tiff for pre-processed files. Information about the

voxel size for each sample can be found in Supplementary Table 2. For the production, curation and analysis of this SRµCT dataset we used ImageJ/Fiji [79] and Avizo3D Pro Software. However, other software options are available to segment, visualize and analyze this dataset such as 3D Slicer [88], Amira (ThermoFisher Scientific), VG Studio MAX (Volume Graphics GmbH, Germany), Mimics (Materialise NV), Dragonfly 3D (Object Research Systems Inc., Comet group) or MeshLab [89]. All additional supporting data are available in the *GigaScience* repository, GigaDB [90]. The GigaDB platform is described in detail in Li et al. (2026) [91].

List of abbreviations

µCT: micro-computed tomography; µm: micrometer; cm: centimeter; CPU, central processing unit; evo-devo: evolutionary developmental biology; EMPIAR, Electron Microscopy Public Image Archive;FOV: field of view; h: hour; GB: gigabyte; GPU, graphics processing unit; HCR: hybridization chain reaction; min: minute; mm: millimeter; ms: millisecond; PBS: Dulbecco's Phosphate Buffered Saline; PFA: paraformaldehyde; PTA: phosphotungstic acid; RAM, random-access memory; St: stage; SRµCT: synchrotron radiation micro-computed tomography; TB: terabyte; UV: ultraviolet.

Declarations

All animal work was conducted following Directive 2010/63/EU, the German Animal Welfare Act (Tierschutzgesetz § 11) and in compliance with the Federation of European Laboratory Animal Science Associations' guidelines for the housing, handling and euthanasia of laboratory animals. The collection of small-spotted catshark embryonic stages used in this study does not require ethical permit and short-term housing of the eggs was approved by the local veterinary officer (Veterinäramt Kreis, Plön).

Consent for publication

Not applicable

Competing interests

The authors declare that they have no competing interests.

Funding

The authors acknowledge DESY (Hamburg, Germany), a member of the Helmholtz Association HGF, for the provision of experimental facilities where SRµCT was carried out at the PETRA III beamline P05. Beamtime was allocated for proposals I-20230087 and I-20240871.

Author contributions

E.E.V.	Conceptualization, formal analysis, funding acquisition, investigation, visualization, writing – original draft, writing – review and editing.
A.K.	Investigation, resources, writing – review and editing.
L.W.G.S.	Data curation, investigation, writing – review and editing.
A.P.M.R.	Investigation, writing – review and editing.
S.K.	Investigation, writing – review and editing.
J.U.H.	Methodology, software, writing – review and editing.
T.M.	Investigation, resources, writing – review and editing.
M.K.	Conceptualization, investigation, funding acquisition, project administration, supervision, writing – original draft, writing – review and editing.

Acknowledgements

E.E.V., L.W.G.S., A.P.M.R., S.K., and M.K. were supported by the Max Planck Society. The authors would like to thank animal caretakers Erika Teßmann and Ulrich Friese for their assistance and dedication in caring for the small-spotted catsharks in Ozeaneum (Stralsund), Martin Hansel and his team from Sea Life Berlin for donating small-spotted catshark eggs, and Denis Milla and Nela Kolčáková for their support with Avizo 3D Pro. This study was partly supported through the Maxwell computational resources operated at Deutsches Elektronen-Synchrotron DESY (Hamburg, Germany).

References

1. Coolen M, Sauka-Spengler T, Nicolle D, Le-Mentec C, Lallemand Y, Silva CD, et al.. Evolution of Axis Specification Mechanisms in Jawed Vertebrates: Insights from a Chondrichthyan. *PLoS One*. 2007; doi: 10.1371/journal.pone.0000374.

2. Square T, Jandzik D, Cattell M, Coe A, Doherty J, Medeiros DM. A gene expression map of the larval Xenopus laevis head reveals developmental changes underlying the evolution of new skeletal elements. *Dev Biol*. 2015; doi: 10.1016/j.ydbio.2014.10.016.

3. Piekarski N, Gross JB, Hanken J. Evolutionary innovation and conservation in the embryonic derivation of the vertebrate skull. *Nat Commun*. Nature Publishing Group; 2014; doi: 10.1038/ncomms6661.

4. Bhullar B-AS, Morris ZS, Sefton EM, Tok A, Tokita M, Namkoong B, et al.. A molecular mechanism for the origin of a key evolutionary innovation, the bird beak and palate, revealed by an integrative approach to major transitions in vertebrate history. *Evolution*. 2015; doi: 10.1111/evo.12684.

5. Kalinka AT, Tomancak P. The evolution of early animal embryos: conservation or divergence? *Trends in Ecology & Evolution*. 2012; doi: 10.1016/j.tree.2012.03.007.

6. Carroll SB. Evo-Devo and an Expanding Evolutionary Synthesis: A Genetic Theory of Morphological Evolution. *Cell*. 2008; doi: 10.1016/j.cell.2008.06.030.

7. Tanaka Y, Kudoh H, Abe G, Yonei-Tamura S, Tamura K. Evo-Devo of the Fin-to-Limb Transition. *Evolutionary Developmental Biology*. Springer, Cham;

8. Irion U, Nüsslein-Volhard C. Developmental genetics with model organisms. *Proceedings of the National Academy of Sciences*. Proceedings of the National Academy of Sciences; 2022; doi: 10.1073/pnas.2122148119.

9. Jenner RA, Wills MA. The choice of model organisms in evo–devo. *Nat Rev Genet*. Nature Publishing Group; 2007; doi: 10.1038/nrg2062.

10. Nojiri T, Fukui D, Werneburg I, Saitoh T, Endo H, Koyabu D. Embryonic staging of bats with special reference to Vespertilio sinensis and its cochlear development. *Developmental Dynamics*. 2021; doi: 10.1002/dvdy.325.

11. Anthwal N, Urban DJ, Sadier A, Takenaka R, Spiro S, Simmons N, et al.. Insights into the formation and diversification of a novel chiropteran wing membrane from embryonic development. *BMC Biology*. 2023; doi: 10.1186/s12915-023-01598-y.

12. Bai S, Li S, Li X, Zhu S, Shan Z, Zhang J, et al.. Comparison of embryonic development, from HH21 to HH40, between ostrich (Struthio camelus) and chicken (Gallus gallus). *Developmental Dynamics*. 2023; doi: 10.1002/dvdy.568.

13. Curantz C, Bailleul R, Castro-Scherianz M, Hidalgo M, Durande M, Graner F, et al.. Cell shape anisotropy contributes to self-organized feather pattern fidelity in birds. *PLOS Biology*. Public Library of Science; 2022; doi: 10.1371/journal.pbio.3001807.

14. Pranter R, Feiner N. Spatiotemporal distribution of neural crest cells in the common wall lizard Podarcis muralis. *Developmental Dynamics*. 2025; doi: 10.1002/dvdy.758.

15. Diaz Jr RE, Shylo NA, Roellig D, Bronner M, Trainor PA. Filling in the phylogenetic gaps: Induction, migration, and differentiation of neural crest cells in a squamate reptile, the veiled chameleon (Chamaeleo calyptratus). *Developmental Dynamics*. 2019; doi: 10.1002/dvdy.38.

16. Stundl J, Pospisilova A, Matějková T, Psenicka M, Bronner ME, Cerny R. Migratory patterns and evolutionary plasticity of cranial neural crest cells in ray-finned fishes. *Developmental Biology*. 2020; doi: 10.1016/j.ydbio.2020.08.007.

17. Horackova A, Pospisilova A, Stundl J, Minarik M, Jandzik D, Cerny R. Pre-mandibular pharyngeal pouches in early non-teleost fish embryos. *Proceedings of the Royal Society B: Biological Sciences*. Royal Society; 2023; doi: 10.1098/rspb.2023.1158.

18. Shimeld SM, Donoghue PCJ. Evolutionary crossroads in developmental biology: cyclostomes (lamprey and hagfish). *Development*. 2012; doi: 10.1242/dev.074716.

19. Lamanna F, Hervas-Sotomayor F, Oel AP, Jandzik D, Sobrido-Cameán D, Santos-Durán GN, et al.. A lamprey neural cell type atlas illuminates the origins of the vertebrate brain. *Nat Ecol Evol*. Nature Publishing Group; 2023; doi: 10.1038/s41559-023-02170-1.

20. Onai T, Adachi N, Urakubo H, Sugahara F, Aramaki T, Matsumoto M, et al.. Ultrastructure of the lamprey head mesoderm reveals evolution of the vertebrate head. *iScience*. 2023; doi: 10.1016/j.isci.2023.108338.

21. Marlétaz F, Timoshevskaya N, Timoshevskiy VA, Parey E, Simakov O, Gavriouchkina D, et al.. The hagfish genome and the evolution of vertebrates. *Nature*. Nature Publishing Group; 2024; doi: 10.1038/s41586-024-07070-3.

22. Onimaru K, Motone F, Kiyatake I, Nishida K, Kuraku S. A staging table for the embryonic development of the brownbanded bamboo shark (Chiloscyllium punctatum). *Developmental Dynamics*. 2018; doi: 10.1002/dvdy.24623.

23. Juarez M, Reyes M, Coleman T, Rotenstein L, Sao S, Martinez D, et al.. Characterization of the Trunk Neural Crest in the bamboo shark, Chiloscyllium punctatum. *J Comp Neurol*. 2013; doi: 10.1002/cne.23351.

24. Venkatesh B, Lee AP, Ravi V, Maurya AK, Lian MM, Swann JB, et al.. Elephant shark genome provides unique insights into gnathostome evolution. *Nature*. Nature Publishing Group; 2014; doi: 10.1038/nature12826.

25. Amaral CRL, Pereira ,Filipe, Silva ,Dayse A., Amorim ,António, and de Carvalho EF. The mitogenomic phylogeny of the Elasmobranchii (Chondrichthyes). *Mitochondrial DNA Part A*. Taylor & Francis; 2018; doi: 10.1080/24701394.2017.1376052.

26. Hirasawa T, Kuratani S. Evolution of Skeletal Tissues. *Evolutionary Developmental Biology*. Springer, Cham;

27. Hirasawa T, Kuratani S. Evolution of the vertebrate skeleton: morphology, embryology, and development. *Zoological Lett*. 2015; doi: 10.1186/s40851-014-0007-7.

28. Brazeau MD, Giles S, Dearden RP, Jerve A, Ariunchimeg Y, Zorig E, et al.. Endochondral bone in an Early Devonian 'placoderm' from Mongolia. *Nat Ecol Evol*. Nature Publishing Group; 2020; doi: 10.1038/s41559-020-01290-2.

29. Ryll B, Sanchez S, Haitina T, Tafforeau P, Ahlberg PE. The genome of Callorhinchus and the fossil record: a new perspective on SCPP gene evolution in gnathostomes. *Evol Dev*. 2014; doi: 10.1111/ede.12071.

30. Compagnucci C, Debiais-Thibaud M, Coolen M, Fish J, Griffin JN, Bertocchini F, et al.. Pattern and polarity in the development and evolution of the gnathostome jaw: Both conservation and heterotopy in the branchial arches of the shark, *Scyliorhinus canicula*. *Developmental Biology*. 2013; doi: 10.1016/j.ydbio.2013.02.022.

31. Ermakova GV, Meyntser IV, Zaraisky AG, Bayramov AV. Loss of noggin1, a classic embryonic inducer gene, in elasmobranchs. *Sci Rep*. Nature Publishing Group; 2024; doi: 10.1038/s41598-024-54435-9.

32. Gillis JA, Alsema EC, Criswell KE. Trunk neural crest origin of dermal denticles in a cartilaginous fish. *Proceedings of the National Academy of Sciences*. Proceedings of the National Academy of Sciences; 2017; doi: 10.1073/pnas.1713827114.

33. Cole NJ, Currie PD. Insights from sharks: Evolutionary and developmental models of fin development. *Developmental Dynamics*. 2007; doi: 10.1002/dvdy.21268.

34. : First observation of the mating, egg-laying and hatching behaviour of a captive female Kong skate, Okamejei kenojei (Müller & Henle, 1841) - Gao - 2022 - Journal of Fish Biology - Wiley Online Library. https://onlinelibrary.wiley.com/doi/full/10.1111/jfb.15165 Accessed 2025 Jun 6.

35. : The Elasmobranch Husbandry Manual: Captive Care of Sharks, Rays, and Their Relatives [1 ed.] 0867271523, 9780867271522. dokumen.pub. https://dokumen.pub/the-elasmobranch-husbandry-manual-captive-care-of-sharks-rays-and-their-relatives-1nbsped-0867271523-9780867271522.html Accessed 2025 Jun 6.

36. Gillis JA, Bennett S, Criswell KE, Rees J, Sleight VA, Hirschberger C, et al.. Big insight from the little skate: *Leucoraja erinacea* as a developmental model system. In: Goldstein B, Srivastava M, editors. *Current Topics in Developmental Biology*. Academic Press;

37. Maruska KP, Gelsleichter J. Chapter 11 - Hormones and Reproduction in Chondrichthyan Fishes. In: Norris DO, Lopez KH, editors. *Hormones and Reproduction of Vertebrates*. London: Academic Press;

38. Buddle AL, Dyke JUV, Thompson MB, Simpfendorfer CA, Whittington CM. Evolution of placentotrophy: using viviparous sharks as a model to understand vertebrate placental evolution. *Mar Freshwater Res*. CSIRO PUBLISHING; 2018; doi: 10.1071/MF18076.

39. Katona G, Szabó F, Végvári Z, Székely Jr T, Liker A, Freckleton RP, et al.. Evolution of reproductive modes in sharks and rays. *Journal of Evolutionary Biology*. 2023; doi: 10.1111/jeb.14231.

40. Pacoureau N, Rigby CL, Kyne PM, Sherley RB, Winker H, Carlson JK, et al.. Half a century of global decline in oceanic sharks and rays. *Nature*. Nature Publishing Group; 2021; doi: 10.1038/s41586-020-03173-9.

41. Ballard WW, Mellinger J, Lechenault H. A series of normal stages for development of *Scyliorhinus canicula* , the lesser spotted dogfish *(Chondrichthyes: Scyliorhinidae)*. *J Exp Zool*. 1993; doi: 10.1002/jez.1402670309.

42. Mayeur H, Leyhr J, Mulley J, Leurs N, Michel L, Sharma K, et al.. The Sensory Shark: High-quality Morphological, Genomic and Transcriptomic Data for the Small-spotted Catshark Scyliorhinus Canicula Reveal the Molecular Bases of Sensory Organ Evolution in Jawed Vertebrates. *Molecular Biology and Evolution*. 2024; doi: 10.1093/molbev/msae246.

43. Vidal-Vázquez N, Hernández-Núñez I, Carballo-Pacoret P, Salisbury S, Villamayor PR, Hervas-Sotomayor F, et al.. A single-nucleus RNA sequencing atlas of the postnatal retina of the shark Scyliorhinus canicula. *Sci Data*. Nature Publishing Group; 2025; doi: 10.1038/s41597-025-04547-2.

44. Mayeur H, Lanoizelet M, Quillien A, Menuet A, Michel L, Martin KJ, et al.. When Bigger Is Better: 3D RNA Profiling of the Developing Head in the Catshark Scyliorhinus canicula. *Front Cell Dev Biol*. Frontiers; 2021; doi: 10.3389/fcell.2021.744982.

45. Pearce J, Fraser MW, Sequeira AMM, Kaur P. State of Shark and Ray Genomics in an Era of Extinction. *Front Mar Sci*. Frontiers; 2021; doi: 10.3389/fmars.2021.744986.

46. Ziermann JM, Freitas R, Diogo R. Muscle development in the shark Scyliorhinus canicula: implications for the evolution of the gnathostome head and paired appendage musculature. *Frontiers in Zoology*. 2017; doi: 10.1186/s12983-017-0216-y.

47. Elagoz AM, Styfhals R, Maccuro S, Masin L, Moons L, Seuntjens E. Optimization of Whole Mount RNA Multiplexed in situ Hybridization Chain Reaction With Immunohistochemistry, Clearing and Imaging to Visualize Octopus Embryonic Neurogenesis. *Front Physiol*. Frontiers; 2022; doi: 10.3389/fphys.2022.882413.

48. Randlett O, Wee CL, Naumann EA, Nnaemeka O, Schoppik D, Fitzgerald JE, et al.. Whole-brain activity mapping onto a zebrafish brain atlas. *Nat Methods*. Nature Publishing Group; 2015; doi: 10.1038/nmeth.3581.

49. André M, Dinvaut S, Castellani V, Falk J. 3D exploration of gene expression in chicken embryos through combined RNA fluorescence in situ hybridization, immunofluorescence, and clearing. *BMC Biology*. 2024; doi: 10.1186/s12915-024-01922-0.

50. Matula J, Tesarova M, Zikmund T, Kaucka M, Adameyko I, Kaiser J. X-ray microtomography–based atlas of mouse cranial development. *GigaScience*. 2021; doi: 10.1093/gigascience/giab012.

51. Laznovsky J, Kavkova M, Helena Reis A, Robovska-Havelkova P, Maia LA, Krivanek J, et al.. Unveiling vertebrate development dynamics in frog Xenopus laevis using micro-CT imaging. *GigaScience*. 2024; doi: 10.1093/gigascience/giae037.

52. Metscher BD. MicroCT for developmental biology: A versatile tool for high-contrast 3D imaging at histological resolutions. *Developmental Dynamics*. 2009; doi: 10.1002/dvdy.21857.

53. Deeming DC, Kundrát M. Interpretation of fossil embryos requires reasonable assessment of developmental age. *Paleobiology*. 2023; doi: 10.1017/pab.2022.21.

54. Enault S, Adnet S, Debiais-Thibaud M. Skeletogenesis during the late embryonic development of the catshark Scyliorhinus canicula (Chondrichthyes; Neoselachii). *MorphoMuseum*. Association Palæovertebrata; 2016; doi: 10.18563/m3.1.4.e2.

55. Metscher BD. MicroCT for comparative morphology: simple staining methods allow high-contrast 3D imaging of diverse non-mineralized animal tissues. *BMC Physiology*. 2009; doi: 10.1186/1472-6793-9-11.

56. Callahan S, Crowe-Riddell JM, Nagesan RS, Gray JA, Davis Rabosky AR. A guide for optimal iodine staining and high-throughput diceCT scanning in snakes. *Ecology and Evolution*. 2021; doi: 10.1002/ece3.7467.

57. Babaei F, Hong TLC, Yeung K, Cheng SH, Lam YW. Contrast-Enhanced X-Ray Micro-Computed Tomography as a Versatile Method for Anatomical Studies of Adult Zebrafish. *Zebrafish*. Mary Ann Liebert, Inc., publishers; 2016; doi: 10.1089/zeb.2016.1245.

58. Criswell KE, Coates MI, Gillis JA. Embryonic development of the axial column in the little skate, *Leucoraja erinacea*. *Journal of Morphology*. 2017; doi: 10.1002/jmor.20637.

59. Gabner S, Böck P, Fink D, Glösmann M, Handschuh S. The visible skeleton 2.0: phenotyping of cartilage and bone in fixed vertebrate embryos and foetuses based on X-ray microCT. *Development*. 2020; doi: 10.1242/dev.187633.

60. Theodosiou NA, Oppong E. 3D morphological analysis of spiral intestine morphogenesis in the little skate, Leucoraja erinacea. *Developmental Dynamics*. 2019; doi: 10.1002/dvdy.34.

61. Rzhepakovsky I, Piskov S, Avanesyan S, Shakhbanov M, Sizonenko M, Timchenko L, et al.. High-Performance Microcomputing Tomography of Chick Embryo in the Early Stages of Embryogenesis. *Applied Sciences*. Multidisciplinary Digital Publishing Institute; 2023; doi: 10.3390/app131910642.

62. Matula J, Polakova V, Salplachta J, Tesarova M, Zikmund T, Kaucka M, et al.. Resolving complex cartilage structures in developmental biology via deep learning-based automatic segmentation of X-ray computed microtomography images. *Sci Rep*. Nature Publishing Group; 2022; doi: 10.1038/s41598-022-12329-8.

63. Kaucka M, Zikmund T, Tesarova M, Gyllborg D, Hellander A, Jaros J, et al.. Oriented clonal cell dynamics enables accurate growth and shaping of vertebrate cartilage. Bronner M, editor. *eLife*. eLife Sciences Publications, Ltd; 2017; doi: 10.7554/eLife.25902.

64. Kaucka M, Petersen J, Tesarova M, Szarowska B, Kastriti ME, Xie M, et al.. Signals from the brain and olfactory epithelium control shaping of the mammalian nasal capsule cartilage. White RM, editor. *eLife*. eLife Sciences Publications, Ltd; 2018; doi: 10.7554/eLife.34465.

65. Lesciotto KM, Perrine SMM, Kawasaki M, Stecko T, Ryan TM, Kawasaki K, et al.. Phosphotungstic acid enhanced microCT: optimized protocols for embryonic and early postnatal mice. *Dev Dyn*. 2020; doi: 10.1002/dvdy.136.

66. Tafforeau P, Boistel R, Boller E, Bravin A, Brunet M, Chaimanee Y, et al.. Applications of X-ray synchrotron microtomography for non-destructive 3D studies of paleontological specimens. *Appl Phys A*. 2006; doi: 10.1007/s00339-006-3507-2.

67. Betz O, Wegst U, Weide D, Heethoff M, Helfen L, Lee W-K, et al.. Imaging applications of synchrotron X-ray phase-contrast microtomography in biological morphology and biomaterials science. I. General aspects of the technique and its advantages in the analysis of millimetre-sized arthropod structure. *Journal of Microscopy*. 2007; doi: 10.1111/j.1365-2818.2007.01785.x.

68. Tesařová M, Mancini L, Simon A, Adameyko I, Kaucká M, Elewa A, et al.. A quantitative analysis of 3D-cell distribution in regenerative muscle-skeletal system with synchrotron X-ray computed microtomography. *Sci Rep*. Nature Publishing Group; 2018; doi: 10.1038/s41598-018-32459-2.

69. Leyhr J, Sanchez S, Dollman KN, Tafforeau P, Haitina T. Enhanced contrast synchrotron X-ray microtomography for describing skeleton-associated soft tissue defects in zebrafish mutants. *Front Endocrinol*. Frontiers; 2023; doi: 10.3389/fendo.2023.1108916.

70. Bejarano-Escobar R, Blasco M, Durán AC, Rodríguez C, Martín-Partido G, Francisco-Morcillo J. Retinal histogenesis and cell differentiation in an elasmobranch species, the small-spotted catshark Scyliorhinus canicula. *Journal of Anatomy*. 2012; doi: 10.1111/j.1469-7580.2012.01480.x.

71. Bejarano-Escobar R, Blasco M, Durán AC, Martín-Partido G, Francisco-Morcillo J. Chronotopographical distribution patterns of cell death and of lectin-positive macrophages/microglial cells during the visual system ontogeny of the small-spotted catshark Scyliorhinus canicula. *J Anat*. 2013; doi: 10.1111/joa.12071.

72. Wilde F, Ogurreck M, Greving I, Hammel JU, Beckmann F, Hipp A, et al.. Micro-CT at the imaging beamline P05 at PETRA III. *AIP Conference Proceedings*. 2016; doi: 10.1063/1.4952858.

73. Lytaev P, Hipp A, Lottermoser L, Herzen J, Greving I, Khokhriakov I, et al.. Characterization of the CCD and CMOS cameras for grating-based phase-contrast tomography. *Developments in X-Ray Tomography IX*. SPIE;

74. Moosmann J, Ershov A, Weinhardt V, Baumbach T, Prasad MS, LaBonne C, et al.. Time-lapse X-ray phase-contrast microtomography for in vivo imaging and analysis of morphogenesis. *Nat Protoc*. Nature Publishing Group; 2014; doi: 10.1038/nprot.2014.033.

75. van Aarle W, Palenstijn WJ, De Beenhouwer J, Altantzis T, Bals S, Batenburg KJ, et al.. The ASTRA Toolbox: A platform for advanced algorithm development in electron tomography. *Ultramicroscopy*. 2015; doi: 10.1016/j.ultramic.2015.05.002.

76. Aarle W van, Palenstijn WJ, Cant J, Janssens E, Bleichrodt F, Dabravolski A, et al.. Fast and flexible X-ray tomography using the ASTRA toolbox. *Opt Express, OE*. Optica Publishing Group; 2016; doi: 10.1364/OE.24.025129.

77. Palenstijn WJ, Batenburg KJ, Sijbers J. Performance improvements for iterative electron tomography reconstruction using graphics processing units (GPUs). *Journal of Structural Biology*. 2011; doi: 10.1016/j.jsb.2011.07.017.

78. Moosmann J. moosmann/matlab. https://github.com/moosmann/matlab. Accessed 16 April 2026.

79. Schindelin J, Arganda-Carreras I, Frise E, Kaynig V, Longair M, Pietzsch T, et al.. Fiji: an open-source platform for biological-image analysis. *Nat Methods*. Nature Publishing Group; 2012; doi: 10.1038/nmeth.2019.

80. Schmied C, Jambor HK. Effective image visualization for publications – a workflow using open access tools and concepts. F1000Research;

81. Tesařová M, Zikmund T, Kaucká M, Adameyko I, Jaroš J, Paloušek D, et al.. Use of micro computed-tomography and 3D printing for reverse engineering of mouse embryo nasal capsule. *J Inst*. 2016; doi: 10.1088/1748-0221/11/03/C03006.

82. Pétré M, Balcaen T, Schneidewind P, Mazy L, Pyka G, Fehervary H, et al.. Screening staining agents for contrast-enhanced microCT of vascular tissues: Assessing the effect on microstructural and mechanical properties. *Tomography of Materials and Structures*. 2024; doi: 10.1016/j.tmater.2024.100038.

83. Balint R, Lowe T, Shearer T. Optimal Contrast Agent Staining of Ligaments and Tendons for X-Ray Computed Tomography. *PLOS ONE*. Public Library of Science; 2016; doi: 10.1371/journal.pone.0153552.

84. Choi HMT, Schwarzkopf M, Fornace ME, Acharya A, Artavanis G, Stegmaier J, et al.. Third-generation in situ hybridization chain reaction: multiplexed, quantitative, sensitive, versatile, robust. *Development*. 2018; doi: 10.1242/dev.165753.

85. Becker K, Jährling N, Saghafi S, Weiler R, Dodt H-U. Chemical clearing and dehydration of GFP expressing mouse brains. *PLoS One*. 2012; doi: 10.1371/journal.pone.0033916.

86. Escamilla-Vega E, Seton LWG, Kyomen S, Murillo-Rincón AP, Petersen J, Tautz D, et al.. Evolution of the essential gene MN1 during the macroevolutionary transition toward patterning the vertebrate hindbrain. *Proceedings of the National Academy of Sciences*. Proceedings of the National Academy of Sciences; 2025; doi: 10.1073/pnas.2416061122.

87. Byrum SR, Frazier BS, Grubbs RD, Naylor GJP, Fraser GJ. Embryonic development in the bonnethead (Sphyrna tiburo), a viviparous hammerhead shark. *Developmental Dynamics*. 2024; doi: 10.1002/dvdy.658.

88. Fedorov A, Beichel R, Kalpathy-Cramer J, Finet J, Fillion-Robin J-C, Pujol S, et al.. 3D Slicer as an image computing platform for the Quantitative Imaging Network. *Magn Reson Imaging*. 2012; doi: 10.1016/j.mri.2012.05.001.

89. Cignoni P, Callieri M, Corsini M, Dellepiane M, Ganovelli F, Ranzuglia G. MeshLab: an Open-Source Mesh Processing Tool. The Eurographics Association;

90. Escamilla-Vega E, Koch A-K, Seton LWG, Murillo-Rincón AP, Kyomen S, Hammel JU, et al.. Supporting data for "Synchrotron radiation micro-computed tomography of the small-spotted catshark embryonic development (Chondrichthyes: Scyliorhinus canicula)" GigaScience Database. https://doi.org/10.5524/102817.

91. Li X, Hua C, Yue Q, Li Z, Tong J, Luo Z, et al. GigaDB: A redesigned repository for data publishing and management. GigaScience. 2026; giag047. doi:10.1093/gigascience/giag047

92. Mellinger J. Egg-case diversity among dogfish, Scyliorhinus canicula (L.): a study of egg laying rate and nidamental gland secretory activity. *Journal of Fish Biology*. 1983; doi: 10.1111/j.1095-8649.1983.tb04728.x.

93. Koch A-K, Grunow B, Moritz T. Recommendations for scientific fish husbandry: Scyliorhinus canicula (Carcharhiniformes, Scyliorhinidae) – a model species for sharks. *Bulletin of Fish Biology*. 2025; doi: doi: 10.53188/BFB0012.

Figure legends

Figure 1. Experimental setup for SRµCT of small-spotted catshark embryos. (A) Fertilized small-spotted catshark eggs. Small-spotted catshark eggshells display a broad range of coloration from light beige to dark brown [92,93]. The underlying causes of this phenotypic variation remain unknown, although genetic factors have been suggested as contributing influences [92]. Importantly, eggshell

pigmentation does not affect embryo development [93]. White arrowhead indicates an embryo at approximately St.28 within the eggcase. Scale bar: 1cm. **(B)** St.31 small-spotted catshark embryo, attached to its yolk sac and removed from the eggcase. Scale bar: 1cm. **(C)** PTA-contrasted small-spotted catshark embryos at two developmental stages: St.28 (left), and St.15 (right, indicated by black arrowhead). The embryos are mounted in plastic pipette tips and glued to standardized holders. Scale bar: 1cm. **(D)** Experimental setup at the beamline P05 at PETRA III. Red arrowhead indicates the sample positioned in the center of the stage prior to imaging. **(E)** St.15 small-spotted catshark embryo from Fig. 1C immediately after scanning. Scale bar: 1.5mm. **(F)** Raw tomographic slice of PTA-contrasted St.30 small-spotted catshark embryo (left) and the same tomographic slice after pre-processing in ImageJ/Fiji (right). The dotted white circles indicate the pipette tip containing the embryo. Thresholds for the adjustment of brightness and contrast are shown in blue (low-intensity value) and red (high-intensity value), with the original values shown for the unprocessed image. Scale bar: 500µm.

Figure 2. 3D reconstruction of a fully segmented St.19 small-spotted catshark embryo. (A) 3D reconstruction of all segmented structures together. Note that some structures may not visible in this model because they are located beneath other anatomical structures. Scale bar: 500µm. **(B)** 3D reconstructions of segmented structures presented separately for better visibility of individual organs and tissues. Scale bar: 500µm.

Figure 3. Combination of SRµCT with 3D confocal laser microscopy gene expression data. (A) 3D reconstruction of the developing small-spotted catshark nervous system at St.23. Blue 3D models represent the segmented central nervous system (CNS). Red 3D models represent the segmented peripheral nervous system (PNS). Scale bars: 1.5mm. A’ and A’’ indicate tomographic slices of the pre-processed SRµCT data used to reconstruct the nervous system. Scale bar: 250µm. **(B)** TUJ1

immunofluorescence labelling the PNS and immature neurons of the CNS at St.23. Scale bar: 1.5mm. **(C)** HCR *in situ* hybridization labelling different subpopulations within the developing nervous system at St.23. Scale bar: 1mm.

Figure 4. Examples of 3D reconstructions and tomographic slices of small-spotted catshark embryos at different developmental stages. Yellow labelling indicates distinct developing structures. Red dashed lines indicate the anatomical position corresponding to the tomographic slices. **(A)** St.12 small-spotted catshark embryo. Dorsal view 3D reconstruction (left) and tomographic slice (right). Scale bars: 500µm. **(B)** St.15 small-spotted catshark embryo. Dorsal view 3D reconstruction (left) and tomographic slices (right). Tomographic slice 1 through the prospective head region shows the elevation of the neural plate at this stage. Tomographic slice 2 through the trunk region shows the formation of early developmental structures like the somites and primitive gut. Scale bars: 500µm. **(C)** St.25 small-spotted catshark embryo. Whole embryo lateral view 3D reconstruction (left) and close-up of the head region (middle). Scale bars: 1mm. Tomographic slice through the trunk region, where the pectoral fin buds are located, and close-up (right). Scale bars: 500µm. **(D)** Ventral and lateral view 3D reconstruction of the head of a St.30 small-spotted catshark embryo (left) and corresponding tomographic slices (right). Scale bars: 500µm.

Figure 5. Heart morphogenesis in the small-spotted catshark. 3D reconstructions of whole embryos in lateral view at consecutive developmental stages (top) and corresponding frontal view of the segmented heart (bottom). Imaging of consecutive embryonic stages allowed the reconstruction of heart morphogenesis, from a nearly straight tube at St19, to the bending into an S-shape at St.21-22, and the acquisition of a compact structure by St.24-25. Scale bars: 500µm.

Main Figure 1

Click here to access/download;Figure;Fig1.tiff

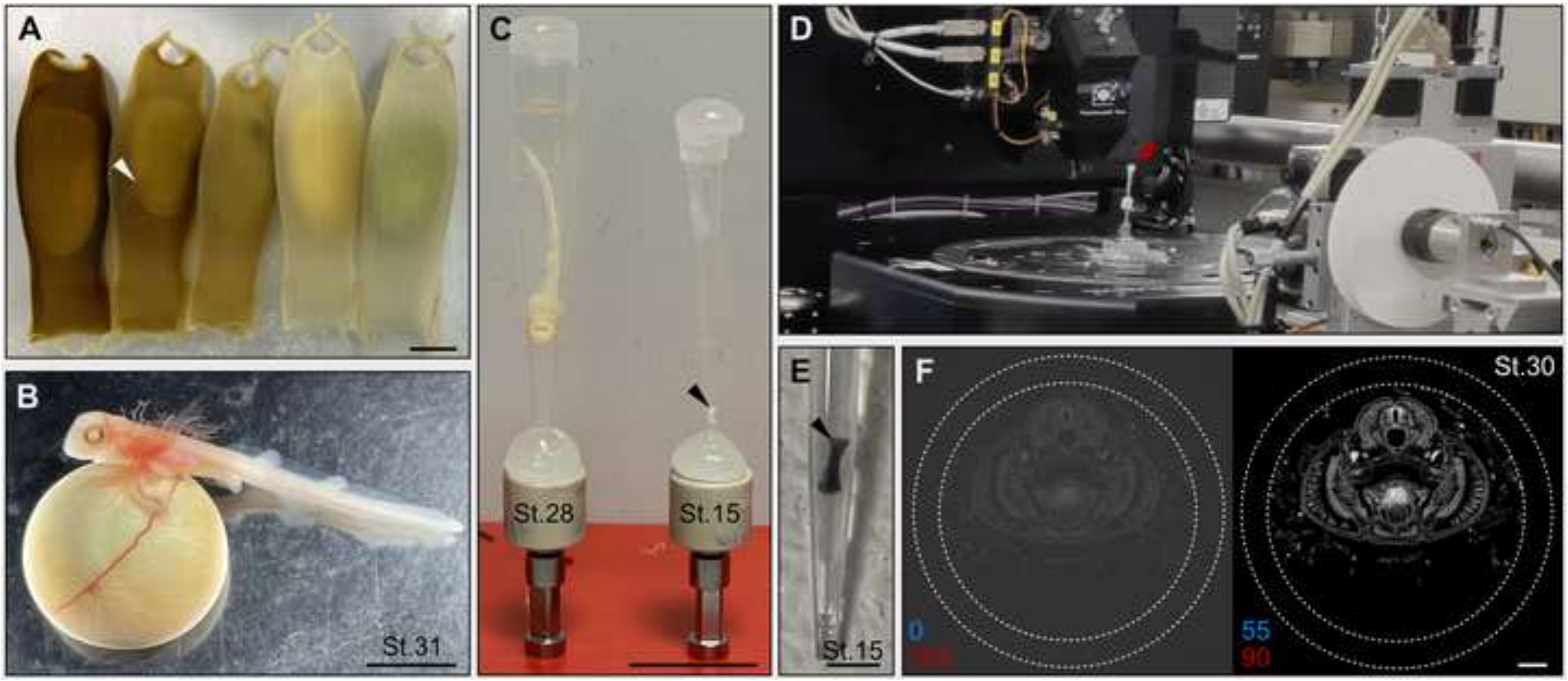

Main Figure 2

Click here to access/download;Figure;Fig2.tiff

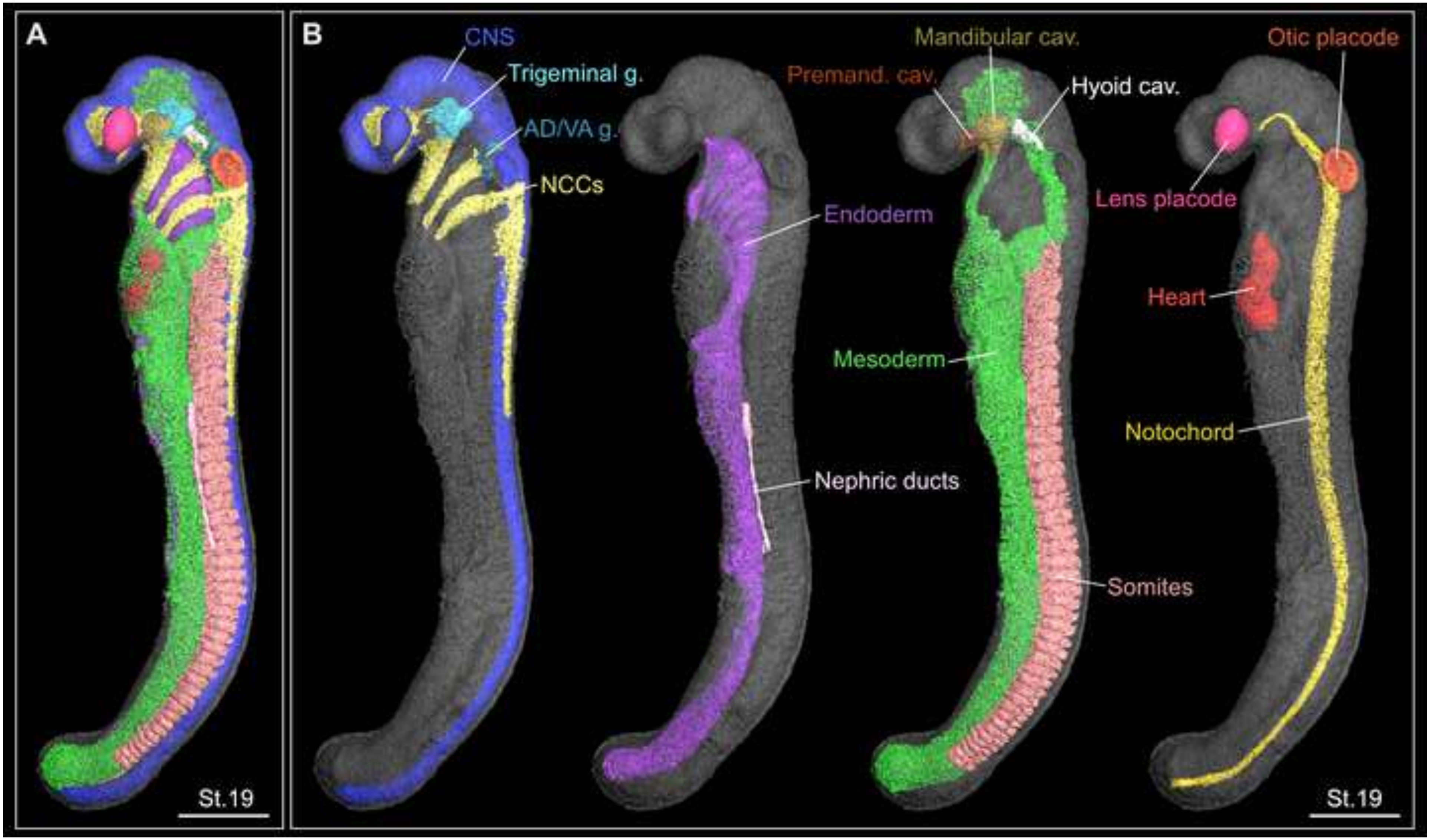

Main Figure 3

Click here to access/download;Figure;Fig3.tiff

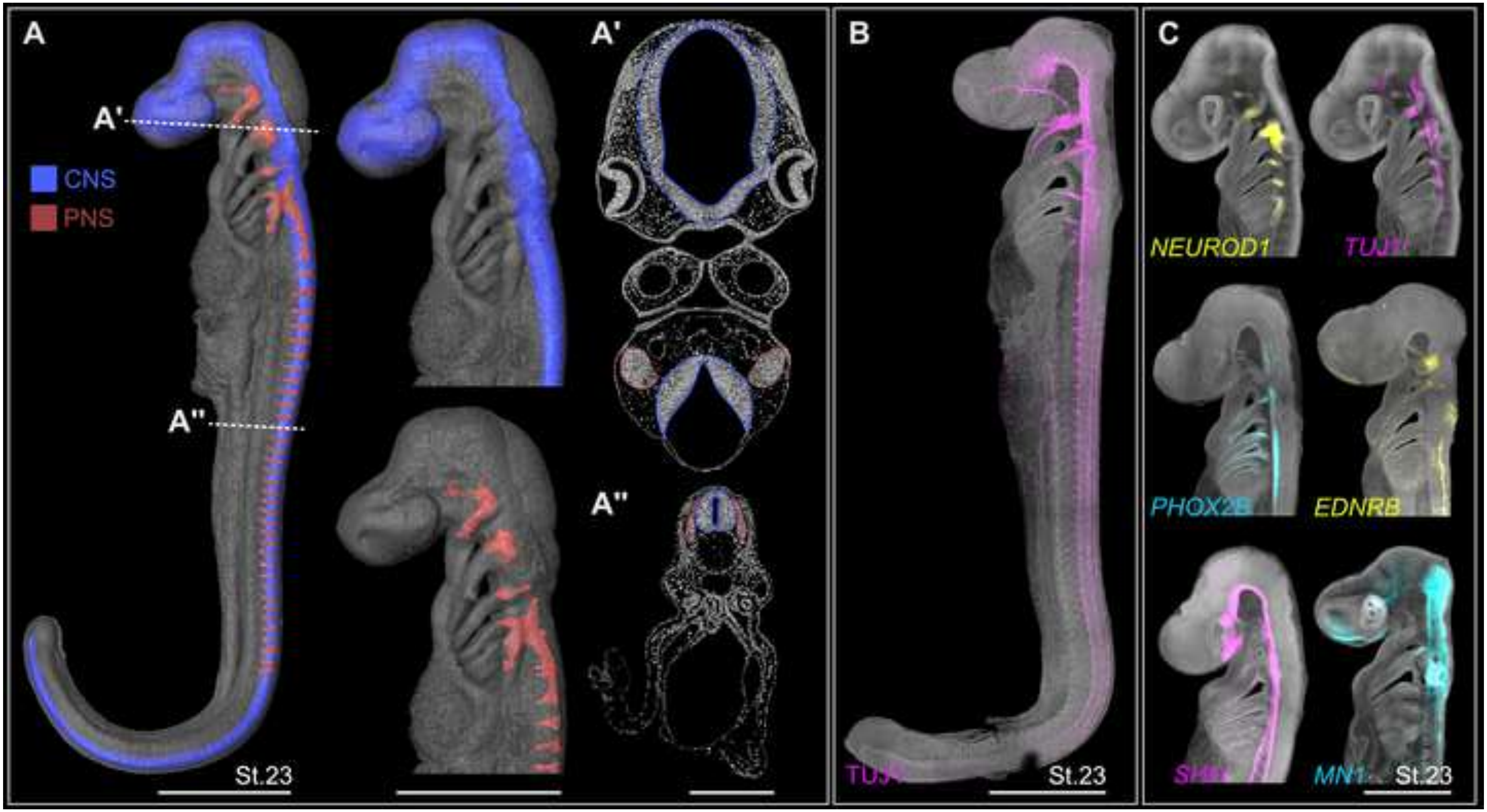

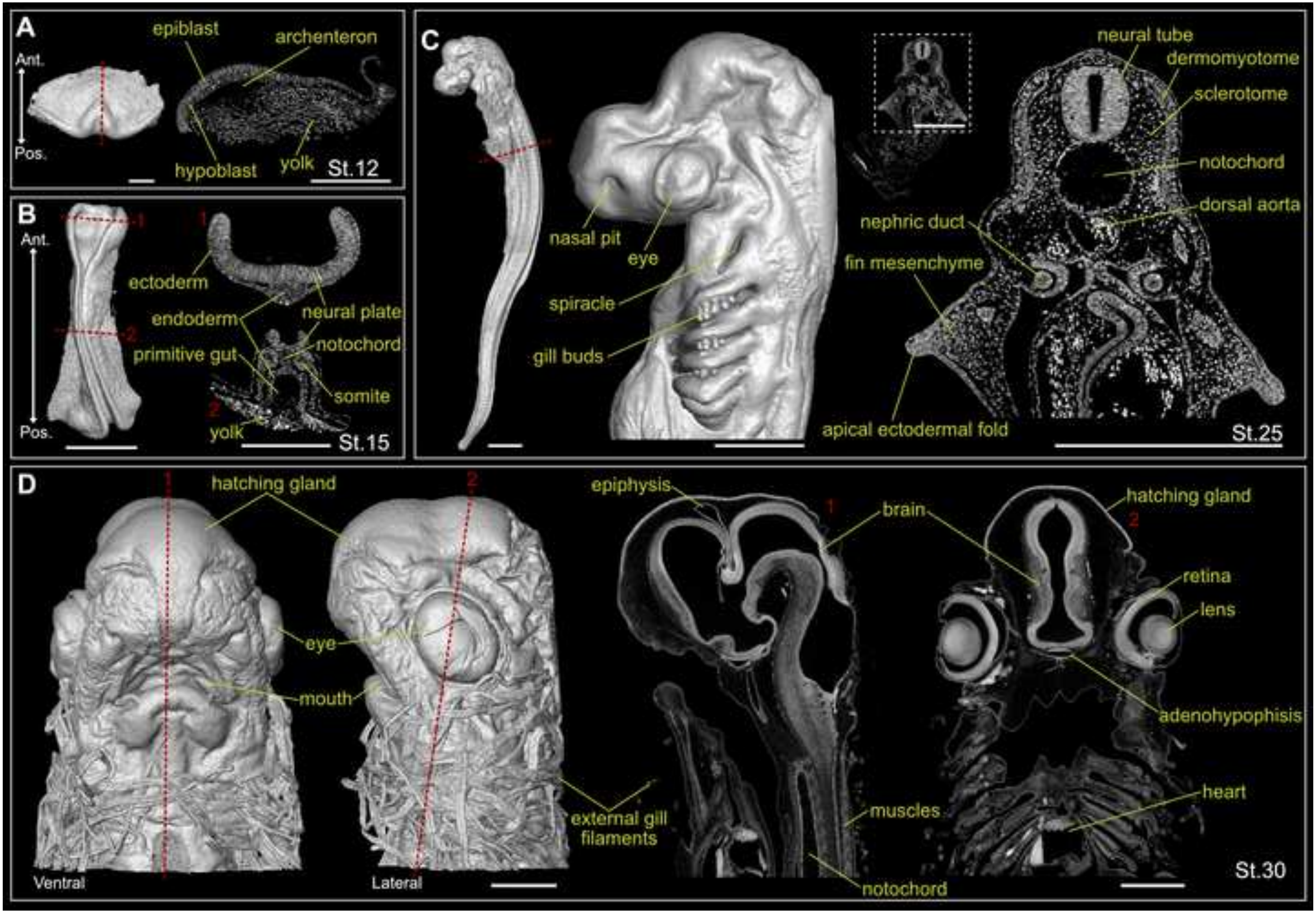
A
Ant.
Pos.
epiblast
archenteron
hypoblast
yolk
St.12
B
Ant.
Pos.
ectoderm
endoderm
primitive gut
neural plate
notochord
somite
yolk
St.15
C
nasal pit
eye
spiracle
gill buds
neural tube
dermomyotome
sclerotome
notochord
dorsal aorta
nephric duct
fin mesenchyme
apical ectodermal fold
St.25
D
hatching gland
eye
mouth
external gill filaments
Ventral
Lateral
epiphysis
brain
muscles
notochord
hatching gland
retina
lens
adenohypophysis
heart
St.30

Click here to access/download;Figure;Fig5.tiff

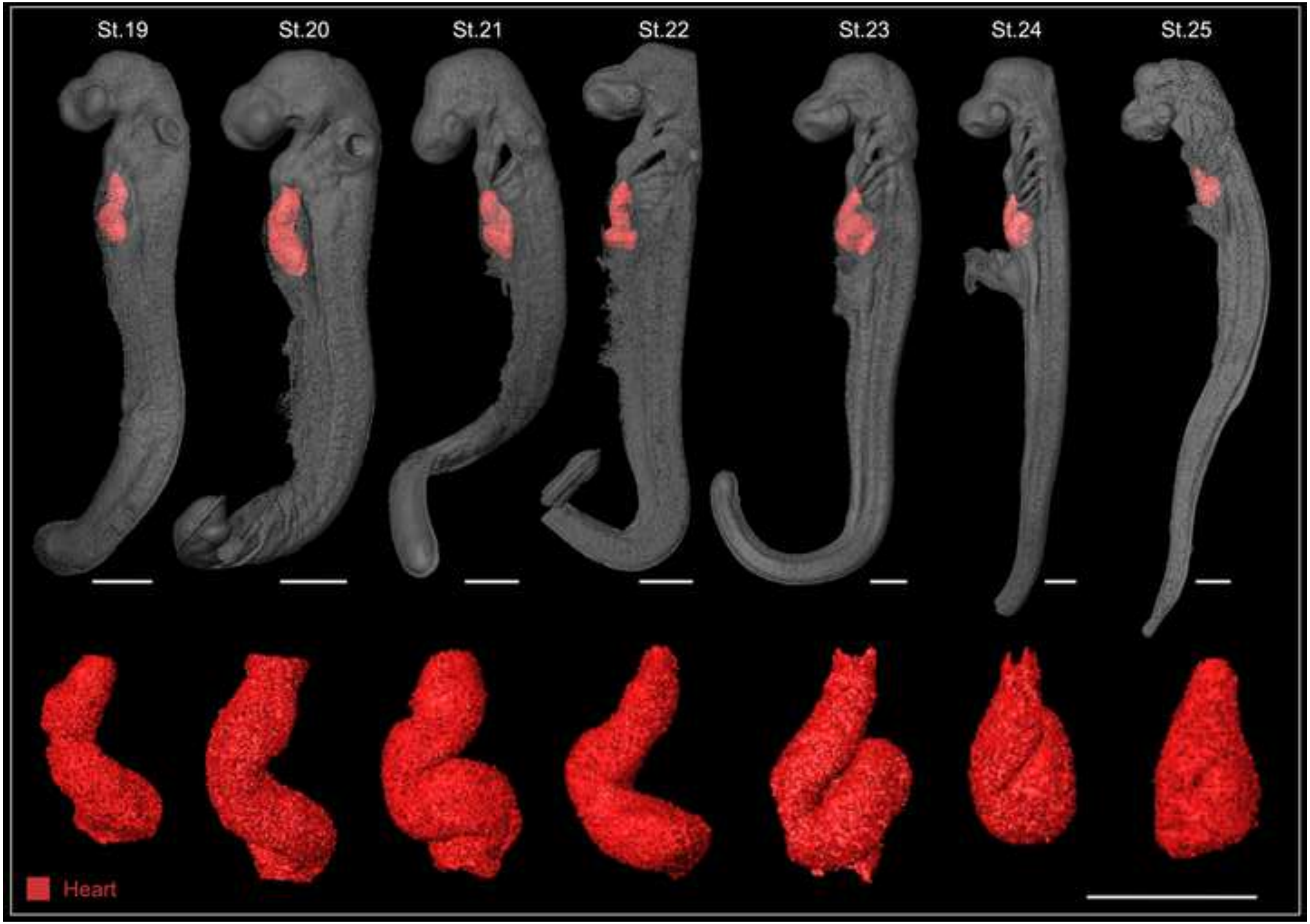

Click here to access/download
Supplementary Material
SupplementaryFiles_GigaScience_14_02_2026.docx

Supplementary Figure 1

Click here to access/download
Supplementary Material
Supplementary_Figure_1.tiff

Supplementary Figure 2

Click here to access/download
Supplementary Material
Supplementary_Figure_2.tiff

Supplementary Figure 3

Click here to access/download
Supplementary Material
Supplementary_Figure_3.tiff

Cover Letter

Click here to access/download;Personal Cover;CoverLetter_GigaScience_14_02_2026.pdf

MAX PLANCK INSTITUTE
FOR EVOLUTIONARY BIOLOGY

To: Dr. Hongfang Zhang
GigaScience

Dr. Markéta Kaucká

Max Planck Research Leader Group
MPI for Evolutionary Biology
August-Thienemann-Str. 2
D-24306 Plön

Tel.: 00 49-(0) 15203 100 942
kaucka@evolbio.mpg.de

February 14, 2026

Dear Dr. Zhang,

Thank you very much for the opportunity to resubmit our revised manuscript "Synchrotron radiation micro-computed tomography of the small-spotted catshark embryonic development (Chondrichthyes: *Scyliorhinus canicula*)" (Manuscript ID: GIGA-D-25-00317).

We thank you and the reviewers for the careful evaluation of our work and for the constructive comments and suggestions. We were delighted that the reviewers found our work interesting, important, and of high quality. We have revised the manuscript accordingly and believe that the changes have significantly improved its clarity and completeness. A detailed, point-by-point response to all reviewer comments is provided in the Response to Reviewers document, and all modifications in the manuscript have been clearly indicated.

We hope that the revised manuscript is now suitable for publication in *GigaScience*, and we appreciate your time and consideration. We believe that the presented unique dataset of high-resolution 3D images of developing small-spotted catshark will form a foundation for future evolutionary, developmental, and comparative studies and will be of broad interest to the readership of *GigaScience* and across disciplines.

If you require any additional information, please do not hesitate to contact me.
Many thanks for your time and for considering our work for publication in *GigaScience*.

Sincerely,
Markéta

Kaucká Markéta
