# Supplementary material for: Synchrotron radiation micro-computed tomography of the small-spotted catshark embryonic development (Chondrichthyes: Scyliorhinus canicula)
Source: Gigascience. 2026 May 7;15:giag054. doi: 10.1093/gigascience/giag054 (PMC13240850; doi:10.1093/gigascience/giag054)
Supplement: giag054_Authors_Response_To_Reviewer_Comments_original_submission [file giag054_authors_response_to_reviewer_comments_original_submission.pdf]

Author's Response To Reviewer Comments

Response to the Reviewers
We thank the Reviewers for their careful and thoughtful evaluation of our manuscript and for their constructive feedback. We have revised the manuscript accordingly and responded to each of their comments below.

Reviewer #1

The authors describe a 3D developmental image series of Scyliorhinus canicula, an important research animal. This is certainly a valuable data set, well documented and accessible. The images are of sufficient completeness and detail to be useful to other researchers. X-ray microtomographic images are an excellent complement to other modes of visualization. The report is well written and sufficient for the purpose.

We would like to cordially thank Reviewer #1 for their positive assessment of our manuscript and for their valuable and constructive suggestions.

A few specific items that merit attention:

p. 7: Sample shrinkage is always an issue with whole-mount imaging of soft specimens and PTA is well known to shrink tissues significantly. Were there any measurements of shrinkage or distortion, or comparisons with unstained samples? This would be worth mentioning.

Tissue shrinkage is indeed a known, unavoidable technical limitation of PTA-contrasting [1–3], and a slight tissue distortion is expected across samples and tissues. However, tissue shrinkage does not result in differences in the relative positions of organs or in the order of their appearance during embryogenesis. We absolutely agree with the reviewer that it would have been very valuable to scan all the embryos prior to PTA-contrasting; however, due to the strict time limitations imposed by the granted funding and assigned beamtime, we could not carry out such scans and measurements to focus specifically on this issue. We now explicitly mention this phenomenon in the text and point the readers to the known effect of contrasting agents and tissue shrinkage (see lines 227-234 or quoted text below). Additionally, we included a new Supplementary Figure showing examples of tissue distortion (Supplementary Fig. 2).

“A slight tissue shrinkage was observed due to the overall tissue dehydration and PTA-contrasting enhancement (1.5% PTA in 90% methanol), which represents a known, unavoidable technical limitation of enhanced soft tissue contrast [65,81,82]. This effect was particularly evident in ectodermal structures starting at St.25 (Supplementary Fig. 2). However, despite the slight tissue shrinkage, the overall morphology and relative anatomical position of the different embryonic structures remain intact. Moreover, the relative order in which individual structures emerge along the developmental timeline are not changed, important for studies on developmental heterochrony.”

Line 163: Degrees are more conventional in practical measurements of angles

Thank you for this suggestion, we have modified the text and added the information in degrees.

p. 8 lines 187f.: Could say a bit more about how the brightness and contrast ranges were chosen: e.g. to suppress background, how much saturation, and refer to Fig. 1 F.

In response to your comment, we have incorporated an additional Supplementary Figure (Supplementary Fig. 1) where we show and explain how the brightness and contrast were adjusted using the Brightness/Contrast function in ImageJ/Fiji 2.9.0 following the guidelines from [4]. This function allows for modifying (clipping) the intensity range (maximum and minimum values) but does not provide information about saturation. We have also included the intensity thresholds selected for the original and pre-processed tomographic slices in Fig. 1F.

p. 11 lines 257-9 Two specimens are not enough to claim more than a baseline o starting point for studying variation.

We agree with the reviewer and have removed the statement from the text. Instead, we now emphasize the potential use of the dataset to investigate within-stage differences in future studies (see lines 309-316 or quoted text below). This is accompanied by a new Supplementary Figure (Supplementary Fig. 3) in which we present an example of within-stage differences, namely neural tube closure at early- and mid-St.17, to bring this topic to the attention of the readers.

"Small-spotted catshark embryos at the same developmental stage often exhibit slight morphological differences depending on their precise developmental timing. For instance, early St.26 embryos (approximately 37 days after deposition) have approximately 85 pairs of somites, while late St.26 embryos (approximately 42 days after deposition) have around 90 pairs of somites [41]. This within-stage variation highlights that embryonic development is a continuous process despite being conventionally subdivided into discrete stages. The presented dataset can serve as a starting point for future studies of within-stage developmental differences since it provides scans of at least two independent embryos from St.17-29, allowing a more refined characterization of small-spotted catshark development (Supplementary Fig. 3)."

p. 12 line 277 Do the archived datasets include the label files (the segmentations)? Good to mention this as it increases the value of the image data.

The datasets include the raw and pre-processed tomographic images, but not the segmentation files. Given the dataset's scientific relevance, we wanted to make it publicly available as soon as possible and provide a valuable resource to the evo-devo community. We agree with the Reviewer that providing the segmentations would increase the value of the dataset, and we are currently working towards an extensive segmentation resource. However, due to the large dataset spanning several developmental stages and the numerous embryonic structures to be segmented, these data are far from complete. All such segmentations, accompanied by extensive molecular mapping, will be summarized in a separate manuscript and made publicly available in the future.

Are the original specimens still intact and vouchered somewhere?

The PTA-contrasted embryos were not retained for long-term storage after SRμCT imaging. During synchrotron scanning, the specimens received a high X-ray dose, which is known to induce radiolysis of water and organic material and to chemically modify PTA (visible as the formation of "tungsten blue") (Fig. 1E). These radiation-induced changes can alter both the tissue and the staining chemistry and may continue to develop during further storage, making the specimens unsuitable for subsequent quantitative imaging, histology, or other analyses.
In addition, PTA staining is not readily reversible, and prolonged storage of contrasted/fixed/scanned embryonic material often leads to further diffusion of stain, precipitation, or tissue brittleness, which compromises morphology. After weighing the pros and cons of further storage, we opted to discard these samples safely.

Fig. 3F: Thank you for showing pre- and post-processed images.

We thank the Reviewer for the positive feedback.

Reviewer #2

This manuscript presents a high-resolution dataset of embryonic development in the small-spotted catshark (Scyliorhinus canicula), an emerging important model for studying vertebrate development and evolution. Using synchrotron radiation micro-computed tomography (SRμCT), 36 whole-embryo scans were obtained across key developmental stages (gastrulation to late organogenesis, stages 12-31). Enhanced tissue contrast with phosphotungstic acid enabled high-level resolution, allowing semi-automated reconstruction and pre-processing for 3D segmentation of anatomical structures during the development.

The dataset has a potential to provide a comprehensive resource for morphometric analyses and comparative evo-devo research, facilitating the identification of conserved and derived developmental processes in vertebrates.

This dataset has a great potential to enhance the evo-devo research on catshark, however, there are several major points that need to be addressed by the authors.

We would like to cordially thank Reviewer #2 for their positive assessment of the manuscript and for their valuable comments and suggestions, which helped us improve its clarity and address its limitations.

Major points:

1. It is important to make it clear that this is not the first attempt to image catshark embryos with a synchrotron radiation micro-computed tomography by using enhanced tissue contrast. For example, the recent paper by Mayeur et al (2024) described the 3D segmentation of the catshark late embryo sensory structures obtained by this technique using another contrast agent. Even though the authors cite this paper, it is not made clear in the text. However, I agree that this a first catshark embryo dataset spanning a large range of developmental stages that provides a great comparative potential.

We thank the Reviewer for pointing this out and apologize for not discussing the publication by Mayeur et al. (2024) sufficiently. While the publication was indeed cited in the original manuscript, we agree that its relevance was not adequately discussed. We have introduced the following text to acknowledge its contributions.

“Synchrotron radiation micro-computed tomography (SRµCT) combined with iodine-based contrast enhancement was previously employed to reconstruct and characterize the sensory organs in a pre-hatching small-spotted catshark embryo [42], highlighting the potential of this imaging technology to study chondrichthyan development in great detail.”

2. Catshark is an important vertebrate model, however it is very derived and during the evolution of vertebrates cartilaginous fishes lost perichondral, all major dermal and possibly even the endochondral bones (Ryll et al., 2014; Brazeau et al., 2020). This is important to add to the description of the species, to be able to describe for example the skeletal structures in the catshark embryo dataset.

We agree with the reviewer and, in response to this comment, we have added information on the evolution of skeletal tissues to the description of Chondrichthyes.

“Chondrichthyans are primarily known for having a cartilaginous endoskeleton throughout their lives, and the loss of perichondral, endochondral, and most dermal bones [26–29]. These traits render them important models for the research of skeletal tissue evolution.”

3. The information on image resolution, tissue enhancement properties of PTA and identified anatomical structures requires editing.

3.1 Authors state in several places in the text that they have acquired a dataset with a cellular resolution, however this would require a resolution below 1 micron. It is also visible from the virtual histology sections on Figure 3 and 4 for example, that the resolution in not at the cellular level. This statement should be changed. In addition, a virtual histology section on Figure 4D has a lower resolution comparing to other virtual histology sections presented and should be replaced, if possible.

We agree with the reviewer and have carefully revised the manuscript and removed all references to “cellular resolution”. We also apologize for the lower resolution of the tomographic slice shown in Fig. 4D and have replaced it with a higher-quality one.

We would like to note that scans from St.26-31 were acquired at a lower resolution (2.57µm) than those from St.12-25 (1.28µm), which likely explains why the tomographic slice in Fig. 4D appears slightly different from the others. However, we have ensured that the revised figure/panels are representative and

of an appropriate quality.

3.2 It should be made clear in the text that PTA might not provide a good contrast for all types of tissues. In this dataset for example it is not well suitable for contrasting skeletal tissues, like muscle, and cartilage, which is visible on virtual histology sections on Figure 4.
Showing a few corresponding slides from a traditional histology for comparison would be valuable if authors would like to discuss PTA contrast as universal for all tissue types.

Thank you for this comment, we have clarified in the manuscript that different contrasting agents exhibit distinct tissue-specific absorption rates and contrasting properties, and we explicitly note that PTA does not stain cartilage – please see this adapted text:

“This limitation can be overcome by using contrasting agents, which are differentially absorbed by soft tissues, allowing their visualization in the final images [55]. Various contrast-enhancing agents have been tested, including iodine [51,56–58], ruthenium red [59], and phosphotungstic acid (PTA) [50,60,61], each with distinct tissue-specific absorption rates and, thus, contrasting abilities (e.g., PTA does not stain cartilage) [62–65].”

However, we would like to note that PTA has previously been successfully applied to the study of skeletal tissues, specifically, because cartilage remains uncontrasted while the surrounding softer tissues exhibit strongly enhanced contrast, allowing the identification and segmentation of skeletal structures (e.g., cartilage) based on the negative contrast [1,5–7]. We would like to further note that muscles do absorb PTA, as shown in the tomographic slice in Fig. 4 and in other publications [8].

Given the existing literature on this topic and because the aim of this manuscript is to present the small-spotted catshark developmental SRμCT dataset (rather than to compare or evaluate contrasting agents), we refrained from including traditional histological sections. We provide relevant references describing the benefits and disadvantages of PTA and contrasting different tissues in the manuscript.

3.3 There are no labeled teeth, skin denticles, tendons, liver, reproductive organs in the Figures, despite that the authors have included these tissues in a long list of tissues and structures that can be analyzed in this dataset (Lines 232-239). The suggestion is to edit this list of tissues and structures to include only the identified tissues in the dataset and all tissues mentioned should be also labeled in the Figures.

We thank the Reviewer for pointing out that the list of tissues and structures (lines 232-239, now 291-296) could be interpreted as referring only to structures explicitly labelled in the figures. Our intention was different: this list was meant to indicate the broader range of anatomical features that can be identified in the full dataset and therefore its potential for reuse, whereas the figures were designed only to show representative examples of some of the identifiable structures rather than to provide a comprehensive anatomical atlas.
Several tissues listed (e.g., teeth, skin denticles, liver, and reproductive organs) are present in the dataset at their respective developmental stages and anatomical regions, but were not specifically labelled in the figures to maintain figure clarity and readability. Labelling all identifiable structures across multiple stages would substantially reduce legibility without improving the interpretation of the example images.

To avoid confusion, we have revised the manuscript to clarify that (i) the list of tissues and structures refers to features that can be identified in the dataset as a whole and highlights potential avenues for reuse, and (ii) the figures illustrate selected representative structures rather than an exhaustive inventory. We believe this revision addresses the Reviewer’s concern while preserving both figure clarity and the intended description of the dataset’s scope.

4. Include mid-lateral virtual histology sections on Figure 3 and 4?

We thank the Reviewer for this suggestion. A mid-lateral virtual histology section has now been included in Fig. 4D to provide an additional anatomical perspective. We have not added a comparable section to Fig. 3 because the figure is already densely populated, and inclusion of an additional panel would reduce clarity and readability. In Fig. 3, the coronal sections were intentionally chosen, as they provide a clearer

visualization of both central and peripheral components of the nervous system, which are the primary features highlighted in this figure. We hope that the additional section in Fig. 4, together with the existing views in Fig. 3, provides sufficient anatomical context while maintaining figure clarity.

5. Because the authors emphasize that ''The segmented 3D models can be directly compared between consecutive developmental stages to understand the complex morphogenetic processes during embryogenesis together with organ growth and shaping.'', by providing a 3D reconstruction of several consecutive stages showing organ growth and shaping changes (for example inner organs like heart and additional stages with segmented CNS) they would demonstrate this. The stages should be clearly indicated in the Figures and not only in the Figure legend, which will also make a comparative aspect more pronounced.

We fully agree with the Reviewer and have now included a new main figure (Fig. 5) illustrating the process of heart morphogenesis. In this figure, we segmented the developing heart from St.19 to St.25 and show how it changes from an initial near-straight tube, through an S-shape, to a compact structure more similar to its final morphology. In addition, following the Reviewer's suggestion, we have clearly indicated the corresponding developmental stages directly within the figure (and not only in the figure legend), as well as in all other figures.

6. Additional information is required for the growth series provided in Supplementary Table 2, this should include the length of the specimen and other important parameters like somite number.

We have incorporated embryo length and somite number into Supplementary Table 2. Although counting somites in early-developing catshark embryos is relatively easy, at later developmental stages, the anterior somites begin to differentiate, and only the posterior ones are recognizable. For this reason, and to avoid introducing potential errors, somite counts are provided up to St.25.

7. Author state that all data are publicly available for further exploration. However, there was no link available for reviewer for deposited raw or pre-processed small-size datasets. Therefore, the availability of all datasets described in Supplementary Table 2 could not be confirmed.
It is important that the authors make available the image stacks before and after processing i.e. of large and small size, since file size reduction might have resulted in the image quality reduction.

We sincerely apologize and regret that the Reviewer was unable to access the dataset during the review process. Due to the large size of the raw dataset, we were initially instructed by the editorial office (specifically the data curator) to provide only the pre-processed files. These files should have been made available to the Reviewers.

We fully agree with the Reviewer that both raw image stacks and pre-processed datasets should be available. Accordingly, we have ensured that both the raw datasets and the pre-processed smaller files have been deposited in the Electron Microscopy Public Image Archive (EMPIAR). The datasets are assigned accession number EMPIAR-12984 and will be publicly accessible upon publication of the manuscript, in accordance with EMPIAR release policies. We are also contacting the journal to confirm that reviewer access is enabled where possible, so that the files can be examined during the review process.

Minor points:

Line 41-42. ''Cellular resolution was achieved by enhanced tissue contrasting with phosphotunstic acid.'' The resolution of the scans is very good but not yet at the cellular level. Better to provide the actual voxel sizes here or amend ''cellular''.

Thank you for this comment, we have modified the sentence to tone down the claim on resolution.

Line 42/main text. Phosphotungstic is misspelled in several places throughout the manuscript.

We sincerely apologize for these grammatical mistakes and thank the reviewer for pointing them out. We have carefully checked the manuscript and corrected the errors.

Lines 68-69. ''Mouse, chicken, clawed frogs, and zebrafish are among the most commonly used species in developmental biology''. Add ''vertebrate'' before ''species in developmental biology''

We have clarified that these are all vertebrate species.

Lines 74-77. This sentence needs adjustment. There is a constantly growing number of evo-devo labs exploiting sea lamprey and catshark models. The advances of genome editing with CRISPR/Cas9 has allowed several labs to perform genome editing in lamprey and many studies on catshark gene expression, cell tracing etc.
We thank the Reviewer for this comment and agree that there has been substantial progress in recent years, including the adoption of techniques such as CRISPR/Cas9-mediated genome editing in lampreys and a growing number of studies on gene expression, lineage (dye) tracing, and developmental processes in catsharks. We also agree that the number of laboratories working with these organisms is steadily increasing.

The intent of the sentence in lines 74-77, however, was not to suggest that lampreys and catsharks are rarely used or that methodological advances have not occurred, but rather to emphasize that, despite this progress, studies on cyclostomes and chondrichthyans still represent a comparatively small proportion of developmental and evo-devo research when viewed alongside the extensive body of work on osteichthyan models. To avoid any unintended implication that research activity in these systems is limited, we have revised the wording of the sentence to explicitly acknowledge the recent expansion of work in lampreys and catsharks while retaining the original point regarding their relative underrepresentation.

"Notable progress in breeding and maintaining jawless vertebrates (cyclostomes – lamprey and hagfish) [18], has allowed their broader inclusion in modern evo-devo [19–21]. In addition, cartilaginous fishes (Chondrichthyes) such as elephant sharks (Callorhinchus milii) and bamboo sharks (Chiloscyllium sp.) are also emerging as key research species [22–24]. However, the vast majority of vertebrate evo-devo research remains mostly restricted to representatives of Osteichthyes, leaving cyclostomes and Chondrichthyes comparatively underrepresented."

Lines 93-94. Disagree to some extent, as the non-accessible one cell stage embryos makes genome editing very challenging in chondrichthyan species.

We agree and have now clarified that genome editing remains challenging in Chondrichthyes.

Lines 95-96. ''by immunofluorescence and in situ hybridization'' change to ''combined with immunofluorescence and in situ hybridization''

We have changed the wording following the Reviewer's suggestion.

Line 98. ''remains challenges'' change to '' remains challenging''

We thank the reviewer for noting this grammatical error, we have corrected it.

Line 99-100. ' as well as the lack of high-quality genome assemblies, which hinder the design of in situ hybridization probes''. Amend this phrase. With the projects like Vertebrate Genomes Project, the number of sequenced vertebrate genomes and their quality has increased dramatically. There are also numerous transcriptome datasets deposited in the GenBank even when the genome assembly is not complete.

We have removed the sentence following the Reviewer's suggestion.

Line 103-104. ''…we explored the use of X-ray-based methods, which allow imaging of thick samples at the centimeter scale.'' There are published studies applying X-ray synchrotron micro-tomography to catshark embryos. Add a citation for the recent study using synchrotron X-ray and iodine contrast on catshark late embryo (Mayeur et al 2024 MolBiolEvol).

We have added the reference following the Reviewer’s suggestion.

Line 108-109. ''The first one is its inability to effectively differentiate soft tissues due to insufficient differences in their X-ray attenuation coefficients.'' By placing the sample at different distances from the objective and applying propagation phase contrast, thick soft tissues can be imaged and soft tissues can be differentiated, this is actively used in Human Organ Atlas Project by applying hierarchical phase-contrast synchrotron tomography (Walsh et al 2021 Nature Methods).

We thank the Reviewer for this important clarification and agree that propagation-based phase-contrast synchrotron tomography can enable visualization and differentiation of soft tissues, including in relatively thick specimens, as demonstrated for example in hierarchical phase-contrast approaches such as those used in the Human Organ Atlas Project (Walsh et al., 2021).

The statement in Lines 108–109 (now 113-114), however, refers specifically to conventional absorption-based μCT, for which limited intrinsic soft-tissue contrast remains a general constraint and is one of the reasons contrast agents are widely used. While phase-contrast methods can in principle be applied to unstained embryonic material, their effectiveness depends strongly on imaging geometry, beam coherence, and the intrinsic density gradients within the specimen. In small embryonic tissues, where density differences between adjacent structures can be subtle, unstained phase-contrast imaging does not always provide sufficient anatomical discrimination for the types of analyses performed here, and staining approaches remain common practice [9–11].

To avoid any misunderstanding, we have revised the sentence to clarify that this limitation applies primarily to conventional absorption-based μCT and to acknowledge that phase-contrast methods can overcome this constraint under appropriate conditions, while keeping the introduction concise for a general readership.

Line 111-112. Some contrasting agents are also absorbed by hard tissues, but not PTA, this is important to mention here.

We have now stated that different contrasting agents exhibit distinct absorptions, as reflected in the resulting contrasted images, and explicitly mentioned that PTA is not absorbed by hard tissues (please see answer to point 3.2).

Line 122. ''(space and time)'' change to ''(volume and time)''

We have changed the wording following the Reviewer’s suggestion.

Line 123. ''often large'' change to ''non-transparent and thick''

We have changed the wording following the Reviewer’s suggestion.

Line 127. ''The provided dataset with superior quality and excellent contrast will benefit…'' Amend this phrase. The quality is based on resolution, the resolution above micron is not a superior resolution. A dataset at submicron resolution would produce a better quality dataset.
Contrast quality is not excellent, as it is dependent on individual sample preparation and PTA is also known to be less suitable contrast agent for some of the skeletal tissues. Contrast quality could also have been improved by changing propagation distances of imaged sample to detector, depending on the sample sizes.

We have toned down this statement.

“The provided dataset will benefit researchers interested in Chondrichthyes embryology and comparative evo-devo studies,”

Line 138. State the concentration of tricaine for overdose treatment

We have added the tricaine concentration used for the overdose treatment.

Line 141. ''increasing ethanol … steps'' change to ''increasing ethanol …concentrations in PBS''

We have changed the wording following the Reviewer's suggestion.

Line 142. ''the long ethanol steps'' change to '' the long incubation in ethanol''

We have changed the wording following the Reviewer's suggestion.

Line 148. ''Till scanning'' change to ''Until scanning''

We have changed the wording following the Reviewer's suggestion.

Line 152. Provide details of resin product number and manufacturer.

We apologize for the lack of information on the UV-light-sensitive resin used to seal the pipette tips. We have now incorporated this information into the text.

Line 160-173. Provide the details of the technical equipment i.e. camera, lens, scintillator used.

We have added the requested information to the main text.

Was propagation distance i.e. sample to detector distance changed between smaller and larger specimen to maximize a phase contrast effect?

Since all the samples were well contrasted and could be clearly visualized, we did not employ the phase-contrast scanning mode but instead used attenuation contrast. We first scanned a small and a large sample at the same sample-to-detector distance and observed that the quality and contrast of the resulting images were good for both. Hence, the sample-to-detector distance was not changed between smaller and larger specimens, and all were scanned with the same distance (80mm).

Describe if the beam was filtered and how.

The monochromatic beam was filtered using a double Christal monochromator with 1,1,1-silicon crystals to set a final photon energy of 20keV.

It is important to write if imaging was done over 360 degrees, and in a half or full acquisition mode.

Scanning was performed on full acquisition mode over 180° and an angular step of 0.059°. We have added this information to the main text.

Line 282. ''manipulate'' change to ''segment''

We have changed the wording following the Reviewer's suggestion.

Line 284. Add Mimics, and Dragonfly to the list of software.

We thank the reviewer for pointing these two additional software options and have added them to the list.

Figure 1A. What is dark/light color of eggshells linked to? This should be explained in the Figure legend.

We appreciate this very interesting question raised by the Reviewer. The small-spotted catshark eggshells are known to display a range of coloration from light beige to dark brown, as shown in Fig. 1A [12,13]. However, the underlying causes of this phenotypic variation remain unknown. In our breeding tanks, all adult catshark females were maintained under the same stable conditions (temperature, salinity, oxygenation, diet). It has been suggested that each female lays eggs of the same color [12]. Hence, eggshell coloration is likely linked to genetic factors rather than environmental ones. However, proving this

hypothesis would require additional investigation. Please note that eggshell color does not affect embryo development in any way [13]. We have incorporated this information in the Fig. 1 legend (see lines 618-621).

“Small-spotted catshark eggshells display a broad range of coloration from light beige to dark brown [89,90]. The underlying causes of this phenotypic variation remain unknown, although genetic factors have been suggested as contributing influences [89]. Importantly, eggshell pigmentation does not affect embryo development [90].”

Figure 1C. Is St15 embryo on the right mounted with rostral part down or up, add this information to the Figure legend.

We apologize for the lack of clarity regarding the embryonic axis and orientation of the embryos in the figure, we have clarified them in the figure as well as in the figure legends. For the Reviewer’s information, the St.15 embryo on the right is positioned dorsally with the rostral (anterior) part up.

Figure 2-4. Label corresponding developmental stages on the Figure, not only in the Figure legend.

We thank the reviewer for this suggestion and have now incorporated the corresponding embryonic stages into all the figures.

Supplementary Table 2. Provide embryo length and/or somite number for each sample.

We have incorporated the requested information on embryo length and somite number in Supplementary Table 2 (please see answer to point 6).

References

1. Lesciotto KM, Perrine SMM, Kawasaki M, Stecko T, Ryan TM, Kawasaki K, et al.. Phosphotungstic acid enhanced microCT: optimized protocols for embryonic and early postnatal mice. Dev Dyn. 2020; doi: 10.1002/dvdy.136.
2. Pétré M, Balcaen T, Schneidewind P, Mazy L, Pyka G, Fehervary H, et al.. Screening staining agents for contrast-enhanced microCT of vascular tissues: Assessing the effect on microstructural and mechanical properties. Tomography of Materials and Structures. 2024; doi: 10.1016/j.tmater.2024.100038.
3. Balint R, Lowe T, Shearer T. Optimal Contrast Agent Staining of Ligaments and Tendons for X-Ray Computed Tomography. PLOS ONE. Public Library of Science; 2016; doi: 10.1371/journal.pone.0153552.
4. Schmied C, Jambor HK. Effective image visualization for publications – a workflow using open access tools and concepts. F1000Research;
5. Matula J, Polakova V, Salplachta J, Tesarova M, Zikmund T, Kaucka M, et al.. Resolving complex cartilage structures in developmental biology via deep learning-based automatic segmentation of X-ray computed microtomography images. Sci Rep. Nature Publishing Group; 2022; doi: 10.1038/s41598-022-12329-8.
6. Kaucka M, Zikmund T, Tesarova M, Gyllborg D, Hellander A, Jaros J, et al.. Oriented clonal cell dynamics enables accurate growth and shaping of vertebrate cartilage. Bronner M, editor. eLife. eLife Sciences Publications, Ltd; 2017; doi: 10.7554/eLife.25902.
7. Kaucka M, Petersen J, Tesarova M, Szarowska B, Kastriti ME, Xie M, et al.. Signals from the brain and olfactory epithelium control shaping of the mammalian nasal capsule cartilage. White RM, editor. eLife. eLife Sciences Publications, Ltd; 2018; doi: 10.7554/eLife.34465.
8. Sunadome K, Erickson AG, Kah D, Fabry B, Adori C, Kameneva P, et al.. Directionality of developing skeletal muscles is set by mechanical forces. Nat Commun. Nature Publishing Group; 2023; doi: 10.1038/s41467-023-38647-7.
9. Matula J, Tesarova M, Zikmund T, Kaucka M, Adameyko I, Kaiser J. X-ray microtomography–based atlas of mouse cranial development. GigaScience. 2021; doi: 10.1093/gigascience/giab012.
10. Laznovsky J, Kavkova M, Helena Reis A, Robovska-Havelkova P, Maia LA, Krivanek J, et al.. Unveiling vertebrate development dynamics in frog Xenopus laevis using micro-CT imaging. GigaScience. 2024; doi: 10.1093/gigascience/giae037.

11. Metscher BD. MicroCT for developmental biology: A versatile tool for high-contrast 3D imaging at histological resolutions. Developmental Dynamics. 2009; doi: 10.1002/dvdy.21857.
12. Mellinger J. Egg-case diversity among dogfish, Scyliorhinus canicula (L.): a study of egg laying rate and nidamental gland secretory activity. Journal of Fish Biology. 1983; doi: 10.1111/j.1095-8649.1983.tb04728.x.
13. Koch A-K, Grunow B, Moritz T. Recommendations for scientific fish husbandry: Scyliorhinus canicula (Carcharhiniformes, Scyliorhinidae) – a model species for sharks. Bulletin of Fish Biology. 2025; doi: doi: 10.53188/BFB0012.

em Editorial Manager®

Cookie Preference Center

We use cookies which are necessary to make our site work. We may also use additional cookies to analyze, improve and personalize our content and your digital experience. For more information, see our Cookie Policy.

You may choose not to allow some types of cookies. However, blocking some types may impact your experience of our site and the services we are able to offer. See the different category headings below to find out more or change your settings.

Allow all

Manage Consent Preferences

Strictly Necessary Cookies

Always active

These cookies are necessary for the website to function and cannot be switched off in our systems. They are usually only set in response to actions made by you which amount to a request for services, such as setting your privacy preferences, logging in or filling in forms. You can set your browser to block or alert you about these cookies, but some parts of the site will not then work.

Functional Cookies

These cookies enable the website to provide enhanced functionality and personalisation. They may be set by us or by third party providers whose services we have added to our pages. If you do not allow these cookies then some or all of these services may not function properly.

Performance Cookies

These cookies allow us to count visits and traffic sources so we can measure and improve the performance of our site. They help us to know which pages are the most and least popular and see how visitors move around the site.

Targeting Cookies

These cookies may be set through our site by our advertising partners. They may be used by those companies to build a profile of your interests and show you relevant adverts on other sites. If you do not allow these cookies, you will experience less targeted advertising.

Cookie List

Clear

Apply Cancel
Consent Leg.Interest

Confirm my choices

Power
