# Supplementary material for: Synchrotron radiation micro-computed tomography of the small-spotted catshark embryonic development (Chondrichthyes: Scyliorhinus canicula)
Source: Gigascience. 2026 May 7;15:giag054. doi: 10.1093/gigascience/giag054 (PMC13240850; doi:10.1093/gigascience/giag054)
Supplement: giag054_Reviewer_2_Report_original_submission — Reviewer 2 -- 12/3/2025 [file giag054_reviewer_2_report_original_submission.pdf]

Reviewer Report

Title: Synchrotron radiation micro-computed tomography of the small-spotted catshark embryonic development (Chondrichthyes: Scyliorhinus canicula)

Version: Original Submission **Date:** 12/3/2025

Reviewer name: Reviewer 2

Reviewer Comments to Author:

This manuscript presents a high-resolution dataset of embryonic development in the small-spotted catshark (Scyliorhinus canicula), an emerging important model for studying vertebrate development and evolution. Using synchrotron radiation micro-computed tomography (SRÎ¼CT), 36 whole-embryo scans were obtained across key developmental stages (gastrulation to late organogenesis, stages 12-31). Enhanced tissue contrast with phosphotungstic acid enabled high-level resolution, allowing semi-automated reconstruction and pre-processing for 3D segmenetation of anatomical structures during the development. The dataset has a potential to provide a comprehensive resource for morphometric analyses and comparative evo-devo research, facilitating the identification of conserved and derived developmental processes in vertebrates.
This dataset has a great potential to enhance the evo-devo research on catshark, however, there are several major points that need to be addressed by the authors.
Major points
1.
It is important to make it clear that this is not the first attempt to image catshark embryos with a synchrotron radiation micro-computed tomography by using enhanced tissue contrast. For example, the recent paper by Mayeur et al (2024) described the 3D segmentation of the catshark late embryo sensory structures obtained by this technique using another contrast agent. Even though the authors cite this paper, it is not made clear in the text. However, I agree that this a first catshark embryo dataset spanning a large range of developmental stages that provides a great comparative potential.
2.
Catshark is an important vertebrate model, however it is very derived and during the evolution of vertebrates cartilaginous fishes lost perichondral, all major dermal and possibly even the endochondral bones (Ryll et al., 2014; Brazeau et al., 2020). This is important to add to the description of the species, to be able to describe for example the skeletal structures in the catshark embryo dataset.
3.
The information on image resolution, tissue enhancement properties of PTA and identified anatomical structures requires editing.
3.1 Authors state in several places in the text that they have acquired a dataset with a cellular resolution, however this would require a resolution below 1 micron. It is also visible from the virtual histology sections on Figure 3 and 4 for example, that the resolution in not at the cellular level. This statement should be changed. In addition, a virtual histology section on Figure 4D has a lower resolution comparing to other virtual histology sections presented and should be replaced, if possible.

3.2 It should be made clear in the text that PTA might not provide a good contrast for all types of tissues. In this dataset for example it is not well suitable for contrasting skeletal tissues, like muscle, and cartilage, which is visible on virtual histology sections on Figure 4.
Showing a few corresponding slides from a traditional histology for comparison would be valuable if authors would like to discuss PTA contrast as universal for all tissue types.
3.3 There are no labeled teeth, skin denticles, tendons, liver, reproductive organs in the Figures, despite that the authors have included these tissues in a long list of tissues and structures that can be analysed in this dataset (Lines 232-239). The suggestion is to edit this list of tissues and structures to include only the identified tissues in the dataset and all tissues mentioned should be also labeled in the Figures.
4.
Include mid-lateral virtual histology sections on Figure 3 and 4?
5.
Because the authors emphasize that ''The segmented 3D models can be directly compared between consecutive developmental stages to understand the complex morphogenetic processes during embryogenesis together with organ growth and shaping.'', by providing a 3D reconstruction of several consecutive stages showing organ growth and shaping changes (for example inner organs like heart and additional stages with segmented CNS) they would demonstrate this. The stages should be clearly indicated in the Figures and not only in the Figure legend, which will also make a comparative aspect more pronounced.
6.
Additional information is required for the growth series provided in Supplementary Table 2, this should include the length of the specimen and other important parameters like somite number.
7.
Author state that all data are publicly available for further exploration. However, there was no link available for reviewer for deposited raw or pre-processed small-size datasets. Therefore, the availability of all datasets described in Supplementary Table 2 could not be confirmed.
It is important that the authors make available the image stacks before and after processing i.e. of large and small size, since file size reduction might have resulted in the image quality reduction.
Minor points
Line 41-42
''Cellular resolution was achieved by enhanced tissue contrasting with phosphotunstic acid.''
The resolution of the scans is very good but not yet at the cellular level. Better to provide the actual voxel sizes here or amend ''cellular''.
Line 42/main text
Phosphotungstic is misspelled in several places throughout the manuscript.
Lines 68-69
''Mouse, chicken, clawed frogs, and zebrafish are among the most commonly used species in developmental biology''
Add ''vertebrate'' before ''species in developmental biology''
Lines 74-77
This sentence needs adjustment. There is a constantly growing number of evo-devo labs exploiting sea lamprey and catshark models. The advances of genome editing with CRISPR/Cas9 has allowed several

labs to perform genome editing in lamprey and many studies on catshark gene expression, cell tracing etc.
Lines 93-94
Disagree to some extent, as the non-accessible one cell stage embryos makes genome editing very challenging in chondrichthyan species.
Lines 95-96
''by immunofluorescence and in situ hybridization'' change to ''combined with immunofluorescence and in situ hybridization''
Line 98
''remains challenges'' change to '' remains challenging''
Line 99-100
'' as well as the lack of high-quality genome assemblies, which hinder the design of in situ hybridization probes''
Amend this phrase. With the projects like Vertebrate Genomes Project, the number of sequenced vertebrate genomes and their quality has increased dramatically. There are also numerous transcriptome datasets deposited in the GenBank even when the genome assembly is not complete.
Line 103-104
''…we explored the use of X-ray-based methods, which allow imaging of thick samples at the centimeter scale.''
There are published studies applying X-ray synchrotron micro-tomography to catshark embryos. Add a citation for the recent study using synchrotron X-ray and iodine contrast on catshark late embryo (Mayeur et al 2024 MolBiolEvol).
Line 108-109
''The first one is its inability to effectively differentiate soft tissues due to insufficient differences in their X-ray attenuation coefficients.''
By placing the sample at different distances from the objective and applying propagation phase contrast, thick soft tisseus can be imaged and soft tissues can be differentiated, this is actively used in Human Organ Atlas Project by applying hierarchical phase-contrast synchrotron tomography (Walsh et al 2021 Nature Methods).
Line 111-112
Some contrasting agents are also absorbed by hard tissues, but not PTA, this is important to mention here.
Line 122
''(space and time)'' change to ''(volume and time)''
Line 123
''often large'' change to ''non-transparent and thick''
Line 127
''The provided dataset with superior quality and excellent contrast will benefit…''
Amend this phrase. The quality is based on resolution, the resolution above micron is not a superior resolution. A dataset at submicron resolution would produce a better quality dataset.
Contrast quality is not excellent, as it is dependent on individual sample preparation and PTA is also known to be less suitable contrast agent for some of the skeletal tissues. Contrast quality could also

have been improved by changing propagation distances of imaged sample to detector, depending on the sample sizes.
Line 138
State the concentration of tricaine for overdose treatment
Line 141
''increasing ethanol … steps'' change to ''increasing ethanol …concentrations in PBS''
Line 142
''the long ethanol steps'' change to '' the long incubation in ethanol''
Line 148
''Till scanning'' change to ''Until scanning''
Line 152
Provide details of resin product number and manufacturer.
Line 160-173
Provide the details of the technical equipment i.e. camera, lens, scintillator used.
Was propagation distance i.e. sample to detector distance changed between smaller and larger specimen to maximize a phase contrast effect?
Describe if the beam was filtered and how.
It is important to write if imaging was done over 360 degrees, and in a half or full acquisition mode.
Line 282
''manipulate'' change to ''segment''
Line 284
Add Mimics, and Dragonfly to the list of software.
Figure 1A
What is dark/light color of eggshells linked to? This should be explained in the Figure legend.
Figure 1C
Is St15 embryo on the right mounted with rostral part down or up, add this information to the Figure legend.
Figure 2-4
Label corresponding developmental stages on the Figure, not only in the Figure legend.
Supplementary Table 2
Provide embryo length and/or somite number for each sample.

Methods

Are the methods appropriate to the aims of the study, are they well described, and are necessary controls included? Choose an item.

Conclusions

Are the conclusions adequately supported by the data shown? Choose an item.

Reporting Standards

Does the manuscript adhere to the journal's guidelines on minimum standards of reporting? Choose an item.

Choose an item.

Statistics

Are you able to assess all statistics in the manuscript, including the appropriateness of statistical tests used? Choose an item.

Quality of Written English

Please indicate the quality of language in the manuscript: Choose an item.

Declaration of Competing Interests

Please complete a declaration of competing interests, considering the following questions:

- Have you in the past five years received reimbursements, fees, funding, or salary from an organisation that may in any way gain or lose financially from the publication of this manuscript, either now or in the future?
- Do you hold any stocks or shares in an organisation that may in any way gain or lose financially from the publication of this manuscript, either now or in the future?
- Do you hold or are you currently applying for any patents relating to the content of the manuscript?
- Have you received reimbursements, fees, funding, or salary from an organization that holds or has applied for patents relating to the content of the manuscript?
- Do you have any other financial competing interests?
- Do you have any non-financial competing interests in relation to this paper?

If you can answer no to all of the above, write 'I declare that I have no competing interests' below. If your reply is yes to any, please give details below.

I declare that I have no competing interests

I agree to the open peer review policy of the journal. I understand that my name will be included on my report to the authors and, if the manuscript is accepted for publication, my named report including any attachments I upload will be posted on the website along with the authors' responses. I agree for my report to be made available under an Open Access Creative Commons CC-BY license (http://creativecommons.org/licenses/by/4.0/). I understand that any comments which I do not wish to be included in my named report can be included as confidential comments to the editors, which will not be published.

Choose an item.

To further support our reviewers, we have joined with Publons, where you can gain additional credit to further highlight your hard work (see: https://publons.com/journal/530/gigascience). On publication of this paper, your review will be automatically added to Publons, you can then choose whether or not to claim your Publons credit. I understand this statement.

Yes Choose an item.
